# Design, Synthesis and Herbicidal Activity of 5-(1-Amino-4-phenoxybutylidene)barbituric Acid Derivatives Containing an Enamino Diketone Motif

**DOI:** 10.3390/molecules30163445

**Published:** 2025-08-21

**Authors:** Ke Chen, Shumin Wang, Shuyue Fu, Yuxiao Zhang, Wei Gao, Jin Liu, Rui Liu, Kang Lei

**Affiliations:** 1 Department of Biotechnology, The University of Suwon, Hwaseong 18323, Gyeonggi-Do, Republic of Korea; chenke_sd@163.com; 2 School of Pharmaceutical Sciences and Food Engineering, Liaocheng University, Liaocheng 252059, China; 13686366329@163.com (S.W.); fsyue1213@163.com (S.F.); zhangyuxiao0515@163.com (Y.Z.); gaowei@lcu.edu.cn (W.G.); 3 Shandong Academy of Innovation and Development, Jinan 250101, China; lliujin7294@163.com

**Keywords:** barbituric acid, enamino diketone, herbicides, protoporphyrinogen IX oxidase

## Abstract

In continuation of our efforts to identify novel herbicide lead compounds, twenty new 5-(1-amino-4-phenoxybutylidene)barbituric acid derivatives containing an enamino diketone motif were synthesized and evaluated for their herbicidal activities. The greenhouse bioassay results indicated that several of the target compounds, including **BA-1**, **BA-2**, **BA-5**, **BA-18**, and **BA-20**, exhibited notable post-emergence herbicidal activity, with sum inhibition rates exceeding 70% at a dosage of 150 g ha^−1^, which was superior to that of the commercial herbicide flumiclorac-pentyl (FP). The structure–activity relationship analysis demonstrated that the steric and electronic effects of the R group, as well as the lipophilicity of the target compounds, significantly influenced herbicidal activity. Among these, **BA-1** was identified as a promising herbicide lead compound due to its high total herbicidal efficacy, broad-spectrum activity, and favorable crop safety profile. Molecular simulation studies indicated that **BA-1** binds effectively to *Nicotiana tabacum* protoporphyrinogen IX oxidase (*Nt*PPO), suggesting its potential as a novel PPO inhibitor. This study highlights **BA-1** as a promising lead compound for the development of novel PPO-inhibiting herbicides.

## 1. Introduction

As essential tools in the prevention and control of weeds in agricultural production, herbicides play a crucial role in maintaining stable agricultural yields and enhancing economic efficiency [1,2,3]. It is estimated that the absence of herbicides would result in a 34% reduction in global crop yields due to weed infestation [4]. However, the extensive and indiscriminate use of herbicides has led to various adverse effects, including significant environmental issues and the development of herbicide-resistant weed species [5,6,7,8,9]. The development of new herbicides encounters considerable challenges, such as an extended development cycle, substantial financial investment, and low success rates [10]. Identifying novel lead scaffolds for herbicides is still a key step in the development of new pesticide agents [11,12,13,14].

**Figure 1:**
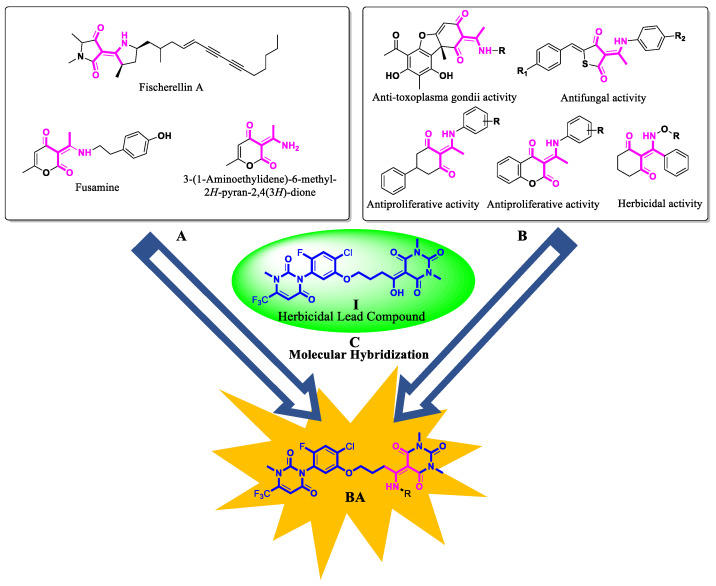
Design of target compound **BA** based on the molecular hybridization strategy. (**A**) Natural products with enamino diketone moiety; (**B**) Bioactive compounds with enamino diketone moiety; (**C**) Reported lead compound **I**.

The enamino diketone represents a fundamental structural motif present in some natural products (Figure 1A) [15,16]. Meanwhile, compounds containing the enamino diketone motif (Figure 1B) have demonstrated significant utility in the pharmaceutical and agrochemical fields due to their antimicrobial [17,18], antibacterial [19,20,21], anticancer [22,23,24,25,26], herbicidal [27,28,29], antifungal [30,31,32], and anti-*toxoplasma gondii* activities [33]. The distinctive structural and biological properties of the enamino diketone motif underscore its potential utility in the development of novel pesticidal agents.

Recently, during our investigation into novel compounds with potential herbicidal applications, we identified a promising lead structure, designated as compound **I** (Figure 1C) [34,35]. Notably, compound **I** contains a diketone moiety that is structurally similar to those found in enamino diketone derivatives. Based on this observation, we hypothesized that converting the carbonyl group of the acyl moiety in compound **I** into an imine, followed by isomerization to the enamine form, could facilitate the construction of compounds featuring the naturally occurring enamino diketone motif. Such structural modifications are expected to possess herbicidal activity. Accordingly, twenty new 5-(1-amino-4-phenoxybutylidene)barbituric acid derivatives (**BA**) containing an enamino diketone motif were designed, synthesized, and their herbicidal activities were evaluated. This report presents the synthesis, structure-activity relationship (SAR) analysis, and molecular simulation studies of **BA derivatives**.

## 2. Results and Discussion

### 2.1. Chemistry

**Scheme 1:**
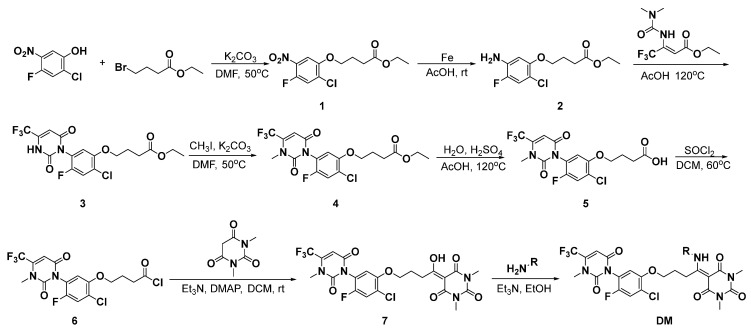
General synthetic pathway for the target compounds **BA-1**~**BA-20**.

**Figure 2:**
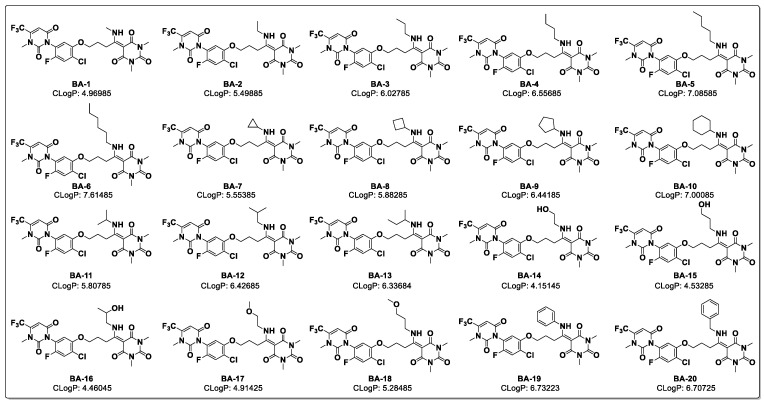
The structures of target compounds **BA-1**~**BA-20**.

The target compounds **BA-1**~**BA-20** were prepared through an eight-step synthetic pathway, as illustrated in Scheme 1, and their chemical structures are presented in Figure 2. The calculated oil/water partition coefficients (Clog *P*) are predicted using ChemBioDraw 14.0. The synthetic route commenced with a base-promoted nucleophilic substitution reaction between 2-chloro-4-fluoro-5-nitrophenol and ethyl 4-bromobutanoate. Briefly, the 2-chloro-4-fluoro-5-nitrophenol reacted with ethyl 4-bromobutanoate in DMF by using K_2_CO_3_ as a base to generate intermediate **1**. The nitro group in intermediate **1** was then chemically reduced by using iron powder in glacial acetic acid at room temperature to yield intermediate **2**, which subsequently underwent a cyclization reaction with ethyl (*Z*)-3-(3,3-dimethylureido)-4,4,4-trifluorobut-2-enoate to produce intermediate **3**. Methylation of compound **3** with iodomethane produced intermediate **4**, which was hydrolyzed by heating under acidic conditions to form carboxylic acid **5**. Carboxylic acid **5** was readily converted to the corresponding acid chloride **6** on treatment with thionyl chloride. The key intermediate **7** could then be smoothly synthesized by reaction of acid chloride **6** with barbituric acid. Finally, the target compounds **BA-1**~**BA-20** were obtained in yields ranging from 16% to 93% through condensation reactions between intermediate **7** and corresponding amines in ethanol. The structures of all the target compounds were identified using ^1^H and ^13^C NMR spectroscopy and HRMS.

### 2.2. Herbicidal Activity and SAR

**Figure 3:**
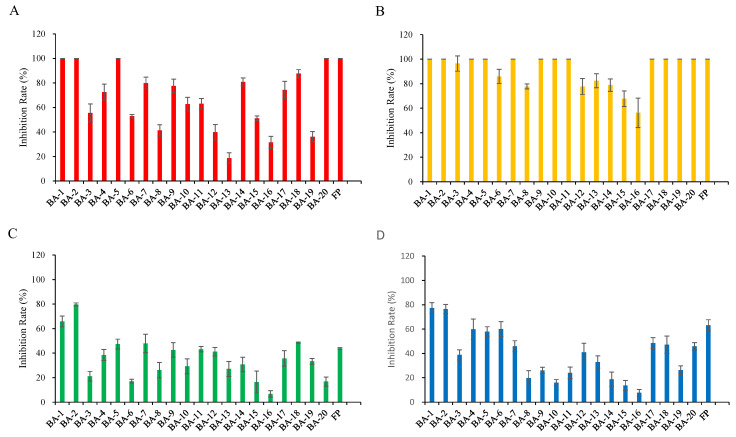
Effects (% inhibition) of the target compounds **BA-1**~**BA-20** on the loss of plant weight at a dosage of 150 g ha^−1^ under the post-emergence condition; (**A**) *B. campestris*; (**B**) *A. retroflexus*; (**C**) *E. crus-galli*; (**D**) *D. sanguinalis*.

**Figure 4:**
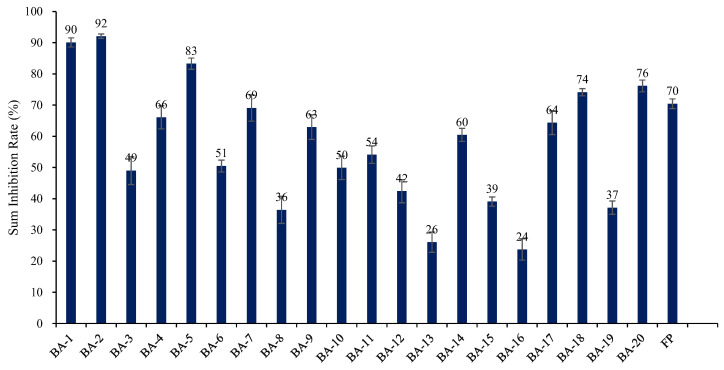
Total effects (% inhibition) of the target compounds **BA-1**~**BA-20** on the loss of plant weight at a dosage of 150 g ha^−1^ under the post-emergence condition.

To evaluate the herbicidal activity of the newly synthesized target compounds **BA-1**~**BA-20**, greenhouse trials were conducted at a post-emergence application rate of 150 g ha^−1^. Commercial herbicide flumiclorac-pentyl (FP) was employed as the positive control. As observed in Figure 3, most of the target compounds exhibited moderate to good herbicidal activity against the four tested weeds. Among them, target compounds **BA-1**, **BA-2**, **BA-5**, and **BA-20** demonstrated complete inhibition against *Brassica campestris* (Figure 3A). Apart from **BA-3**, **BA-6**, **BA-8**, and **BA-12**~**BA-16**, all other target compounds demonstrated complete inhibitory activity against *Amaranthus tricolor*, showing efficacy comparable to that of the commercial herbicide FP (Figure 3B). Furthermore, it was found that some of the newly synthesized target compounds, such as **BA-1** and **BA-2**, exhibited stronger herbicidal activity against *Echinochloa crus-galli* (Figure 3C) and *Digitaria sanguinalis* (Figure 3D) than the commercial herbicide FP. To better understand the herbicidal activity, the total effects of the target compounds **BA-1**~**BA-20** on the loss of plant weight at the dosage of 150 g ha^−1^ were calculated, and the results shown in Figure 4. It was easy to observe that some of the target compounds, such as **BA-1**, **BA-2**, **BA-5**, **BA-18**, and **BA-20**, demonstrated higher sum inhibition rate (SIR) than that of the commercial herbicide FP. These promising results indicated that the series target compound **BA** was effectively designed.

Based on the total herbicidal inhibition rate at the dosage of 150 g ha^−1^, the structure-activity relationship (SAR) of the target compounds was investigated. It was found that, among the target compounds containing straight-chain alkyl groups (i.e., compounds **BA-1**~**BA-6**), **BA-1** (R = Me) and **BA-2** (R = Et) exhibited higher SIR than those with longer alkyl chains (i.e., compounds **BA-3**~**BA-6**). When cycloalkyl groups (i.e., compounds **BA-7**~**BA-10**) or branched-chain alkyl groups (i.e., compounds **BA-11** and **BA-13**) were introduced on the nitrogen atom of target compounds, the target compounds (excluding **BA-7**) exhibited lower SIR than the corresponding straight-chain compounds. For instance, the target compound **BA-4** (R = *n*-butyl) exhibited a total inhibitory effect against the four tested weeds with an SIR of 66%, which is higher than that of **BA-8** (SIR = 36%), **BA-12** (SIR = 42%) and **BA-13** (SIR = 26%). Based on the above analysis, it was speculated that the introduction of smaller substituents on the nitrogen atoms of the target compounds may enhance herbicidal activity. However, through a comparative analysis of the herbicidal activities of compounds **BA-3**~**BA-6** and **BA-8**~**BA-10**, it was found that the SIR increased with the extension of carbon chain and expansion of carbon ring, and the trend terminated at compound **BA-5** (R = pentyl) and **BA-8** (R = cyclopentyl), respectively. This finding contradicts the aforementioned speculation, suggesting that the steric effect of the R group is not the sole factor influencing herbicidal activity.

Previous studies have revealed that lipophilicity is an important parameter affecting the absorption of pesticides by plants, and appropriate lipophilicity is essential for biological activity [36]. Thus, lipophilicity as Clog *P* was predicted by ChemBioDraw 14.0 as shown in Figure 2. It was found that there are some extent correlations between herbicidal activity and lipophilicity of the target compounds. For instance, for the target compounds **BA-3**~**BA-6**, as the Clog *P* value increases, the herbicidal activities of the target compounds **BA-3** (Clog *P* = 6.0, SIR = 49%), **BA-4** (Clog *P* = 6.5, SIR = 66%), and **BA-5** (Clog *P* = 7.0, SIR = 83%) progressively enhance; With the continuous increase in the Clog *P* value, target compound **BA-6** (Clog *P* = 7.6, SIR = 51%) exhibited lower total herbicidal inhibition efficiency than compound **BA-5**, which may be caused by the poor uptake and translocation of **BA-6** in plants due to relatively high lipophilicity. A similar trend is also evident in target compounds **BA-8**~**BA-10**. Furthermore, the introduction of hydroxyl groups onto the alkyl chains attached to the nitrogen atoms of the target compounds **BA-2** and **BA-3** resulted in reduced herbicidal activity among compounds **BA-14** (Clog *P* = 4.1, SIR = 60%), **BA-15** (Clog *P* = 4.5, SIR = 39%), and **BA-16** (Clog *P* = 4.4, SIR = 24%). Therefore, it can be concluded that the introduction of hydrophilic substituents on the nitrogen atom of the target compounds does not enhance herbicidal activity. The herbicidal activity of compounds **BA-17** and **BA-18** supported this hypothesis. When the hydroxyl group in **BA-14** and **BA-15** was replaced by a methoxy group, the herbicidal activity of **BA-17** (Clog *P* = 4.9, SIR = 64%) and **BA-18** (Clog *P* = 5.2, SIR = 74%) was enhanced. These findings indicate that increasing the lipophilicity of target compounds to a certain extent is beneficial to improving the herbicidal activity; However, if the Clog *P* value is excessively low or high, the herbicidal efficiency decreases.

When a benzene ring was introduced on the nitrogen atom of target compounds, **BA-19** (SIR = 37%) exhibited lower herbicidal activity compared to **BA-6** (SIR = 51%). However, inserting a straight-chain alkyl group between the benzene ring and the nitrogen atom significantly enhanced herbicidal activity, as demonstrated by compound **BA-20** (SIR = 76%) relative to **BA-19**. These results suggest that, in addition to steric effects and lipophilicity, electronic effects also have a crucial influence on the herbicidal activities of these compounds.

To further investigate the herbicidal activity of the target compounds, five compounds, i.e., **BA-1**, **BA-2**, **BA-5**, **BA-18**, and **BA-20**, that demonstrated a higher SIR compared to the commercial herbicide FP, were selected for dose–response analysis through serial two-fold dilutions. As shown in Table 1, reducing the dosage from 75 to 18.8 g ha^−1^ resulted in a progressive decline in the herbicidal activity of all tested compounds. Moreover, the tested compounds demonstrated greater efficacy against dicotyledonous weed species compared to monocotyledonous plant species. For instance, compound **BA-1** achieved herbicidal activities of 84% and 100% against *B. campestris* and *A. tricolor*, respectively, at a dosage of 18.8 g ha^−1^, whereas the inhibition rates against *E. crus-galli* and *D. sanguinalis* were only 18% and 11%, respectively. Notably, among the evaluated compounds, **BA-1**, **BA-2**, and **BA-18** exhibited strong herbicidal activity even at a dosage of 18.8 g ha^−1^, achieving SIRs of 64%, 51%, and 50%, respectively, which exceeded the commercial herbicide FP. These findings suggest that these newly synthesized compounds hold promise as potential lead candidates for the development of novel herbicides.

### 2.3. Herbicidal Spectrum and Crop Safety of Target Compound ***BA-1***

**Figure 5:**
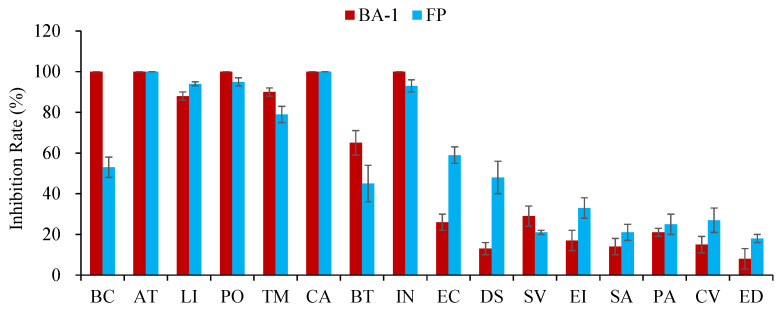
Herbicidal spectrum testing of compound **BA-1** under the post-emergence conditions at the dosage of 37.5 g ha^−1^; Abbreviation: BC: *B. campestris*, AT: *A. tricolor*, LI: *L. indica*, PO: *P. oleracea*, TM: *T. mongolicum*, CA: *C. album*, BT: *Bidens tripartita*, IN: *I. nil*, EC: *E. crus-galli*, DS: *D. sanguinalis*, SV: *Setaria viridis*, EI: *Eleusine indica*, SA: *Spartina alterniflora*, PA: *Pennisetum alopecuroides*, CV: *Chloris virgata*, and ED: *Elymus dahuricus*.

Based on the results of the above two rounds of screening, compound **BA-1** was selected for further evaluation of its herbicidal spectrum due to its pronounced herbicidal activity. As shown in Figure 5, compound **BA-1** exhibited substantial herbicidal efficacy against the majority of tested dicotyledonous weed species, including *B. campestris*, *A. tricolor*, *Lactuca indica*, *Portulaca oleracea*, *Taraxacum mongolicum*, *Chenopodium album*, and *Ipomoea nil*, with inhibition rates exceeding 85%. In contrast, compound **BA-1** displayed relatively limited inhibitory effects against the monocotyledonous plant species tested, indicating a degree of selectivity toward dicotyledonous weeds. Furthermore, a crop safety assessment was conducted to evaluate the potential application of **BA-1** as an herbicide. The results presented in Table 2 indicated that among the four tested crops treated with **BA-1**, *Triticum aestivum* and *Zea mays* exhibited relatively high tolerance, whereas *Gossypium hirsutum* and *Glycine max* were more susceptible under the same experimental conditions, with injury rates of 44% and 47%, respectively. Collectively, these findings suggest that compound **BA-1** holds promise for development as a post-emergence herbicide for the control of dicotyledonous weeds in fields of *T. aestivum* and *Z. mays*.

### 2.4. Molecular Simulation Analysis of ***BA-1***

**Figure 6:**
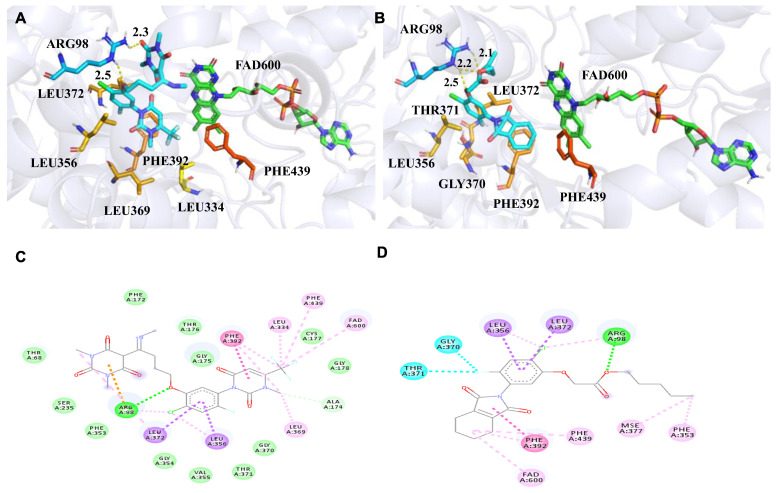
Docking analysis between **BA-1** or FP and *Nt*PPO. (**A**) 3D of molecular docking between **BA-1** and *Nt*PPO; (**B**) 3D of molecular docking between FP and *Nt*PPO; (**C**) 2D of molecular docking between **BA-1** and *Nt*PPO; (**D**) 2D of molecular docking between FP and *Nt*PPO.

**Figure 7:**
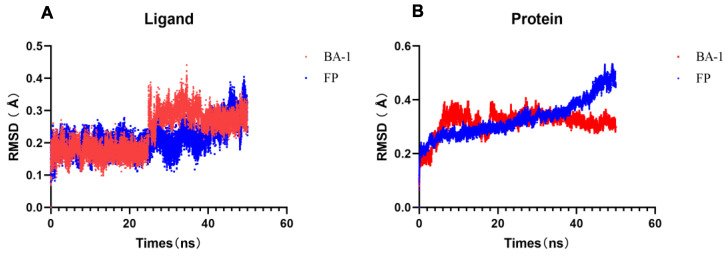
RMSD of protein (**A**) and ligand (**B**) plots of protein–ligand complexes during 50 ns MD simulations.

Given that **BA-1** possesses pyrimidinedione moiety like the commercial herbicide saflufenacil, it is reasonable to hypothesize that **BA-1** may function as a protoporphyrinogen IX oxidase (PPO) inhibitor. To verify the above speculation, a molecular docking study was performed to confirm the interactions between **BA-1** or FP and *Nicotiana tabacum* PPO (*Nt*PPO). As shown in Figure 6, **BA-1** and FP had some identical interacting amino acid residues, such as Arg98, Leu356, Leu372, and Phe392. The pyrimidinedione ring of **BA-1** (Figure 6A,C) and the maleimide ring of FP (Figure 6B,D) engage in π–π stacking interactions with Phe392, respectively. Additionally, the benzene rings of **BA-1** and FP participate in π–σ interactions with Leu356 and Leu372, respectively, which are conserved residues among *Nt*PPO enzymes. Furthermore, Arg98 forms a hydrogen bond with **BA-1**, like the interaction observed with FP. Notably, the barbiturate structure in **BA-1** shapes π-cation stacking interactions with Arg98 to enhance the binding with *Nt*PPO active cavity. To assess the binding stability of the complex formed between **BA-1** and *Nt*PPO, 50 ns molecular dynamics (MD) simulations were conducted using the GROMACS 2023.3 software package. FP was employed as the positive control. As illustrated in Figure 7, both the ligand (Figure 7A) and the protein structure (Figure 7B) exhibited stable conformational behavior throughout the 50 ns simulation period, with root-mean-square deviation (RMSD) fluctuations consistently below 0.5 Å. These findings indicate that compound **BA-1** forms a stable interaction with *Nt*PPO.

## 3. Materials and Methods

### 3.1. Chemical Synthesis Procedures

Detailed synthetic procedures for the intermediates and target compounds are described below; however, the reaction yields have not been optimized.

#### 3.1.1. Synthesis of Intermediates **1**–**7**

The intermediates **1**–**7** was synthesized according to a previously reported method [11].

#### 3.1.2. General Procedure for the Synthesis of Target Compounds **BA-1** to **BA-20**

The target compounds **BA-1**~**BA-20** were synthesized according to a previously reported method [34,35]. A representative example for the synthesis of **BA-1** is described as follows: The intermediate **7** (281 mg, 0.5 mmol) was dissolved in 10 mL of ethanol followed by the addition of methylamine hydrochloride (40.8 mg, 0.6 mmol), and the mixture was refluxed at 90 °C for 12 h. After cooling to room temperature, the resulting solid was collected by filtration, recrystallized with ethanol to give target compound **BA-1** as a white solid (84.5 mg, yield: 29.4%). The target compounds **BA-2**~**BA-20** were prepared by a procedure like that for **BA-1**. The spectrum of ^1^H NMR, ^13^C NMR, and HRMS of all target compounds **BA-1**~**BA-20** can be found in Supplementary Materials (Figures S1–S60), and the data as follows:

5-(4-(2-Chloro-4-fluoro-5-(3-methyl-2,6-dioxo-4-(trifluoromethyl)-3,6-dihydropyrimidin-1(2*H*)-yl)phenoxy)-1-(methylamino)butylidene)-1,3-dimethylpyrimidine-2,4,6(1*H*,3*H*,5*H*)-trione (**BA-1**): white solid, yield 29.4%, m.p. 191–194 °C; ^1^H NMR (500 MHz, CDCl_3_) *δ* 12.57 (s, 1H), 7.32 (d, *J* = 8.8 Hz, 1H), 6.80 (d, *J* = 6.3 Hz, 1H), 6.38 (s, 1H), 4.13 (t, *J* = 5.4 Hz, 2H), 3.57 (s, 3H), 3.41–3.35 (m, 2H), 3.32 (s, 3H), 3.30 (s, 3H), 3.26 (d, *J* = 5.2 Hz, 3H), 2.16 (m, 2H); ^13^C NMR (125 MHz, CDCl_3_) *δ* 177.25 (s), 166.69 (s), 162.54 (s), 159.94 (s), 152.43 (s), 151.45 (d, *J* = 248.0 Hz), 151.34 (s), 151.01 (d, *J* = 2.4 Hz), 150.69 (s), 141.79 (q, *J* = 34.6 Hz), 124.53 (d, *J* = 9.4 Hz), 120.67 (d, *J* = 14.9 Hz), 119.37 (q, *J* = 275.4 Hz), 118.49 (d, *J* = 24.1 Hz), 113.53 (s), 103.11 (q, *J* = 5.4 Hz), 89.93 (s), 68.88 (s), 32.74 (q, *J* = 3.4 Hz), 30.06 (s), 27.93 (s), 27.65 (s), 26.65 (s), 26.61 (s); HRMS, *m*/*z* calcd. for C_23_H_23_ClF_4_N_5_O6^+^ [M + H]^+^ 576.12675, found 576.12628.

5-(4-(2-Chloro-4-fluoro-5-(3-methyl-2,6-dioxo-4-(trifluoromethyl)-3,6-dihydropyrimidin-1(2*H*)-yl)phenoxy)-1-(ethylamino)butylidene)-1,3-dimethylpyrimidine-2,4,6(1*H*,3*H*,5*H*)-trione (**BA-2**): white solid, yield 31%, m.p. 212–215 °C; ^1^H NMR (500 MHz, CDCl_3_) *δ* 12.56 (s, 1H), 7.33 (d, *J* = 8.8 Hz, 1H), 6.80 (d, *J* = 6.3 Hz, 1H), 6.38 (s, 1H), 4.12 (t, *J* = 5.4 Hz, 2H), 3.70–3.61 (m, 2H), 3.57 (s, 3H), 3.39–3.29 (m, 8H), 2.16 (m, 2H), 1.38 (t, *J* = 7.3 Hz, 3H); ^13^C NMR (125 MHz, CDCl_3_) *δ* 176.03 (s), 166.73 (s), 162.60 (s), 159.94 (s), 151.44 (d, *J* = 248.0 Hz), 151.37 (s), 151.03 (d, *J* = 2.2 Hz), 150.70 (s), 141.80 (q, J = 34.6 Hz), 124.49 (d, *J* = 9.5 Hz), 120.69 (d, *J* = 14.9 Hz), 119.37 (q, *J* = 275.2 Hz), 118.49 (d, *J* = 24.1 Hz), 113.51 (s), 103.11 (q, *J* = 5.6 Hz), 89.60 (s), 68.91 (s), 38.38 (s), 32.74 (q, *J* = 3.2 Hz), 27.93 (s), 27.65 (s), 27.11 (s), 26.79 (s), 14.90 (s); HRMS, *m*/*z* calcd. for C_24_H_25_ClF_4_N_5_O_6_^+^ [M + Na]^+^ 590.14240, found 590.14178.

5-(4-(2-Chloro-4-fluoro-5-(3-methyl-2,6-dioxo-4-(trifluoromethyl)-3,6-dihydropyrimidin-1(2*H*)-yl)phenoxy)-1-(propylamino)butylidene)-1,3-dimethylpyrimidine-2,4,6(1*H*,3*H*,5*H*)-trione (**BA-3**): white solid, yield 93%, m.p. 225–227 °C; ^1^H NMR (500 MHz, CDCl_3_) *δ* 12.63 (s, 1H), 7.33 (d, *J* = 8.8 Hz, 1H), 6.80 (d, *J* = 6.3 Hz, 1H), 6.38 (s, 1H), 4.12 (t, *J* = 5.4 Hz, 2H), 3.62–3.53 (m, 5H), 3.40–3.28 (m, 8H), 2.16 (m, 2H), 1.81–1.71 (m, 2H), 1.06 (t, *J* = 7.4 Hz, 3H); ^13^C NMR (125 MHz, CDCl_3_) *δ* 176.15 (s), 166.74 (s), 162.58 (s), 159.92 (s), 151.42 (d, *J* = 248.0 Hz), 151.36 (s), 151.01 (d, *J* = 2.4 Hz), 150.68 (s), 141.77 (q, *J* = 34.4 Hz), 124.46 (d, *J* = 9.4 Hz), 120.69 (d, *J* = 14.9 Hz), 119.37 (q, *J* = 275.3 Hz), 118.46 (d, *J* = 24.1 Hz), 113.48 (s), 103.10 (q, *J* = 5.4 Hz), 89.63 (s), 68.86 (s), 45.15 (s), 32.73 (q, *J* = 3.5 Hz), 27.92 (s), 27.65 (s), 27.07 (s), 26.81 (s), 22.88 (s), 11.40 (s); HRMS, *m*/*z* calcd. for C_25_H_27_ClF_4_N_5_O_6_^+^ [M + H]^+^ 604.15805, found 604.15729.

5-(1-(Butylamino)-4-(2-chloro-4-fluoro-5-(3-methyl-2,6-dioxo-4-(trifluoromethyl)-3,6-dihydropyrimidin-1(2*H*)-yl)phenoxy)butylidene)-1,3-dimethylpyrimidine-2,4,6(1*H*,3*H*,5*H*)-trione (**BA-4**): white solid, yield 75%, m.p. 217–219 °C; ^1^H NMR (500 MHz, CDCl_3_) *δ* 12.61 (s, 1H), 7.32 (d, *J* = 8.8 Hz, 1H), 6.80 (d, *J* = 6.3 Hz, 1H), 6.38 (s, 1H), 4.12 (t, *J* = 5.4 Hz, 2H), 3.60 (dd, *J* = 14.6, 8.9 Hz, 5H), 3.40–3.28 (m, 8H), 2.15 (m, 2H), 1.78–1.67 (m, 2H), 1.47 (m, 2H), 0.98 (t, *J* = 7.3 Hz, 3H); ^13^C NMR (125 MHz, CDCl_3_) *δ* 176.09 (s), 166.74 (s), 162.58 (s), 159.92 (s), 151.43 (d, *J* = 248.1 Hz), 151.36 (s), 151.02 (d, *J* = 2.5 Hz), 150.69 (s), 141.78 (q, *J* = 34.6 Hz), 124.47 (d, *J* = 9.4 Hz), 120.69 (d, *J* = 14.8 Hz), 119.37 (q, *J* = 275.3 Hz), 118.46 (d, *J* = 24.2 Hz), 113.49 (s), 103.10 (q, *J* = 5.3 Hz), 89.63 (s), 68.89 (s), 43.28 (s), 32.73 (q, *J* = 3.4 Hz), 31.54 (s), 27.92 (s), 27.66 (s), 27.08 (s), 26.84 (s), 20.05 (s), 13.69 (s); HRMS, *m*/*z* calcd. for C_26_H_29_ClF_4_N_5_O_6_^+^ [M + H]^+^ 618.17370, found 618.17316.

5-(4-(2-Chloro-4-fluoro-5-(3-methyl-2,6-dioxo-4-(trifluoromethyl)-3,6-dihydropyrimidin-1(2*H*)-yl)phenoxy)-1-(pentylamino)butylidene)-1,3-dimethylpyrimidine-2,4,6(1*H*,3*H*,5*H*)-trione (**BA-5**): white solid, yield 36%, m.p. 205–207 °C; ^1^H NMR (500 MHz, CDCl_3_) *δ* 12.61 (s, 1H), 7.33 (d, *J* = 8.8 Hz, 1H), 6.80 (d, *J* = 6.3 Hz, 1H), 6.38 (s, 1H), 4.12 (t, *J* = 5.4 Hz, 2H), 3.59 (dd, *J* = 11.4, 5.7 Hz, 5H), 3.41–3.28 (m, 8H), 2.15 (m, 2H), 1.79–1.68 (m, 2H), 1.46–1.34 (m, 4H), 0.93 (t, *J* = 7.0 Hz, 3H); ^13^C NMR (125 MHz, CDCl_3_) *δ* 176.06 (s), 166.73 (s), 162.60 (s), 159.93 (s), 151.43 (d, *J* = 248.0 Hz), 151.37 (s), 151.02 (d, *J* = 2.6 Hz), 150.69 (s), 124.47 (d, *J* = 9.3 Hz), 120.69 (d, *J* = 14.8 Hz), 119.38 (q, *J* = 275.4 Hz), 118.46 (d, *J* = 24.4 Hz), 113.49 (s), 103.10 (q, *J* = 5.4 Hz), 89.62 (s), 68.88 (s), 43.56 (s), 32.73 (q, *J* = 3.2 Hz), 29.24 (s), 28.91 (s), 27.92 (s), 27.66 (s), 27.07 (s), 26.85 (s), 22.28 (s), 13.88 (s); HRMS, *m*/*z* calcd. for C_27_H_31_ClF_4_N_5_O_6_^+^ [M + H]^+^ 632.18935, found 632.18842.

5-(4-(2-Chloro-4-fluoro-5-(3-methyl-2,6-dioxo-4-(trifluoromethyl)-3,6-dihydropyrimidin-1(2*H*)-yl)phenoxy)-1-(hexylamino)butylidene)-1,3-dimethylpyrimidine-2,4,6(1*H*,3*H*,5*H*)-trione (**BA-6**): white solid, yield 90%, m.p. 201–203 °C; ^1^H NMR (500 MHz, CDCl_3_) *δ* 12.61 (s, 1H), 7.33 (d, *J* = 8.8 Hz, 1H), 6.80 (d, *J* = 6.3 Hz, 1H), 6.38 (s, 1H), 4.12 (t, *J* = 5.4 Hz, 2H), 3.59 (dd, *J* = 11.8, 6.1 Hz, 5H), 3.39–3.27 (m, 8H), 2.15 (m, 2H), 1.72 (m, 2H), 1.47–1.39 (m, 2H), 1.37–1.30 (m, 4H), 0.91 (dd, *J* = 9.2, 4.8 Hz, 3H); ^13^C NMR (125 MHz, CDCl_3_) *δ* 176.04 (s), 166.72 (s), 162.58 (s), 159.91 (s), 151.42 (d, *J* = 248.0 Hz), 151.36 (s), 151.01 (d, *J* = 2.5 Hz), 150.68 (s), 141.76 (q, *J* = 34.5 Hz), 124.45 (d, *J* = 9.3 Hz), 120.69 (d, *J* = 14.8 Hz), 119.37 (q, *J* = 275.5 Hz), 118.44 (d, *J* = 24.1 Hz), 113.49 (s), 103.09 (q, *J* = 5.4 Hz), 89.60 (s), 68.88 (s), 43.58 (s), 32.71 (q, *J* = 3.1 Hz), 31.32 (s), 29.52 (s), 27.90 (s), 27.64 (s), 27.07 (s), 26.83 (s), 26.48 (s), 22.44 (s), 13.96 (s); HRMS, *m*/*z* calcd. for C_28_H_33_ClF_4_N_5_O_6_^+^ [M + H]^+^ 646.20500, found 646.20422.

5-(4-(2-Chloro-4-fluoro-5-(3-methyl-2,6-dioxo-4-(trifluoromethyl)-3,6-dihydropyrimidin-1(2*H*)-yl)phenoxy)-1-(cyclopropylamino)butylidene)-1,3-dimethylpyrimidine-2,4,6(1*H*,3*H*,5*H*)-trione (**BA-7**): white solid, yield 87%, m.p. 211–214 °C; ^1^H NMR (500 MHz, CDCl_3_) *δ* 12.49 (s, 1H), 7.31 (d, *J* = 8.8 Hz, 1H), 6.81 (d, *J* = 6.3 Hz, 1H), 6.38 (s, 1H), 4.16 (t, *J* = 5.5 Hz, 2H), 3.62–3.51 (m, 5H), 3.31 (s, 3H), 3.30 (s, 3H), 3.11–3.04 (m, 1H), 2.22 (m, 2H), 1.10–1.01 (m, 2H), 0.85–0.78 (m, 2H); ^13^C NMR (125 MHz, CDCl_3_) *δ* 178.48 (s), 166.60 (s), 162.31 (s), 159.92 (s), 151.38 (d, *J* = 248.1 Hz), 151.27 (s), 151.06 (d, *J* = 2.4 Hz), 150.68 (s), 141.77 (q, *J* = 34.3 Hz), 124.38 (d, *J* = 9.3 Hz), 120.69 (d, *J* = 14.9 Hz), 119.37 (q, *J* = 275.4 Hz), 118.47 (d, *J* = 24.3 Hz), 113.50 (s), 103.09 (q, *J* = 5.4 Hz), 89.94 (s), 69.07 (s), 32.73 (d, *J* = 3.5 Hz), 27.92 (s), 27.63 (s), 26.83 (s), 25.62 (s), 8.09 (s); HRMS, *m*/*z* calcd. for C_25_H_25_ClF_4_N_5_O_6_^+^ [M + H]^+^ 602.14240, found 602.14185.

5-(4-(2-Chloro-4-fluoro-5-(3-methyl-2,6-dioxo-4-(trifluoromethyl)-3,6-dihydropyrimidin-1(2*H*)-yl)phenoxy)-1-(cyclobutylamino)butylidene)-1,3-dimethylpyrimidine-2,4,6(1*H*,3*H*,5*H*)-trione (**BA-8**): white solid, yield 73%, m.p. 249–251 °C; ^1^H NMR (500 MHz, CDCl_3_) *δ* 12.73 (d, *J* = 5.9 Hz, 1H), 7.35 (d, *J* = 8.8 Hz, 1H), 6.80 (d, *J* = 6.3 Hz, 1H), 6.38 (s, 1H), 4.47 (dd, *J* = 15.7, 7.7 Hz, 1H), 4.12 (t, *J* = 5.4 Hz, 2H), 3.57 (s, 3H), 3.38–3.24 (m, 8H), 2.58–2.45 (m, 2H), 2.25–2.06 (m, 4H), 1.94–1.76 (m, 2H); ^13^C NMR (125 MHz, CDCl_3_) *δ* 174.83 (s), 166.68 (s), 162.56 (s), 159.92 (s), 151.42 (d, *J* = 247.8 Hz), 151.33 (s), 151.04 (d, *J* = 2.6 Hz), 150.69 (s), 141.79 (q, *J* = 34.5 Hz), 124.38 (d, *J* = 9.3 Hz), 120.72 (d, *J* = 14.9 Hz), 119.38 (q, *J* = 275.4 Hz), 118.50 (d, *J* = 24.2 Hz), 113.42 (s), 103.10 (q, *J* = 5.6 Hz), 89.55 (s), 68.89 (s), 47.92 (s), 32.73 (q, *J* = 3.5 Hz), 31.37 (s), 31.35 (s), 27.92 (s), 27.66 (s), 27.30 (s), 27.26 (s), 15.36 (s); HRMS, *m*/*z* calcd. for C_26_H_27_ClF_4_N_5_O_6_^+^ [M + H]^+^ 616.15805, found 616.15747.

5-(4-(2-Chloro-4-fluoro-5-(3-methyl-2,6-dioxo-4-(trifluoromethyl)-3,6-dihydropyrimidin-1(2*H*)-yl)phenoxy)-1-(cyclopentylamino)butylidene)-1,3-dimethylpyrimidine-2,4,6(1*H*,3*H*,5*H*)-trione (**BA-9**): white solid, yield 29%, m.p. 264–266 °C; ^1^H NMR (500 MHz, CDCl_3_) *δ* 12.76 (d, *J* = 7.4 Hz, 1H), 7.33 (d, *J* = 8.8 Hz, 1H), 6.80 (d, *J* = 6.3 Hz, 1H), 6.38 (s, 1H), 4.37 (dd, *J* = 13.6, 6.7 Hz, 1H), 4.13 (t, *J* = 5.4 Hz, 2H), 3.57 (s, 3H), 3.38 (dd, *J* = 9.4, 6.1 Hz, 2H), 3.32 (s, 3H), 3.30 (s, 3H), 2.23–2.08 (m, 4H), 1.92–1.79 (m, 2H), 1.76–1.62 (m, 4H); ^13^C NMR (125 MHz, CDCl_3_) *δ* 174.95 (s), 166.74 (s), 162.55 (s), 159.92 (s), 151.41 (d, *J* = 248.1 Hz), 151.37 (s), 151.00 (d, *J* = 2.5 Hz), 150.68 (s), 141.78 (q, *J* = 34.3 Hz), 124.35 (d, *J* = 9.5 Hz), 120.73 (d, *J* = 14.8 Hz), 119.37 (q, *J* = 275.5 Hz), 118.45 (d, *J* = 24.2 Hz), 113.43 (s), 103.10 (q, *J* = 5.5 Hz), 89.39 (s), 68.86 (s), 54.89 (s), 34.19 (s), 32.73 (q, *J* = 3.1 Hz), 27.92 (s), 27.65 (s), 27.44 (s), 27.10 (s), 23.98 (s); HRMS, *m*/*z* calcd. for C_27_H_29_ClF_4_N_5_O_6_^+^ [M + H]^+^ 630.17370, found 630.17291.

5-(4-(2-Chloro-4-fluoro-5-(3-methyl-2,6-dioxo-4-(trifluoromethyl)-3,6-dihydropyrimidin-1(2*H*)-yl)phenoxy)-1-(cyclohexylamino)butylidene)-1,3-dimethylpyrimidine-2,4,6(1*H*,3*H*,5*H*)-trione (**BA-10**): white solid, yield 23%, m.p. 264–267 °C; ^1^H NMR (500 MHz, CDCl_3_) *δ* 12.75 (d, *J* = 8.0 Hz, 1H), 7.33 (d, *J* = 8.8 Hz, 1H), 6.80 (d, *J* = 6.3 Hz, 1H), 6.37 (d, *J* = 4.3 Hz, 1H), 4.12 (t, *J* = 5.4 Hz, 2H), 3.93–3.83 (m, 1H), 3.57 (s, 3H), 3.38–3.28 (m, 8H), 2.16 (dt, *J* = 12.2, 6.3 Hz, 2H), 1.94 (d, *J* = 9.6 Hz, 2H), 1.79 (dd, *J* = 9.1, 4.0 Hz, 2H), 1.45 (m, 6H); ^13^C NMR (125 MHz, CDCl_3_) *δ* 174.54 (s), 166.80 (s), 162.57 (s), 159.92 (s), 151.43 (d, *J* = 248.1 Hz), 151.39 (s), 150.99 (d, *J* = 2.5 Hz), 150.68 (s), 141.79 (q, *J* = 34.4 Hz), 124.44 (d, *J* = 9.3 Hz), 120.73 (d, *J* = 14.8 Hz), 119.37 (q, *J* = 275.5 Hz), 118.45 (d, *J* = 24.1 Hz), 113.44 (s), 103.10 (q, *J* = 5.3 Hz), 89.31 (s), 68.82 (s), 52.14 (s), 33.58 (s), 32.74 (q, *J* = 3.4 Hz), 27.93 (s), 27.90 (s), 27.66 (s), 26.69 (s), 25.04 (s), 24.10 (s); HRMS, *m*/*z* calcd. for C_28_H_31_ClF_4_N_5_O_6_^+^ [M + H]^+^ 644.18935, found 644.18866.

5-(4-(2-Chloro-4-fluoro-5-(3-methyl-2,6-dioxo-4-(trifluoromethyl)-3,6-dihydropyrimidin-1(2*H*)-yl)phenoxy)-1-(isopropylamino)butylidene)-1,3-dimethylpyrimidine-2,4,6(1*H*,3*H*,5*H*)-trione (**BA-11**): white solid, yield 48%, m.p. 264–267 °C; ^1^H NMR (500 MHz, CDCl_3_) *δ* 12.63 (d, *J* = 8.0 Hz, 1H), 7.33 (d, *J* = 8.8 Hz, 1H), 6.81 (d, *J* = 6.3 Hz, 1H), 6.38 (s, 1H), 4.34–4.23 (m, 1H), 4.12 (t, *J* = 5.4 Hz, 2H), 3.57 (s, 3H), 3.43–3.28 (m, 8H), 2.22–2.11 (m, 2H), 1.37 (s, 3H), 1.35 (s, 3H); ^13^C NMR (125 MHz, CDCl_3_) *δ* 174.59 (s), 166.76 (s), 162.58 (s), 159.92 (s), 151.44 (d, *J* = 248.1 Hz), 151.36 (s), 150.99 (d, *J* = 2.6 Hz), 150.68 (s), 141.78 (q, *J* = 34.6 Hz), 124.39 (d, *J* = 9.5 Hz), 120.73 (d, *J* = 14.8 Hz), 119.37 (q, *J* = 275.5 Hz), 118.46 (d, *J* = 24.1 Hz), 113.48 (s), 103.09 (q, *J* = 5.5 Hz), 89.26 (s), 68.84 (s), 45.57 (s), 32.73 (q, *J* = 3.5 Hz), 27.93 (s), 27.76 (s), 27.63 (s), 26.62 (s), 23.57 (s); HRMS, *m*/*z* calcd. for C_25_H_27_ClF_4_N_5_O_6_^+^ [M + H]^+^ 604.15805, found 604.15765.

5-(4-(2-Chloro-4-fluoro-5-(3-methyl-2,6-dioxo-4-(trifluoromethyl)-3,6-dihydropyrimidin-1(2*H*)-yl)phenoxy)-1-(isobutylamino)butylidene)-1,3-dimethylpyrimidine-2,4,6(1*H*,3*H*,5*H*)-trione (**BA-12**): white solid, yield 76%, m.p. 241–244 °C; ^1^H NMR (500 MHz, CDCl_3_) *δ* 12.71 (s, 1H), 7.33 (d, *J* = 8.8 Hz, 1H), 6.80 (d, *J* = 6.3 Hz, 1H), 6.38 (s, 1H), 4.12 (t, *J* = 5.3 Hz, 2H), 3.57 (s, 3H), 3.44–3.40 (m, 2H), 3.39–3.28 (m, 8H), 2.15 (m, 2H), 2.00 (m, 1H), 1.06 (s, 3H), 1.05 (s, 3H); ^13^C NMR (125 MHz, CDCl_3_) δ 176.16 (s), 166.77 (s), 162.57 (s), 159.89 (s), 151.41 (d, *J* = 248.0 Hz), 151.34 (s), 150.97 (d, *J* = 2.6 Hz), 150.66 (s), 141.75 (q, *J* = 34.5 Hz), 124.42 (d, *J* = 9.5 Hz), 120.70 (d, *J* = 14.9 Hz), 119.37 (q, *J* = 275.6 Hz), 118.42 (d, *J* = 24.4 Hz), 113.47 (s), 103.07 (q, *J* = 5.4 Hz), 89.64 (s), 68.80 (s), 50.85 (s), 32.71 (q, *J* = 3.5 Hz), 28.71 (s), 27.90 (s), 27.65 (s), 26.99 (s), 26.79 (s), 20.12 (s); HRMS, *m*/*z* calcd. for C_26_H_29_ClF_4_N_5_O_6_^+^ [M + H]^+^ 618.17370, found 618.17346.

5-(1-(sec-Butylamino)-4-(2-chloro-4-fluoro-5-(3-methyl-2,6-dioxo-4-(trifluoromethyl)-3,6-dihydropyrimidin-1(2*H*)-yl)phenoxy)butylidene)-1,3-dimethylpyrimidine-2,4,6(1*H*,3*H*,5*H*)-trione (**BA-13**): white solid, yield 36%, m.p. 269–271 °C; ^1^H NMR (500 MHz, CDCl_3_) *δ* 12.63 (d, *J* = 8.4 Hz, 1H), 7.33 (d, *J* = 8.8 Hz, 1H), 6.80 (d, *J* = 6.3 Hz, 1H), 6.38 (s, 1H), 4.18–4.06 (m, 3H), 3.57 (s, 3H), 3.45–3.28 (m, 8H), 2.26–2.07 (m, 2H), 1.74–1.62 (m, 2H), 1.32 (d, *J* = 6.4 Hz, 3H), 0.99 (t, *J* = 7.4 Hz, 3H); ^13^C NMR (125 MHz, CDCl_3_) *δ* 175.02 (s), 166.80 (s), 162.59 (s), 159.92 (s), 151.43 (d, *J* = 248.1 Hz), 151.38 (s), 151.01 (d, *J* = 2.5 Hz), 150.68 (s), 141.78 (q, *J* = 34.6 Hz), 124.39 (d, *J* = 9.3 Hz), 120.73 (d, *J* = 14.8 Hz), 119.37 (q, *J* = 275.3 Hz), 118.46 (d, *J* = 24.1 Hz), 113.44 (s), 103.10 (q, *J* = 5.1 Hz), 89.29 (s), 68.84 (s), 51.02 (s), 32.73 (q, *J* = 3.4 Hz), 30.27 (s), 27.92 (s), 27.70 (s), 27.64 (s), 26.64 (s), 21.49 (s), 10.35 (s); HRMS, *m*/*z* calcd. for C_26_H_29_ClF_4_N_5_O_6_^+^ [M + H]^+^ 618.17370, found 618.17316.

5-(4-(2-Chloro-4-fluoro-5-(3-methyl-2,6-dioxo-4-(trifluoromethyl)-3,6-dihydropyrimidin-1(2*H*)-yl)phenoxy)-1-((2-hydroxyethyl)amino)butylidene)-1,3-dimethylpyrimidine-2,4,6(1*H*,3*H*,5*H*)-trione (**BA-14**): white solid, yield 82%, m.p. 214–216 °C; ^1^H NMR (500 MHz, CDCl_3_) *δ* 12.81 (s, 1H), 7.33 (d, *J* = 8.8 Hz, 1H), 6.80 (d, *J* = 6.3 Hz, 1H), 6.38 (s, 1H), 4.15 (t, *J* = 5.4 Hz, 2H), 3.88 (t, *J* = 5.1 Hz, 2H), 3.71 (dd, *J* = 10.4, 5.3 Hz, 2H), 3.57 (s, 3H), 3.41–3.34 (m, 2H), 3.31 (d, *J* = 5.5 Hz, 6H), 2.17 (m, 3H); ^13^C NMR (125 MHz, CDCl_3_) *δ* 176.53 (s), 166.65 (s), 162.52 (s), 160.02 (s), 151.43 (d, *J* = 248.1 Hz), 151.35 (s), 150.83 (d, *J* = 2.5 Hz), 150.72 (s), 141.87 (q, *J* = 34.7 Hz), 124.49 (d, *J* = 9.5 Hz), 120.67 (d, *J* = 14.8 Hz), 119.34 (q, *J* = 275.4 Hz), 118.51 (d, *J* = 24.1 Hz), 113.65 (s), 103.10 (q, *J* = 5.3 Hz), 89.92 (s), 68.69 (s), 60.66 (s), 45.45 (s), 32.78 (q, *J* = 3.3 Hz), 27.95 (s), 27.72 (s), 26.83 (s), 26.79 (s); HRMS, *m*/*z* calcd. for C_24_H_25_ClF_4_N_5_O_7_^+^ [M + H]^+^ 606.13732, found 606.13708.

5-(4-(2-Chloro-4-fluoro-5-(3-methyl-2,6-dioxo-4-(trifluoromethyl)-3,6-dihydropyrimidin-1(2*H*)-yl)phenoxy)-1-((3-hydroxypropyl)amino)butylidene)-1,3-dimethylpyrimidine-2,4,6(1*H*,3*H*,5*H*)-trione (**BA-15**): white solid, yield 80%, m.p. 221–224 °C; ^1^H NMR (500 MHz, CDCl_3_) *δ* 12.66 (s, 1H), 7.33 (d, *J* = 8.8 Hz, 1H), 6.79 (d, J = 6.3 Hz, 1H), 6.38 (s, 1H), 4.14 (t, *J* = 5.4 Hz, 2H), 3.74 (t, *J* = 5.8 Hz, 2H), 3.70 (dd, *J* = 12.3, 6.4 Hz, 2H), 3.57 (s, 3H), 3.42–3.34 (m, 2H), 3.31 (s, 6H), 2.24–2.14 (m, 2H), 1.93 (p, *J* = 6.3 Hz, 2H), 1.74 (s, 1H); ^13^C NMR (125 MHz, CDCl_3_) *δ* 176.40 (s), 166.74 (s), 162.54 (s), 159.92 (s), 151.46 (d, *J* = 248.0 Hz), 151.30 (s), 151.01 (d, *J* = 2.3 Hz), 150.69 (s), 141.77 (q, *J* = 34.5 Hz), 135.64 (s), 129.22 (s), 128.27 (s), 127.30 (s), 124.51 (d, *J* = 9.3 Hz), 120.70 (d, *J* = 14.8 Hz), 119.38 (q, *J* = 275.2 Hz), 118.47 (d, *J* = 24.1 Hz), 113.56 (s), 103.09 (q, *J* = 5.2 Hz), 90.16 (s), 68.95 (s), 47.29 (s), 32.73 (q, *J* = 3.5 Hz), 27.97 (s), 27.71 (s), 27.16 (s), 26.97 (s); HRMS, *m*/*z* calcd. for C_25_H_27_ClF_4_N_5_O_7_^+^ [M + H]^+^ 620.15297, found 620.15247.

5-(4-(2-Chloro-4-fluoro-5-(3-methyl-2,6-dioxo-4-(trifluoromethyl)-3,6-dihydropyrimidin-1(2*H*)-yl)phenoxy)-1-((2-hydroxypropyl)amino)butylidene)-1,3-dimethylpyrimidine-2,4,6(1*H*,3*H*,5*H*)-trione (**BA-16**): white solid, yield 82%, m.p. 234–237 °C; ^1^H NMR (500 MHz, CDCl_3_) *δ* 12.83 (s, 1H), 7.33 (d, *J* = 8.8 Hz, 1H), 6.80 (d, *J* = 6.3 Hz, 1H), 6.37 (d, *J* = 1.7 Hz, 1H), 4.14 (t, *J* = 5.4 Hz, 2H), 4.12–4.02 (m, 1H), 3.69–3.61 (m, 1H), 3.57 (s, 3H), 3.49–3.25 (m, 9H), 2.15 (dd, *J* = 12.2, 5.0 Hz, 3H), 1.27 (d, *J* = 6.2 Hz, 3H); ^13^C NMR (125 MHz, CDCl_3_) *δ* 176.31 (s), 166.65 (s), 162.56 (s), 160.03 (s), 159.97 (s), 151.42 (d, *J* = 248.2 Hz), 151.36 (s), 150.82 (d, *J* = 2.6 Hz), 150.74 (s), 150.69 (s), 141.86 (dd, *J* = 34.3, 3.7 Hz), 124.45 (d, *J* = 9.3 Hz), 120.68 (d, *J* = 14.8 Hz), 119.34 (q, *J* = 275.6 Hz), 118.49 (d, *J* = 24.3 Hz), 113.59 (s), 103.10 (p, *J* = 5.4 Hz), 89.89 (s), 68.57 (s), 66.07 (s), 50.28 (s), 32.77 (q, *J* = 3.5 Hz), 27.95 (s), 27.73 (s), 26.81 (s), 21.10 (s); HRMS, *m*/*z* calcd. for C_25_H_27_ClF_4_N_5_O_7_^+^ [M + H]^+^ 620.15297, found 620.15240.

5-(4-(2-Chloro-4-fluoro-5-(3-methyl-2,6-dioxo-4-(trifluoromethyl)-3,6-dihydropyrimidin-1(2*H*)-yl)phenoxy)-1-((2-methoxyethyl)amino)butylidene)-1,3-dimethylpyrimidine-2,4,6(1*H*,3*H*,5*H*)-trione (**BA-17**): white solid, yield 91%, m.p. 207–209 °C; ^1^H NMR (500 MHz, CDCl_3_) *δ* 12.73 (s, 1H), 7.32 (d, *J* = 8.8 Hz, 1H), 6.80 (d, *J* = 6.3 Hz, 1H), 6.38 (s, 1H), 4.12 (t, *J* = 5.4 Hz, 2H), 3.80 (dd, *J* = 10.4, 5.2 Hz, 2H), 3.64 (t, *J* = 5.1 Hz, 2H), 3.57 (s, 3H), 3.44 (s, 3H), 3.37 (dd, *J* = 9.3, 6.3 Hz, 2H), 3.34 (s, 3H), 3.30 (s, 3H), 2.16 (m, 2H); ^13^C NMR (125 MHz, CDCl_3_) *δ* 176.28 (s), 166.62 (s), 162.61 (s), 159.92 (s), 151.44 (d, *J* = 248.0 Hz), 151.38 (s), 150.99 (d, *J* = 2.6 Hz), 150.68 (s), 141.78 (q, *J* = 34.5 Hz), 124.42 (d, *J* = 9.3 Hz), 120.71 (d, J = 14.9 Hz), 119.37 (q, *J* = 275.4 Hz), 118.46 (d, *J* = 24.1 Hz), 113.53 (s), 103.09 (q, *J* = 5.3 Hz), 89.94 (s), 70.25 (s), 68.85 (s), 59.22 (s), 43.38 (s), 32.73 (d, *J* = 3.4 Hz), 27.93 (s), 27.70 (s), 26.97 (s), 26.86 (s); HRMS, *m*/*z* calcd. for C_25_H_27_ClF_4_N_5_O_7_^+^ [M + H]^+^ 620.15297, found 620.15228.

5-(4-(2-Chloro-4-fluoro-5-(3-methyl-2,6-dioxo-4-(trifluoromethyl)-3,6-dihydropyrimidin-1(2*H*)-yl)phenoxy)-1-((3-methoxypropyl)amino)butylidene)-1,3-dimethylpyrimidine-2,4,6(1*H*,3*H*,5*H*)-trione (**BA-18**): white solid, yield 79%, m.p. 197–200 °C; ^1^H NMR (500 MHz, CDCl_3_) *δ* 12.65 (s, 1H), 7.32 (d, *J* = 8.8 Hz, 1H), 6.80 (d, *J* = 6.3 Hz, 1H), 6.38 (s, 1H), 4.13 (t, *J* = 5.5 Hz, 2H), 3.72 (dd, *J* = 12.4, 6.6 Hz, 2H), 3.57 (s, 3H), 3.50 (t, *J* = 5.8 Hz, 2H), 3.39–3.34 (m, 5H), 3.33 (s, 3H), 3.30 (s, 3H), 2.16 (m, 2H), 2.02–1.94 (m, 2H); ^13^C NMR (125 MHz, CDCl_3_) *δ* 176.21 (s), 166.66 (s), 162.59 (s), 159.91 (s), 151.41 (d, *J* = 247.9 Hz), 151.37 (s), 151.05 (d, *J* = 2.3 Hz), 150.68 (s), 141.76 (q, *J* = 34.6 Hz), 124.53 (d, *J* = 9.3 Hz), 120.65 (d, *J* = 14.8 Hz), 119.38 (q, *J* = 275.4 Hz), 118.43 (d, *J* = 24.1 Hz), 113.50 (s), 103.09 (q, *J* = 5.4 Hz), 89.73 (s), 69.24 (s), 68.91 (s), 58.80 (s), 40.80 (s), 32.72 (q, *J* = 3.5 Hz), 29.50 (s), 27.91 (s), 27.63 (s), 27.05 (s), 26.65 (s); HRMS, *m*/*z* calcd. for C_26_H_29_ClF_4_N_5_O_7_^+^ [M + H]^+^ 634.16862, found 634.16809.

5-(4-(2-Chloro-4-fluoro-5-(3-methyl-2,6-dioxo-4-(trifluoromethyl)-3,6-dihydropyrimidin-1(2*H*)-yl)phenoxy)-1-(phenylamino)butylidene)-1,3-dimethylpyrimidine-2,4,6(1*H*,3*H*,5*H*)-trione (**BA-19**): white solid, yield 16%, m.p. 198–200 °C; ^1^H NMR (500 MHz, CDCl_3_) *δ* 14.14 (s, 1H), 7.41 (t, *J* = 7.5 Hz, 2H), 7.35 (t, *J* = 7.4 Hz, 1H), 7.22 (d, *J* = 8.9 Hz, 1H), 7.17 (d, *J* = 7.6 Hz, 2H), 6.68 (d, *J* = 6.3 Hz, 1H), 6.36 (s, 1H), 3.99 (t, *J* = 5.8 Hz, 2H), 3.56 (s, 3H), 3.37 (s, 3H), 3.33 (s, 3H), 3.27–3.20 (m, 2H), 2.08 (m, 2H); ^13^C NMR (125 MHz, CDCl_3_) *δ* 176.45 (s), 167.01 (s), 162.31 (s), 159.90 (s), 151.25 (s), 151.23 (d, *J* = 247.3 Hz), 150.85 (d, *J* = 2.5 Hz), 150.67 (s), 141.73 (q, *J* = 34.1 Hz), 136.00 (s), 129.75 (s), 128.37 (s), 126.28 (s), 124.62 (d, *J* = 9.3 Hz), 120.39 (d, *J* = 14.8 Hz), 119.38 (q, *J* = 275.1 Hz), 118.30 (d, *J* = 24.2 Hz), 113.24 (s), 103.07 (q, *J* = 5.2 Hz), 90.47 (s), 68.85 (s), 32.71 (q, *J* = 3.4 Hz), 28.04 (s), 27.96 (s), 27.81 (s), 27.73 (s); HRMS, *m*/*z* calcd. for C_28_H_25_ClF_4_N_5_O_6_^+^ [M + H]^+^ 638.14240, found 638.14160.

5-(1-(Benzylamino)-4-(2-chloro-4-fluoro-5-(3-methyl-2,6-dioxo-4-(trifluoromethyl)-3,6-dihydropyrimidin-1(2*H*)-yl)phenoxy)butylidene)-1,3-dimethylpyrimidine-2,4,6(1*H*,3*H*,5*H*)-trione (**BA-20**): white solid, yield 36%, m.p. 234–236 °C; ^1^H NMR (500 MHz, CDCl_3_) *δ* 12.97 (s, 1H), 7.40 (t, *J* = 7.3 Hz, 2H), 7.36–7.29 (m, 4H), 6.80 (d, *J* = 6.3 Hz, 1H), 6.38 (s, 1H), 4.81 (d, *J* = 5.8 Hz, 2H), 4.13 (t, *J* = 5.4 Hz, 2H), 3.57 (s, 3H), 3.42 (dd, *J* = 9.3, 6.0 Hz, 2H), 3.32 (s, 3H), 3.31 (s, 3H), 2.18 (m, 2H); ^13^C NMR (125 MHz, CDCl_3_) δ 176.40 (s), 166.75 (s), 162.54 (s), 159.88 (s), 151.50 (d, *J* = 248.0 Hz), 151.30 (s), 151.02 (d, *J* = 2.6 Hz), 150.68 (s), 141.77 (q, *J* = 34.5 Hz), 135.64 (s), 129.21 (s), 128.26 (s), 127.29 (s), 124.56 (d, *J* = 9.5 Hz), 120.72 (d, *J* = 14.8 Hz), 119.39 (q, *J* = 275.6 Hz), 118.45 (d, *J* = 24.1 Hz), 113.68 (s), 103.09 (q, *J* = 5.2 Hz), 90.17 (s), 69.02 (s), 47.30 (s), 32.70 (q, *J* = 3.5 Hz), 27.95 (s), 27.68 (s), 27.18 (s), 26.95 (s); HRMS, *m*/*z* calcd. for C_29_H_27_ClF_4_N_5_O_6_^+^ [M + H]^+^ 652.15805, found 652.15729.

### 3.2. Evaluation of Herbicidal Activity

According to the methods previously reported [11], the post-emergence herbicidal activities of target compounds **BA-1**~**BA-20** against four representative plants (i.e., *B. campestris*, *A. tricolor*, *E. crus-galli*, *D. sanguinalis*) were evaluated in the greenhouse. Sixteen plants, including *B. campestris*, *A. tricolor*, *L. indica*, *P. oleracea*, *T. mongolicum*, *C. album*, *B. tripartita*, *I. nil*, *E. crus-galli*, *D. sanguinalis*, *S. viridis*, *E. indica*, *S. alterniflora*, *P. alopecuroides*, *C. virgata*, and *E. dahuricus*, were chosen to investigate the herbicidal spectrum of target compound **BA-1**. Briefly, 15~20 weed seeds were uniformly sown in an 8 cm × 7 cm × 7 cm plastic pot filled with a 2:1 (*w*/*w*) mixture of sandy soil and nutrient matrix. The seedlings were cultivated in a greenhouse maintained at 28 ± 2 °C under a 16 h light–8 h dark photoperiod. The tested compounds were initially dissolved in 100% DMF and subsequently diluted with Tween-80 (concentration: 100 g/L). The resulting solutions were further diluted with distilled water (pH 7.0) to the desired concentrations prior to application. When the second true leaves had fully expanded, the seedlings were thinned to 10 plants per pot and sprayed with the evaluated compounds using a laboratory sprayer (model: 3WP-2000, Nanjing Research Institute for Agricultural Mechanization, Nanjing, Ministry of Agriculture, China), equipped with a flat-fan nozzle delivering a spray volume of 280 L ha^−1^ at 230 kPa. The initial herbicidal screening was conducted at a dosage of 150 g ha^−1^ for all target compounds. In the subsequent screening, the dosages for the selected compounds **BA-1**, **BA-2**, **BA-5**, **BA-18**, and **BA-20** were set at 75, 37.5, and 18.8 g ha^−1^, respectively. Flumiclorac-pentyl (FP) was employed as the positive control. A control group was treated with a mixture of distilled water, DMF, and Tween-80 in equal proportions. Each treatment was replicated three times, with a one-day interval between applications. After a 14-day observation period, the herbicidal activity of each compound was evaluated. The inhibition rate was calculated using the following formula: inhibition rate (%) = ((fresh weight of control − fresh weight of treatment)/fresh weight of control) × 100%.

### 3.3. Crop Safety

The assay method for crop safety followed procedures previously reported in the literature [11]. Four representative crop species, i.e., *T. aestivum*, *Z. mays*, *G. hirsutum*, and *G. max*, were selected for greenhouse-based crop safety evaluation. In detail, seeds of the selected crops were planted in plastic pots and grown under controlled greenhouse conditions. When the seedlings reached the four-leaf growth stage, they were sprayed with either of the test compounds, **BA-1** or FP, at a dosage of 37.5 g ha^−1^. The application process was conducted in accordance with the methodology described in Section 3.2. Each treatment was replicated three times, with a one-day interval between applications. Crop injury was evaluated 14 days post-treatment and expressed as the percentage of visible damage observed.

### 3.4. Molecular Simulation Analysis

The crystal structure of protoporphyrinogen IX oxidase (PPO, EC 1.3.3.4) from *Nicotiana tabacum* (PDB ID: 1SEZ, *Nt*PPO) was selected as the target protein for molecular docking studies. The active site was identified based on the coordinates of the co-crystallized ligand OMN using AutoDock Tools. The chemical structures of the ligands **BA-1** and FP were constructed using ChemBioDraw Ultra 14.0 and subjected to energy minimization via the MM2 force field method in ChemBio3D Ultra 14.0. The optimized structures were saved in mol2 format and subsequently converted into pdbqt format using AutoDock Tools for molecular docking analysis. Prior to docking, all water molecules and non-native ligands were removed from the protein structure. Kollman atomic charges and polar hydrogen atoms were added to ensure accurate electrostatic interactions. Molecular docking simulations between **BA-1** or FP and *Nt*PPO were performed using AutoDock Vina 4.2 [37,38,39]. The resulting protein-ligand complexes were visualized and analyzed using PyMOL v1.3 [40].

The molecular dynamics (MD) simulations were performed in accordance with established protocols, encompassing system preparation, force field parameter assignment, definition of initial conditions, integration of equations of motion, and data acquisition [41]. All simulations were executed using GROMACS 2023.3 on an Ubuntu 20.04.1 Linux operating system [42]. To assess the stability of the interactions over time, the complexes formed by **BA-1** or FP with *Nt*PPO were simulated separately. Each simulation system underwent a 50 ns MD simulation. Upon completion, root-mean-square deviation (RMSD) values were computed and analyzed. A lower RMSD suggests a smaller deviation from the reference structure throughout the simulation, indicating enhanced conformational stability of the complex.

### 3.5. Statistical Analysis

The values shown in each table are mean values ± SD of at least three repeated experiments. DPS 7.05 data processing system (DPS, Hangzhou, China) was used as a statistical software program.

## 4. Conclusions

In summary, twenty novel 5-(1-amino-4-phenoxybutylidene)barbituric acid derivatives incorporating an enamino diketone motif were synthesized in moderate yields and evaluated for their herbicidal activity. The bioassay results in the greenhouse demonstrated that some of the target compounds, such as **BA-1**, **BA-2**, **BA-5**, **BA-18**, and **BA-20**, exhibited promising herbicidal activity, and **BA-1** was confirmed as a potential herbicide lead compound due to its excellent herbicidal activity, broad herbicidal spectrum and good crop safety. The SAR study revealed that the steric effect and electronic effects of R group and the lipophilicity of target compound displayed an important effect on herbicidal activity. The molecular simulation analysis revealed that **BA-1** could bind well with *Nt*PPO, which indicated that it might be a novel ACCase inhibitor. The present work suggested that **BA-1** could represent a potential lead compound for further developing novel PPO-inhibiting herbicides. Further study on the structural optimization of **BA-1** is ongoing in our laboratory.

## Figures and Tables

**Table 1:** Effects (% inhibition) of the target compounds **BA-1**, **BA-2**, **BA-5**, **BA-18**, and **BA-20** on the loss of plant weight at different dosages in greenhouse testing ^a^.

Comp.	Dosage (g ha^−1^)	Inhibition Rate (%)				SIR
		*B. campestris*	*A. tricolor*	*E. crus-galli*	*D. sanguinalis*	
**BA-1**	75	100	100	57 ± 5	62 ± 2	87 ± 1
	37.5	94 ± 3	100	23 ± 4	16 ± 2	72 ± 3
	18.8	84 ± 2	100	18 ± 4	11 ± 4	64 ± 2
**BA-2**	75	92 ± 2	100	62 ± 8	44 ± 4	78 ± 2
	37.5	80 ± 5	100	51 ± 7	0 ± 0	66 ± 5
	18.8	58 ± 3	100	42 ± 5	0 ± 2	51 ± 4
**BA-5**	75	100	100	15 ± 1	35 ± 2	77 ± 1
	37.5	79 ± 3	100	11 ± 5	8 ± 6	59 ± 1
	18.8	40 ± 7	100	2 ± 6	0 ± 0	32 ± 6
**BA-18**	75	71 ± 8	100	42 ± 6	29 ± 10	63 ± 4
	37.5	70 ± 1	100	28 ± 9	6 ± 1	57 ± 3
	18.8	63 ± 1	100	21 ± 5	0 ± 4	50 ± 1
**BA-20**	75	74 ± 2	100	18 ± 13	2 ± 2	56 ± 3
	37.5	61 ± 3	72 ± 3	7 ± 6	0 ± 5	45 ± 3
	18.8	54 ± 4	56 ± 4	0 ± 3	0 ± 0	39 ± 1
FP	75	88 ± 4	100	52 ± 2	48 ± 3	77 ± 3
	37.5	53 ± 4	100	50 ± 3	42 ± 3	54 ± 4
	18.8	37 ± 6	90 ± 3	51 ± 6	13 ± 4	40 ± 2

^a^ Each value represents the mean ± SD of three experiments.

**Table 2:** Post-emergence crop selectivity of **BA-1** and FP at the dosage of 37.5 g ha^−1^ (Injury %) ^a^.

Comp.	Injury (%)			
	*T. aestivum*	*Z. mays*	*G. hirsutum*	*G. max*
**BA-1**	4 ± 2	2 ± 2	44 ± 2	47 ± 3
FP	5 ± 2	3 ± 1	33 ± 4	14 ± 1

^a^ Each value represents the mean ± SD of three experiments.

## Data Availability

The data are contained within the article; further inquiries can be directed to the corresponding authors.

## Supplementary Materials

The following supporting information can be downloaded at https://www.mdpi.com/article/10.3390/molecules30163445/s1, Figures S1–S60: ^1^HNMR, ^13^CNMR, HRMS of the target compounds **BA-1** to **BA-20**.
