# Supplementary material for: Design, Synthesis and Herbicidal Activity of 5-(1-Amino-4-phenoxybutylidene)barbituric Acid Derivatives Containing an Enamino Diketone Motif
Source: Molecules. 2025 Aug 21;30(16):3445. doi: 10.3390/molecules30163445 (PMC12388049; doi:10.3390/molecules30163445)
Supplement: Supplementary file 1 [file molecules-30-03445-s001.zip › molecules-3816173-supplementary.pdf]

Supporting Information

Design, Synthesis and Herbicidal Activity of 5-(1-Amino-4-phenoxybutylidene)barbituric Acid Derivatives Containing an Enamino Diketone Motif

Ke Chen [1], Shumin Wang [2], Shuyue Fu [2], Yuxiao Zhang [2], Wei Gao [2], Jin Liu [3], Rui Liu [1,*] and Kang Lei [2,*]

[1] Department of Biotechnology, The University of Suwon, Hwaseong 18323, Gyeonggi-Do, Republic of Korea
[2] School of Pharmaceutical Sciences and Food Engineering, Liaocheng University, Liaocheng 252059, China
[3] Shandong Academy of Innovation and Development, Jinan 250101, China
* Correspondence: liurcau@suwon.ac.kr (R.L.); leikang@lcu.edu.cn (K.L.)

1. General Information

Usually, the reagents and solvents, purchased form Energy Chemical or Tokyo Chemical Industry, were analytical grade and used without further purification. Column chromatography purification was carried out using silica gel column chromatography (silica gel 200 - 300 mesh) (Qingdao Makall Group Co., Ltd, Qingdao, China). The melting points were determined on a X-4 binocular microscope melting point apparatus (Gongyi Tech. Instrument Co., Henan, China) and without corrections. Nuclear magnetic resonance (NMR) spectra were recorded on an AV-500 spectrometer (Bruker Corp., Billerica, MA, USA) in $CDCl_3$ or DMSO-d_6 solution with tetramethylsilane (TMS) as the internal standard. High-resolution mass spectra (HRMS) was conducted using an Ionspec 7.0 T spectrometer (Varian, Palo Alto, CA) by the electrospray ionisation fourier transform ion cyclotron resonance technique.

2. The ^{1}H NMR, ^{13}C NMR, and HRMS Spectrum of Target Compounds

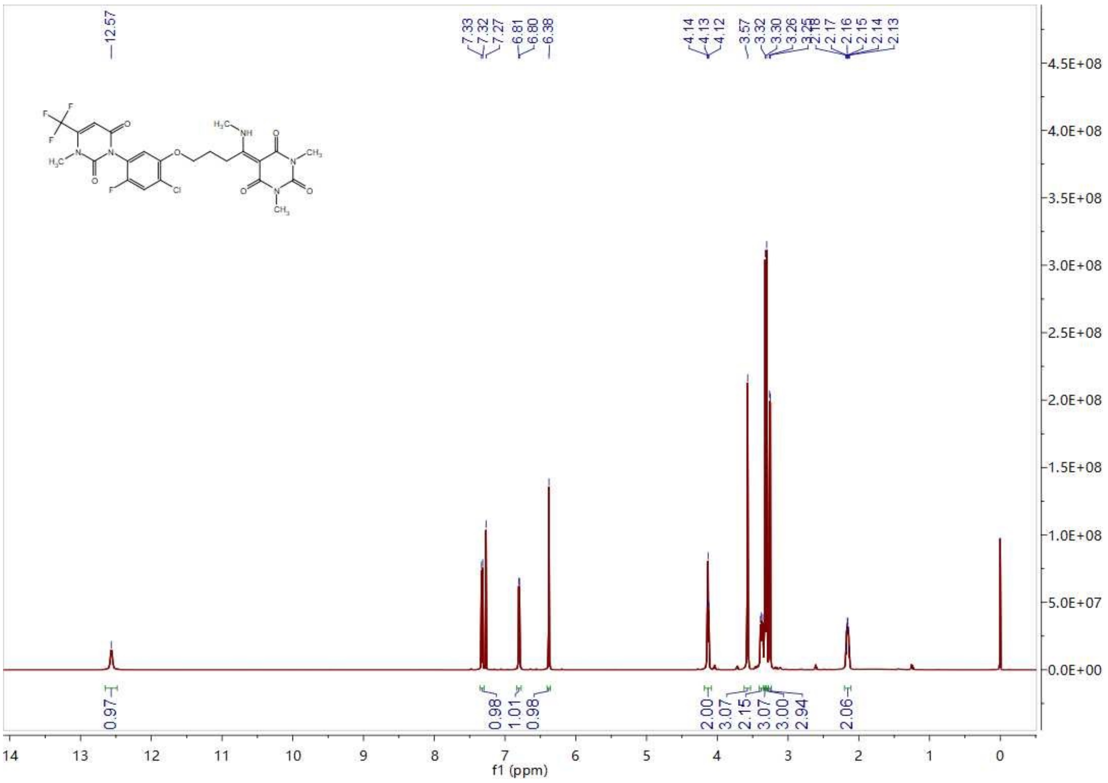

Figure S1 ^{1}HNMR of the target compound BA-1

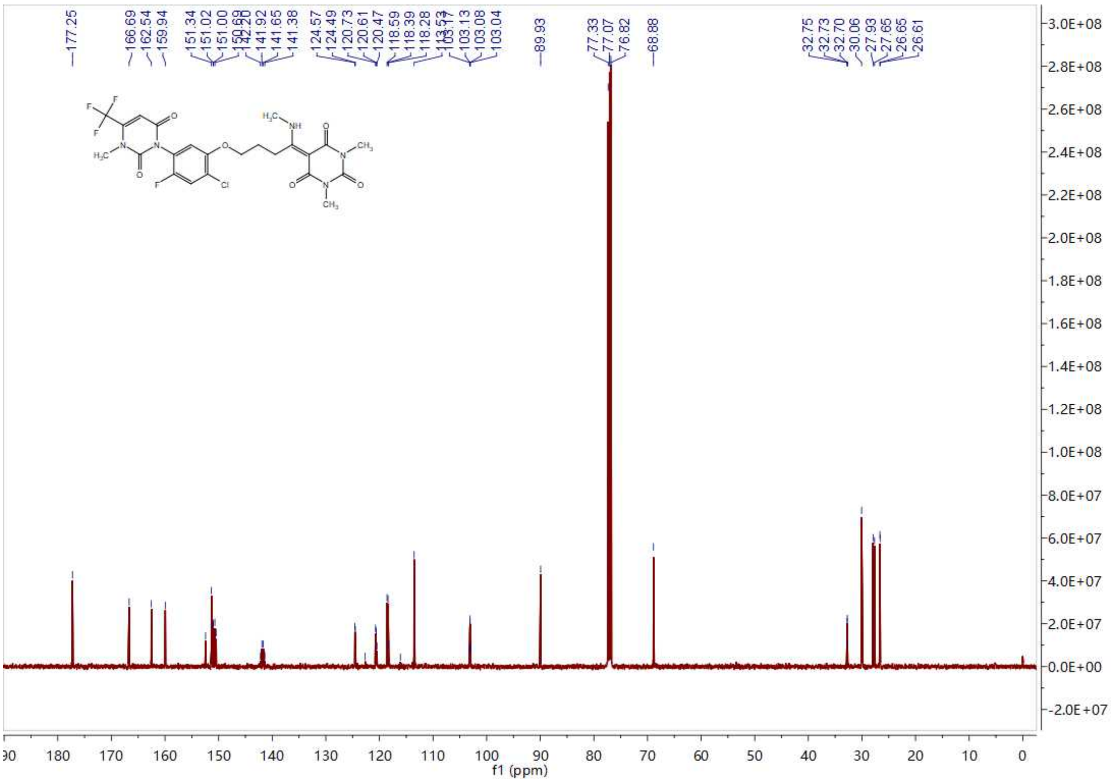

Figure S2 ^{13}CNMR of the target compound BA-1

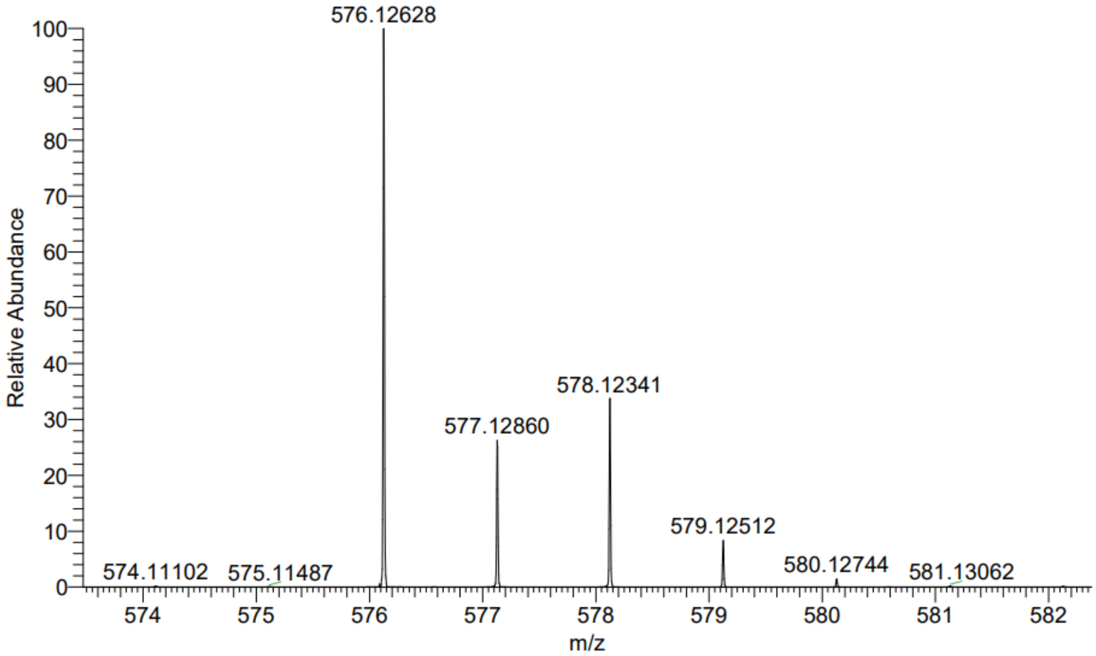

Figure S3 HRMS of the target compound BA-1

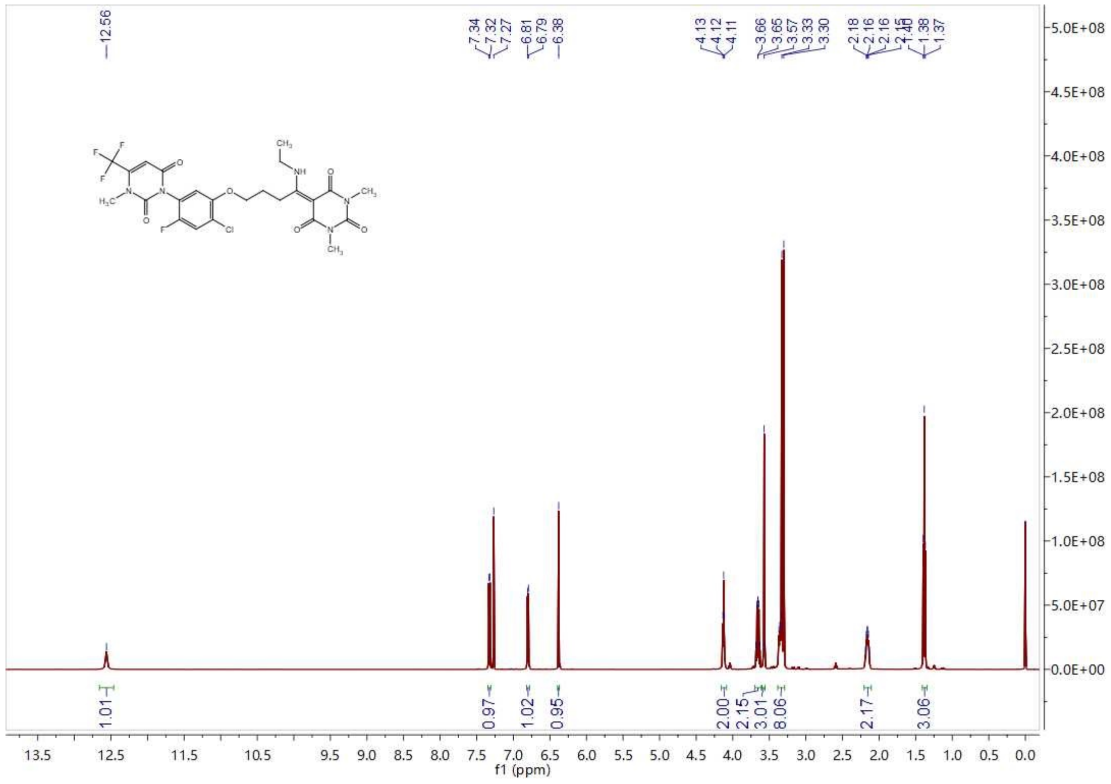

Figure S4 ^{1}HNMR of the target compound BA-2

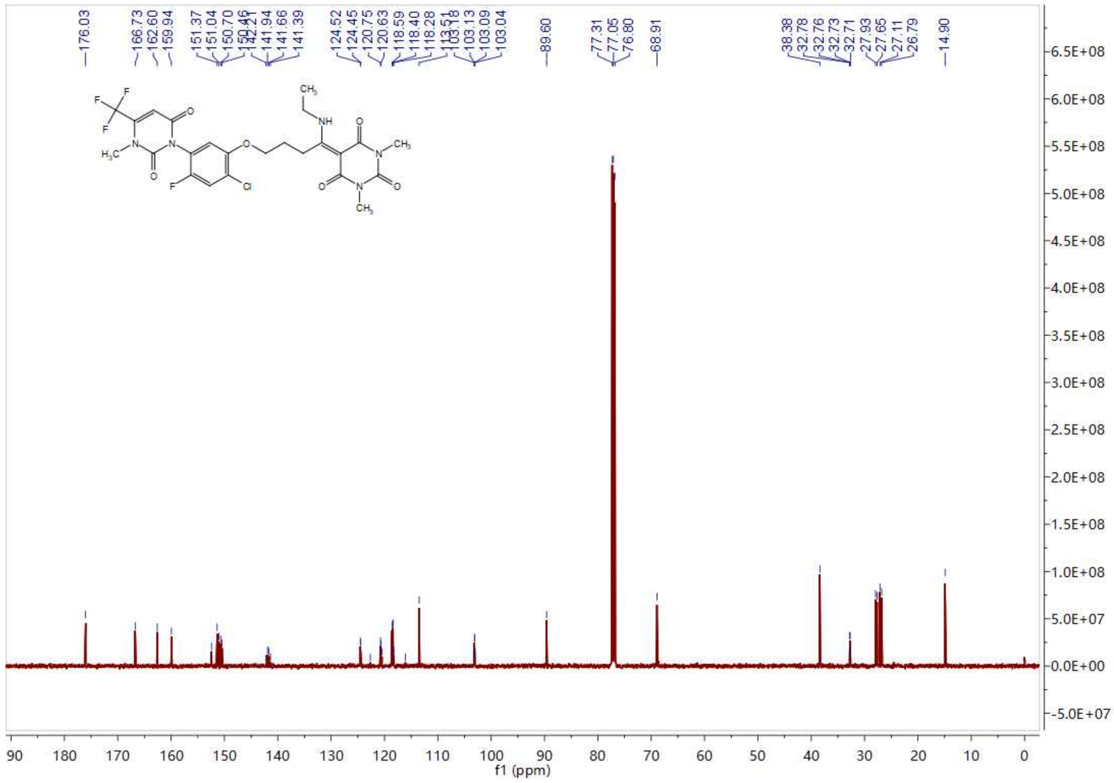

Figure S5 ^{13}CNMR of the target compound BA-2

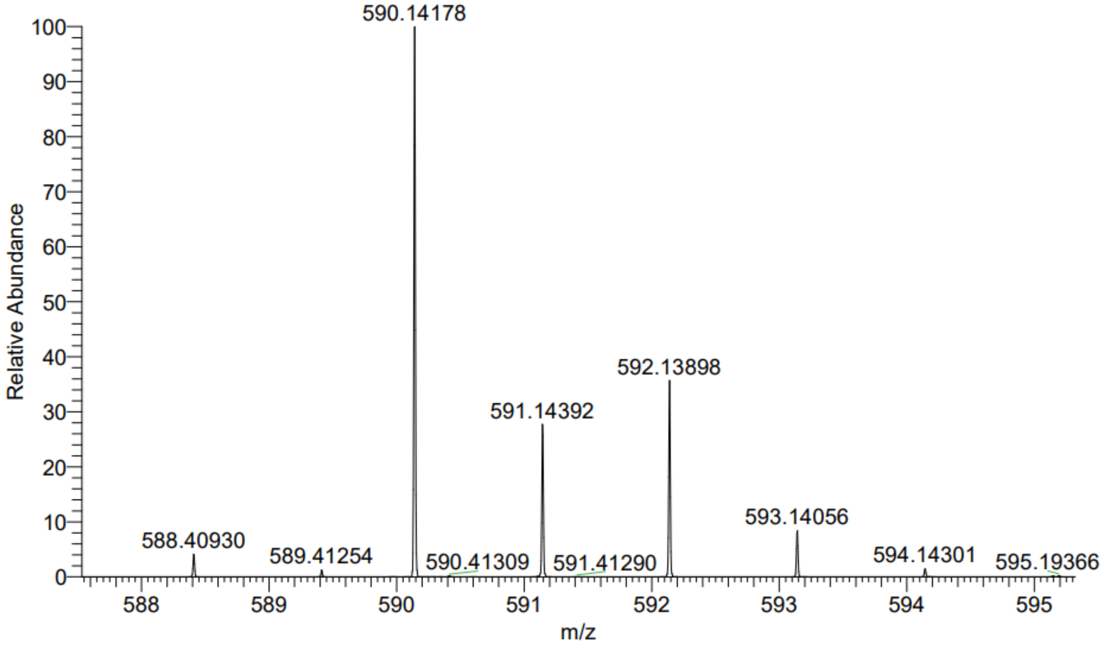

Figure S6 HRMS of the target compound BA-2

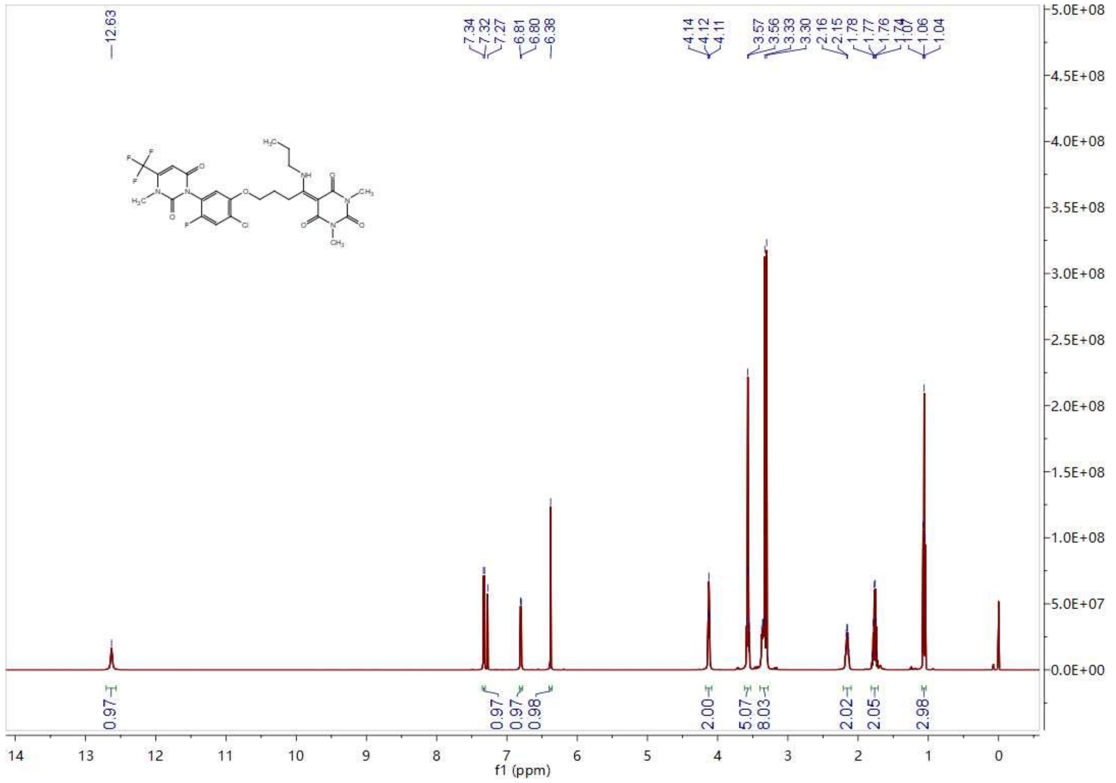

Figure S7 ¹HNMR of the target compound BA-3

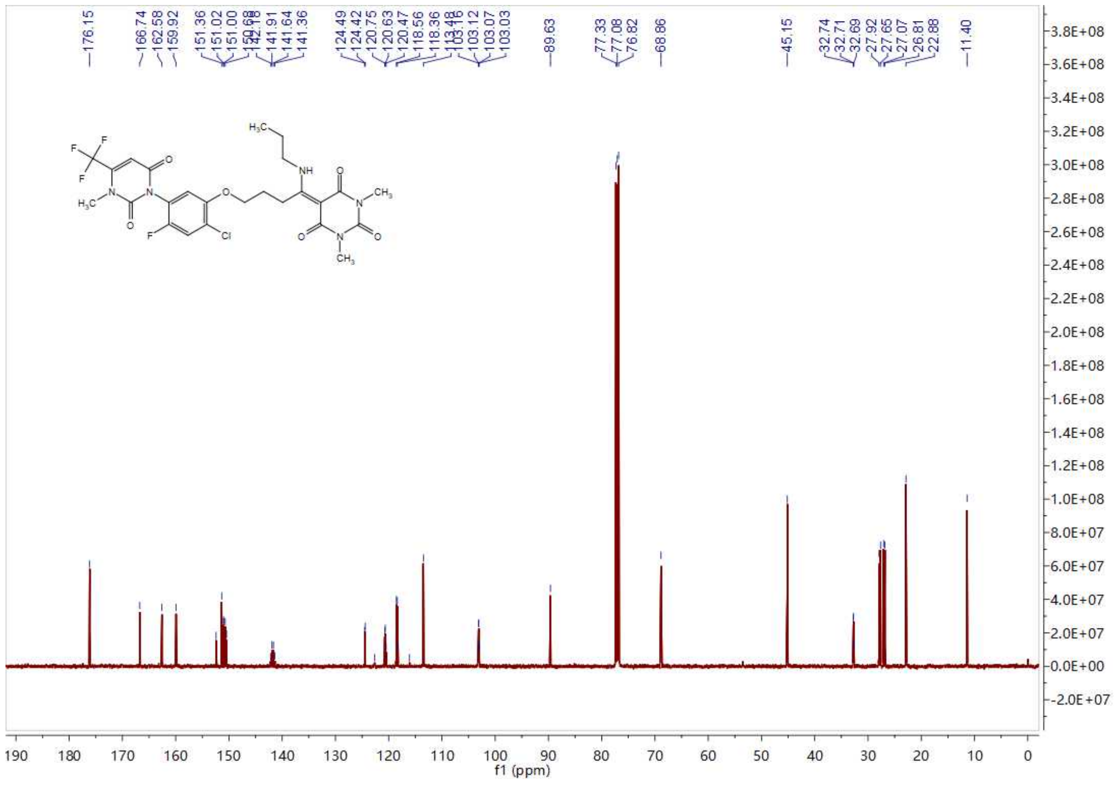

Figure S8 ¹³CNMR of the target compound BA-3

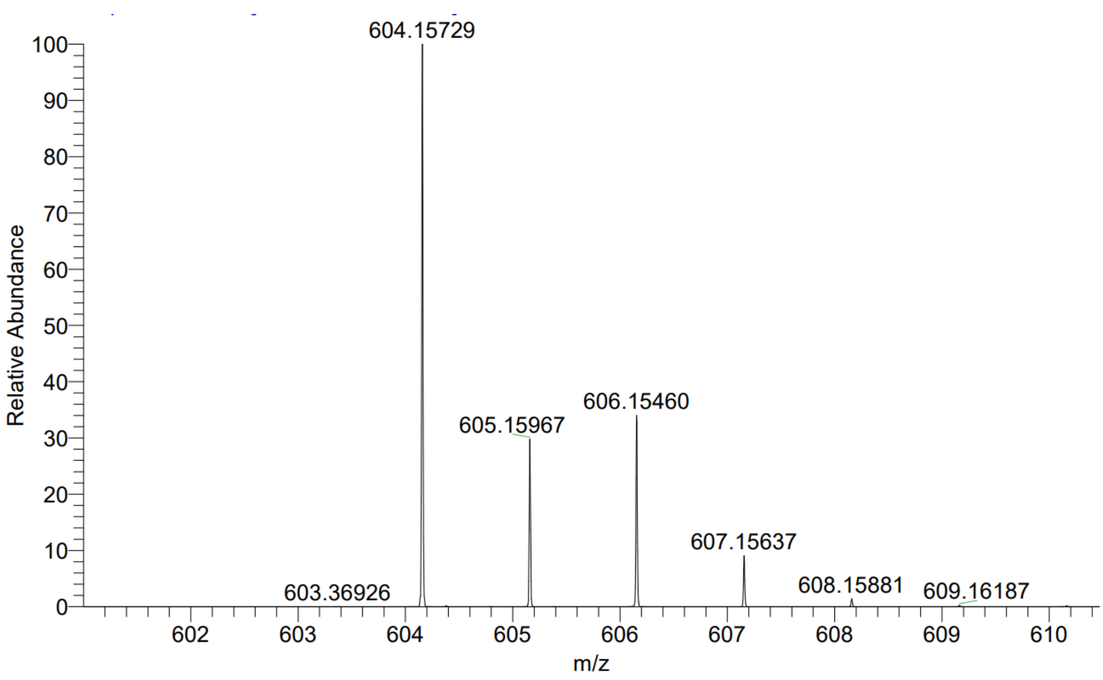

Figure S9 HRMS of the target compound BA-3

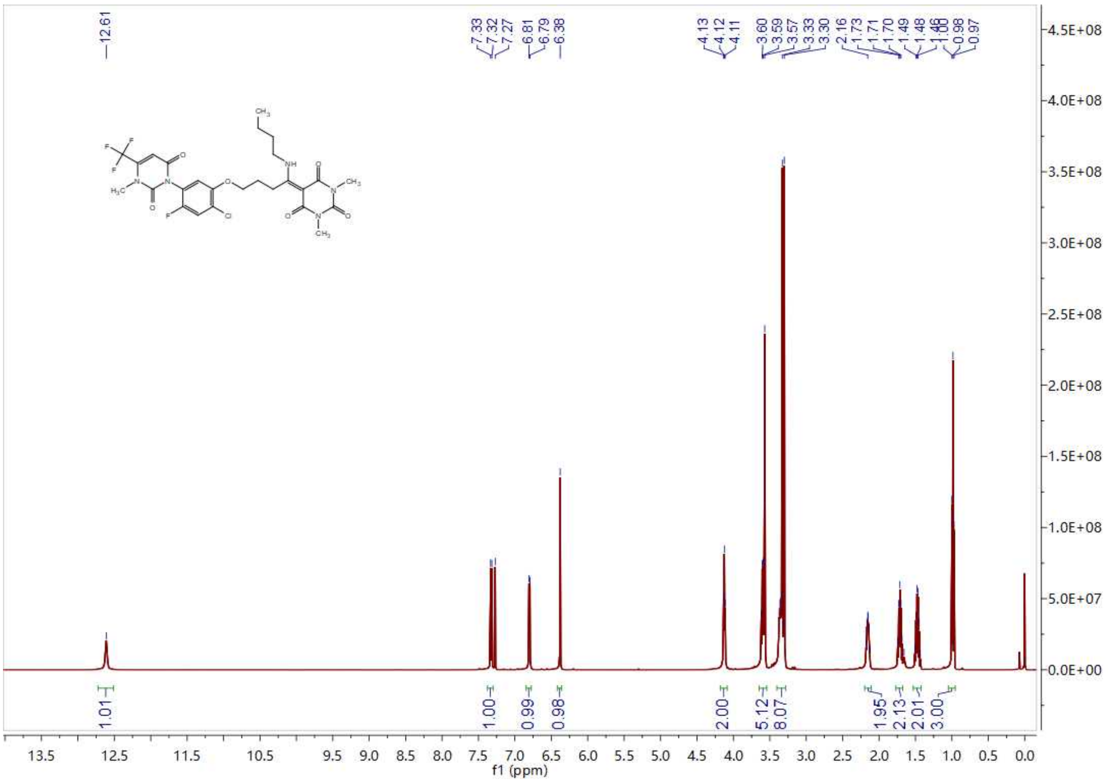

Figure S10 ^{1}HNMR of the target compound BA-4

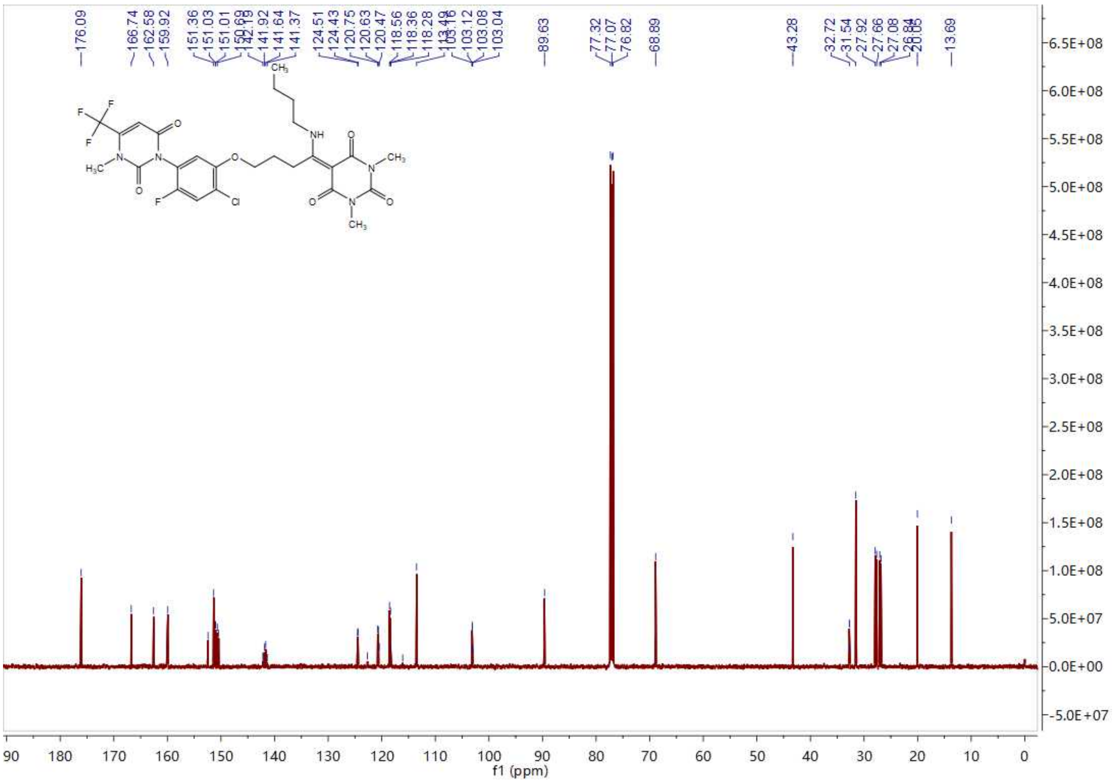

Figure S11 ^{13}CNMR of the target compound BA-4

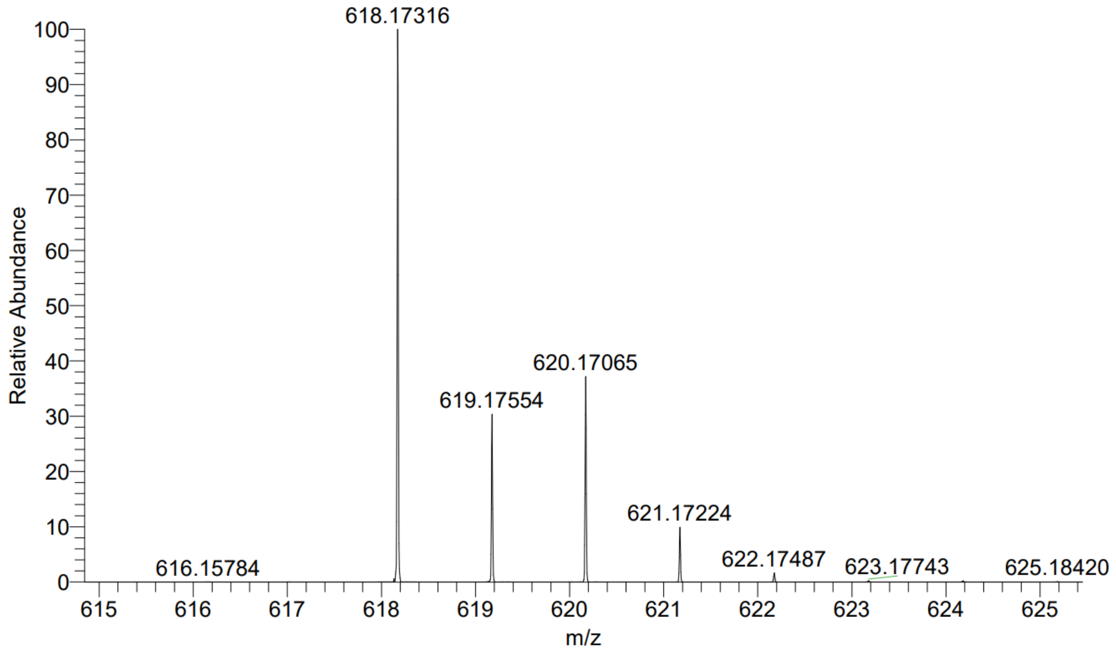

Figure S12 HRMS of the target compound BA-4

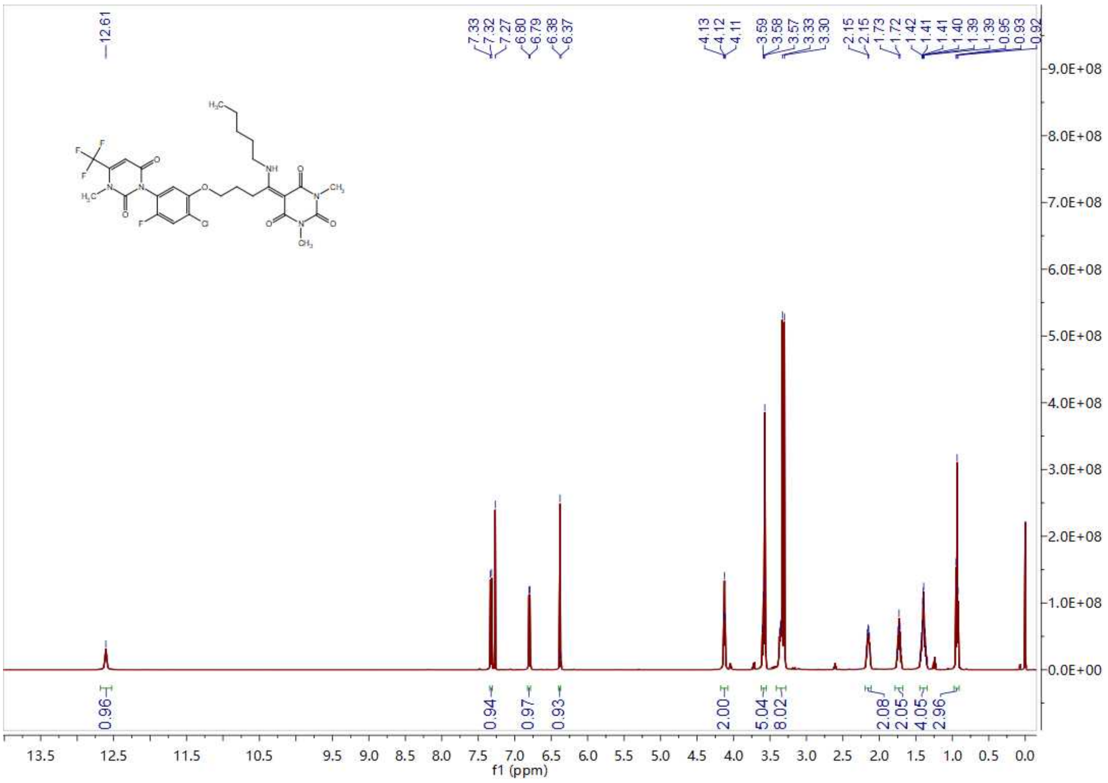

Figure S13 ^{1}HNMR of the target compound BA-5

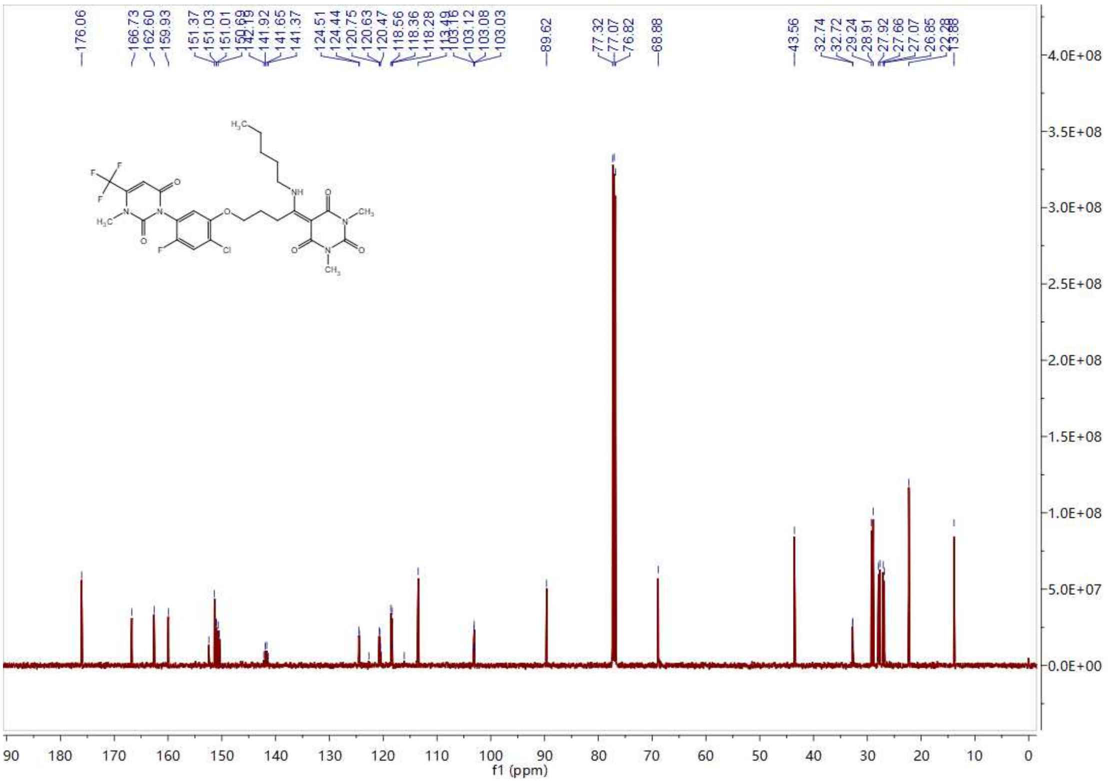

Figure S14 ^{13}CNMR of the target compound BA-5

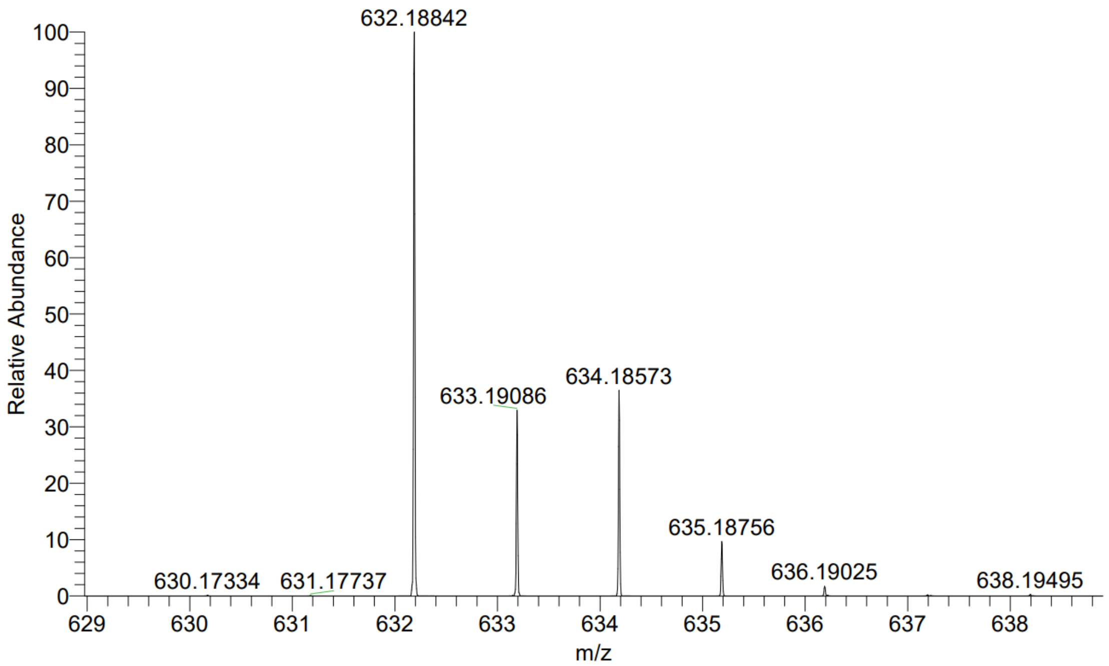

Figure S15 HRMS of the target compound BA-5

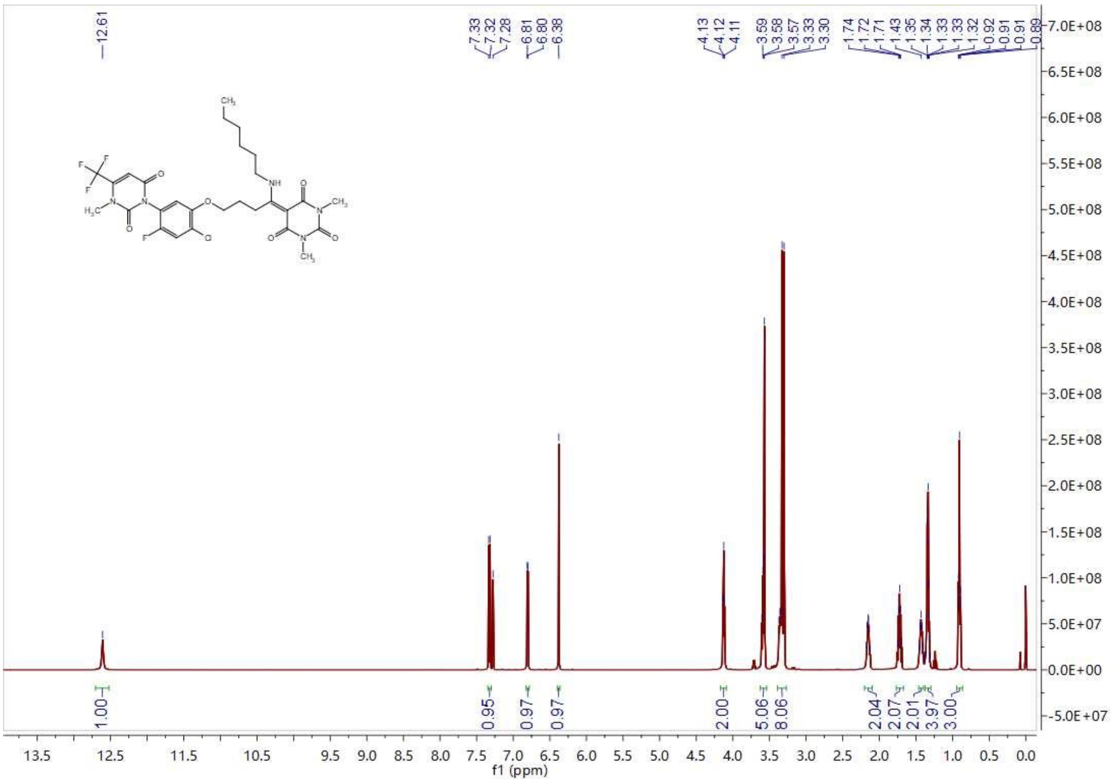

Figure S16 ^{1}HNMR of the target compound BA-6

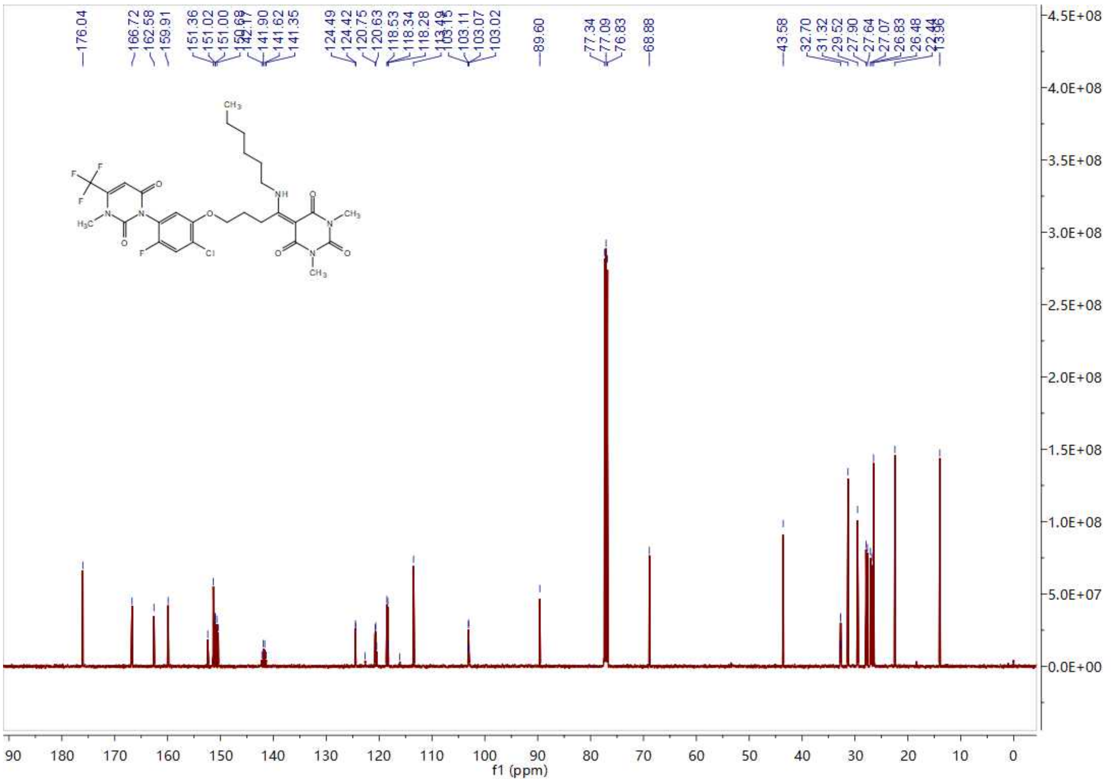

Figure S17 ^{13}CNMR of the target compound BA-6

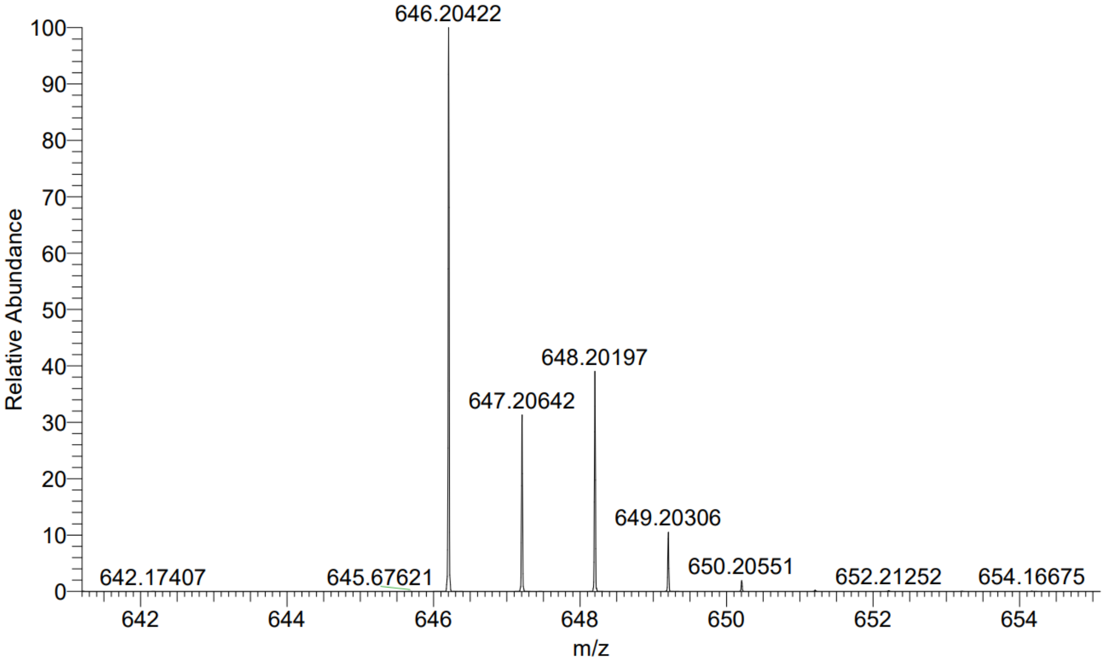

Figure S18 HRMS of the target compound BA-6

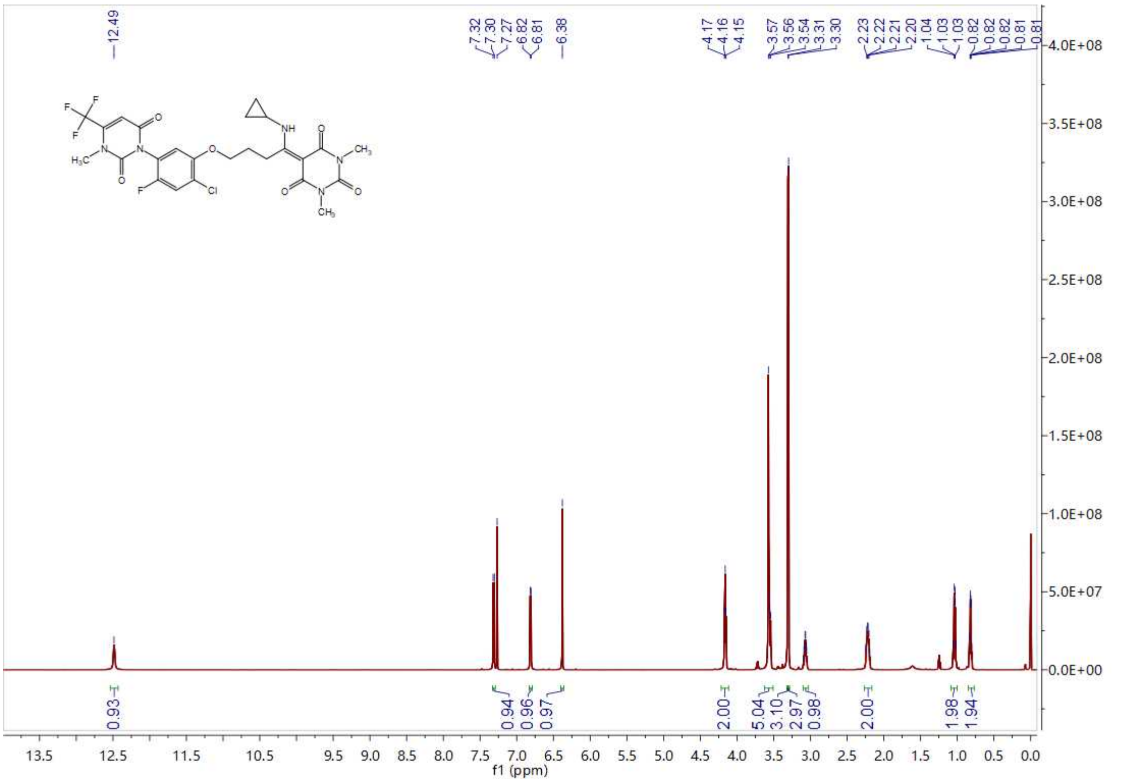

Figure S19 ^{1}HNMR of the target compound BA-7

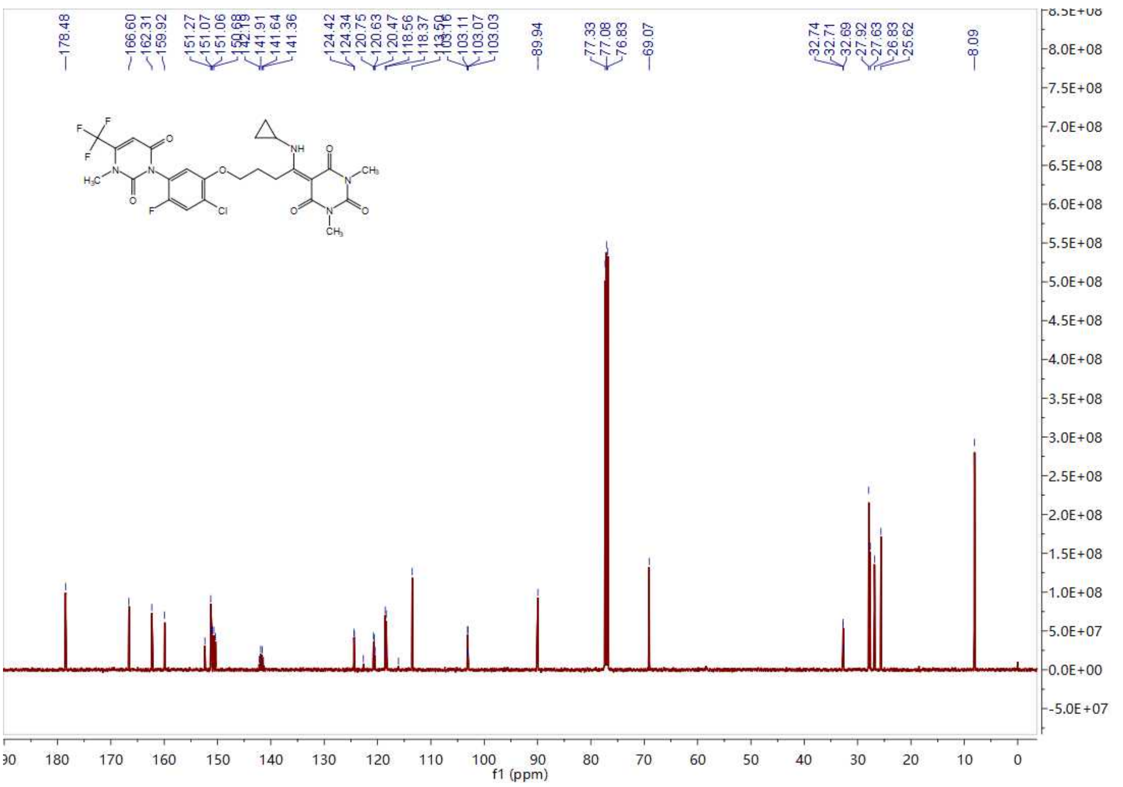

Figure S20 ^{13}CNMR of the target compound BA-7

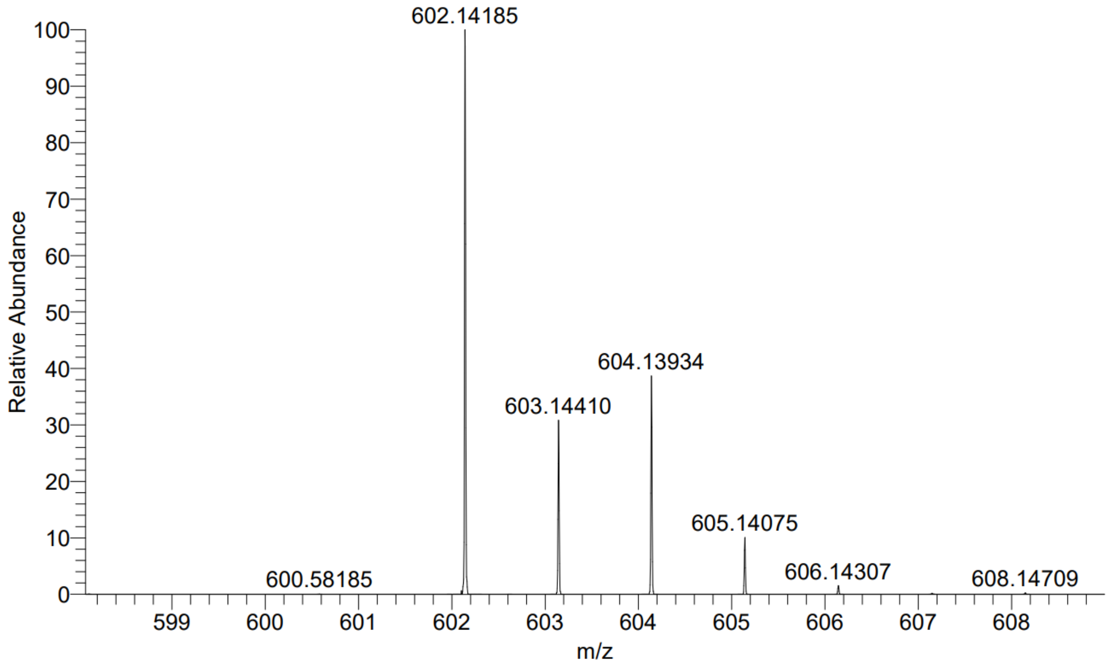

Figure S21 HRMS of the target compound BA-7

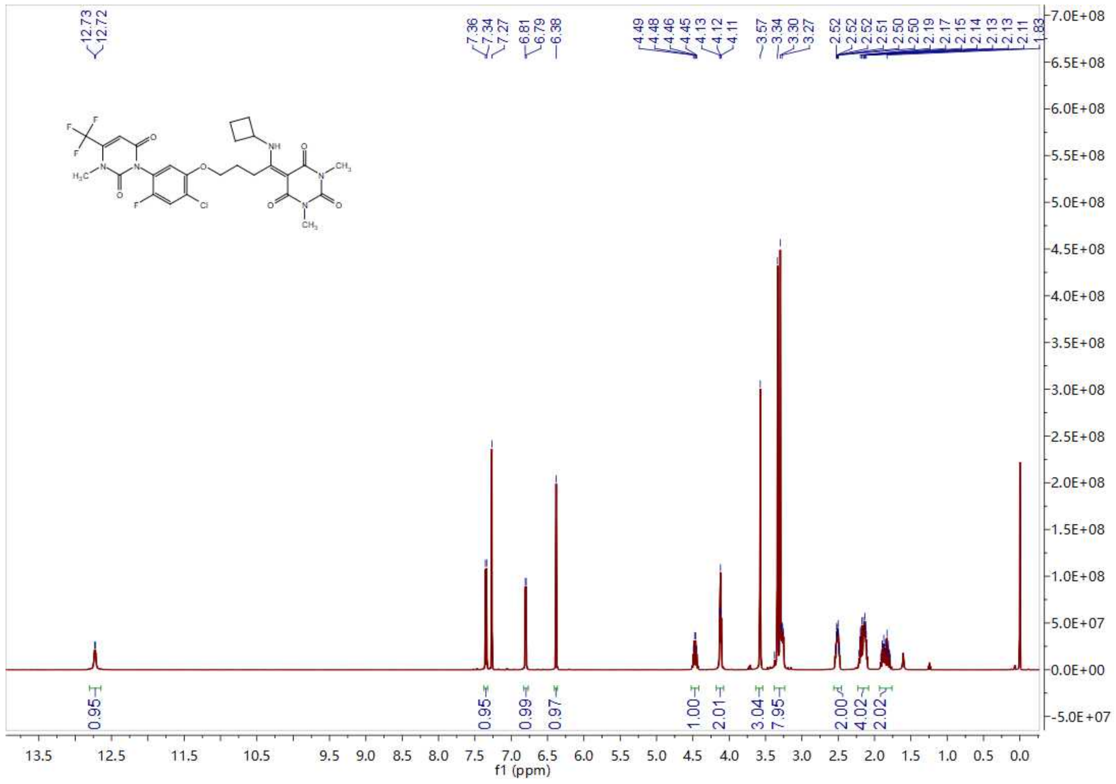

Figure S22 ^{1}HNMR of the target compound BA-8

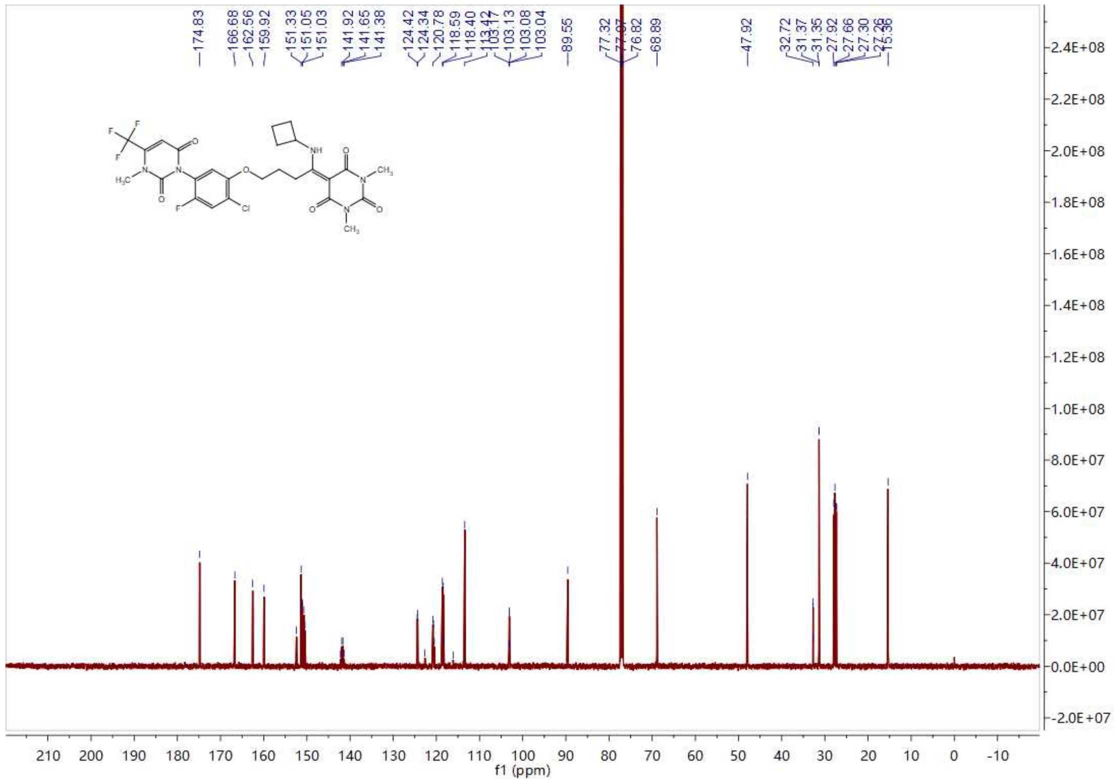

Figure S23 ^{13}CNMR of the target compound BA-8

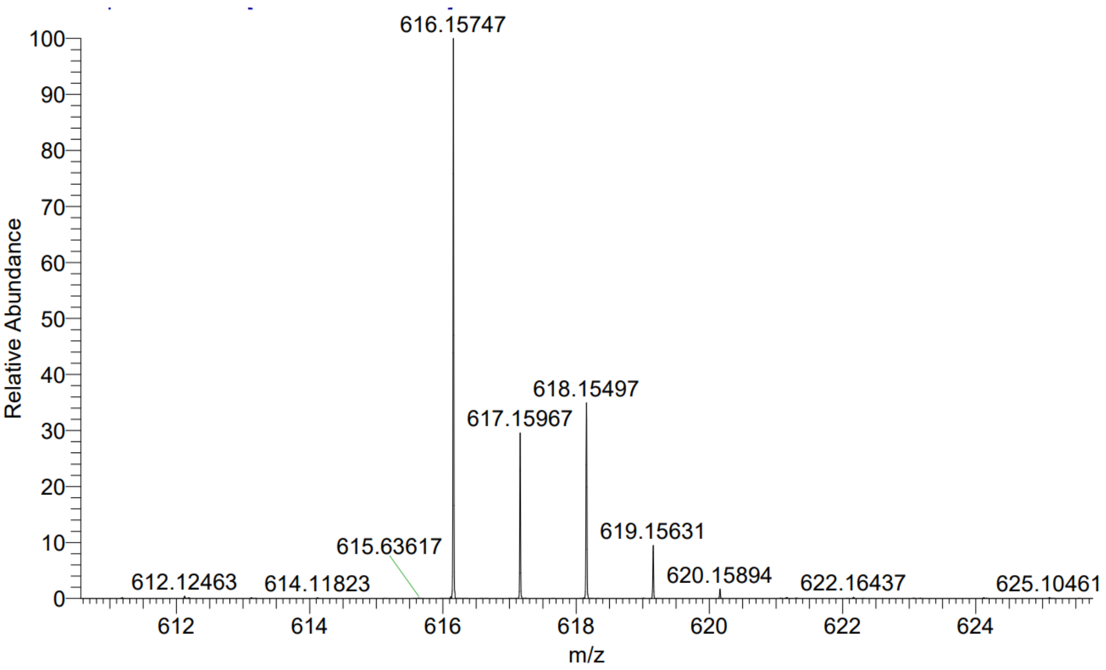

Figure S24 HRMS of the target compound BA-8

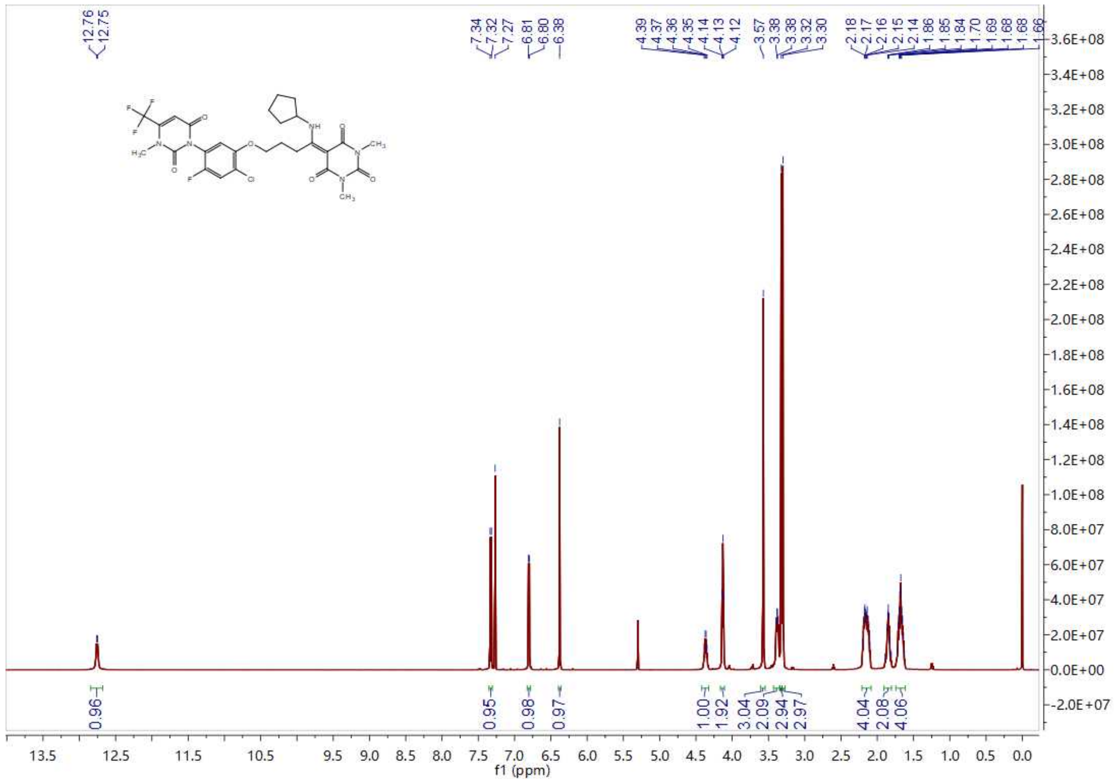

Figure S25 ^{1}HNMR of the target compound BA-9

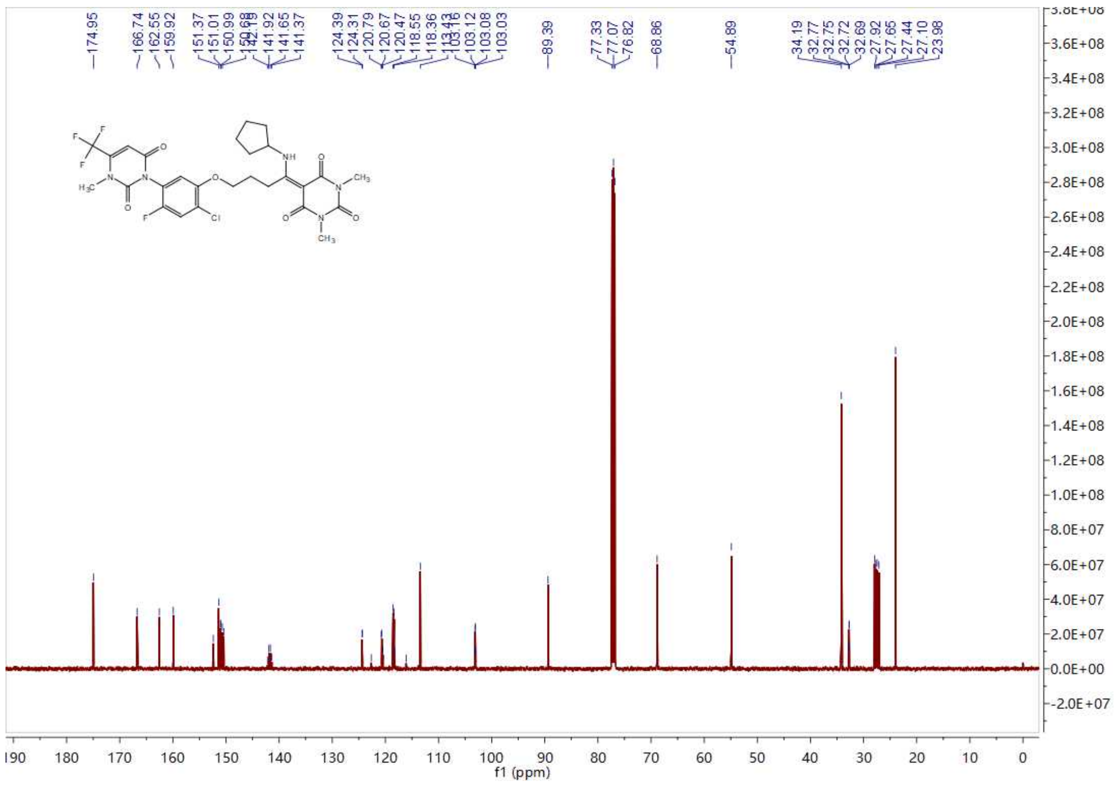

Figure S26 ^{13}CNMR of the target compound BA-9

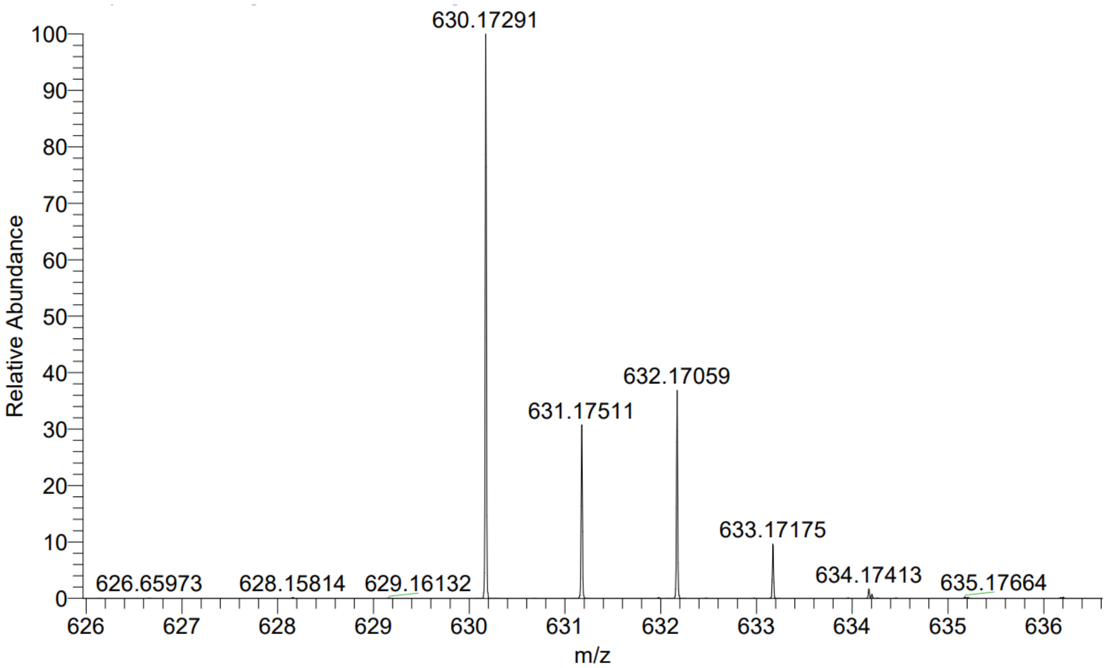

Figure S27 HRMS of the target compound BA-9

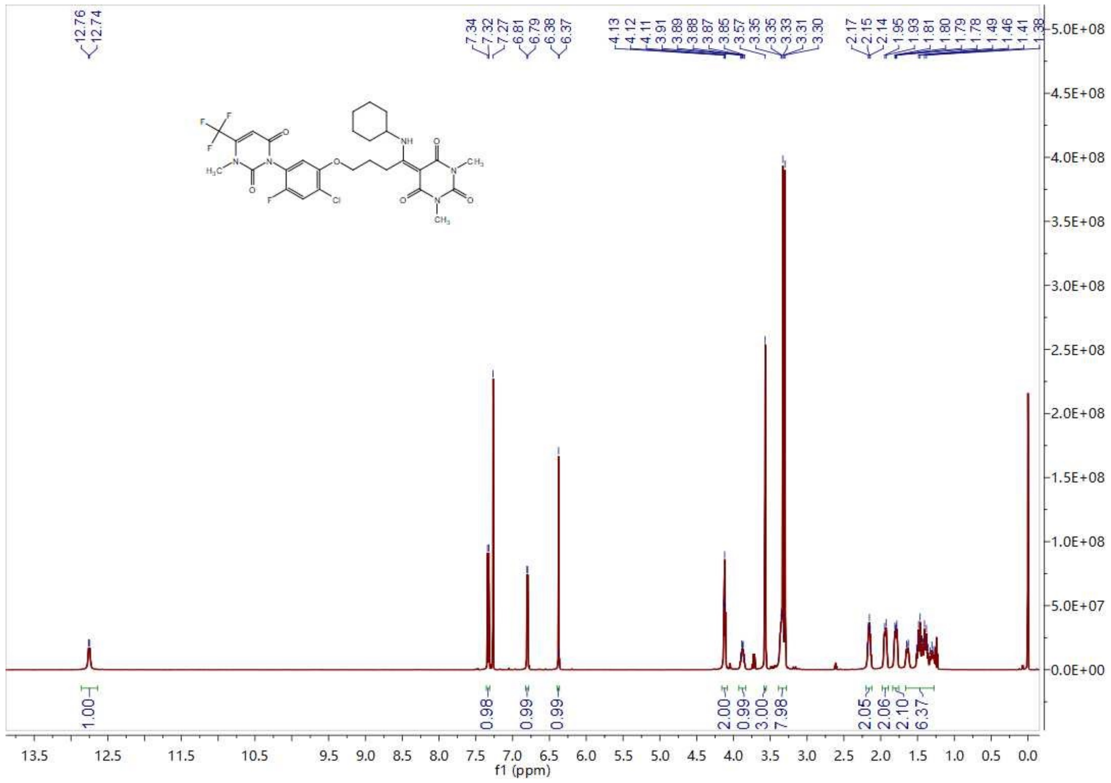

Figure S28 ^{1}HNMR of the target compound BA-10

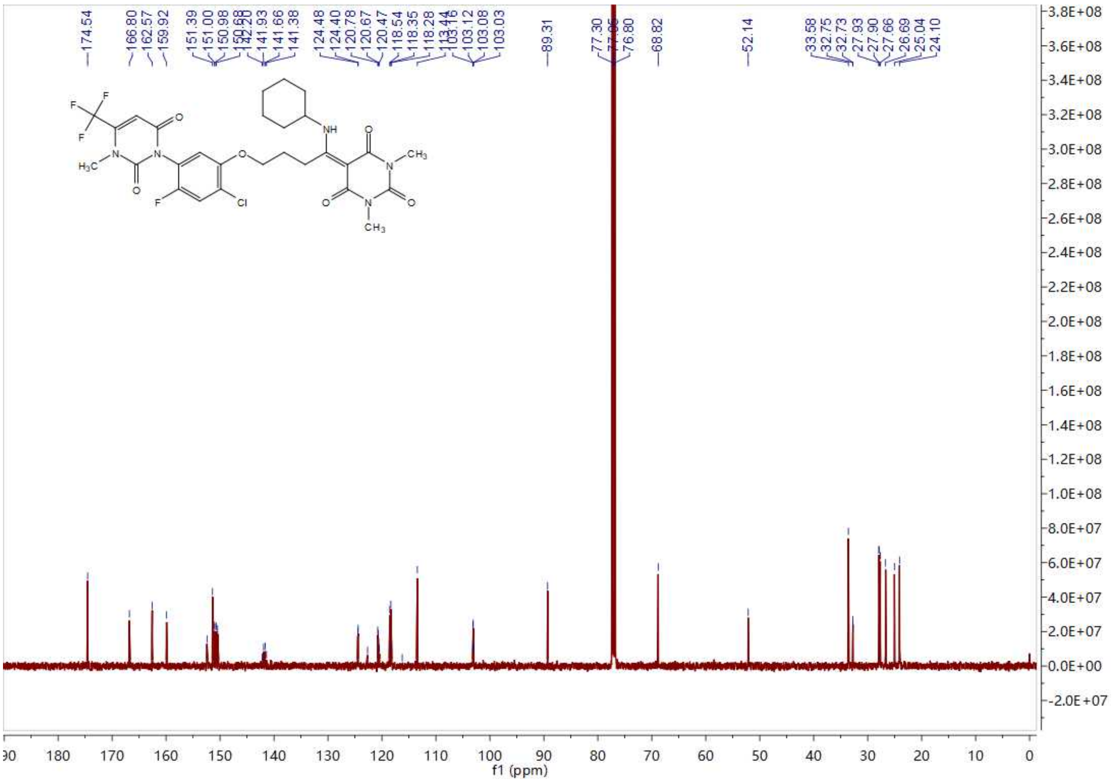

Figure S29 ^{13}CNMR of the target compound BA-10

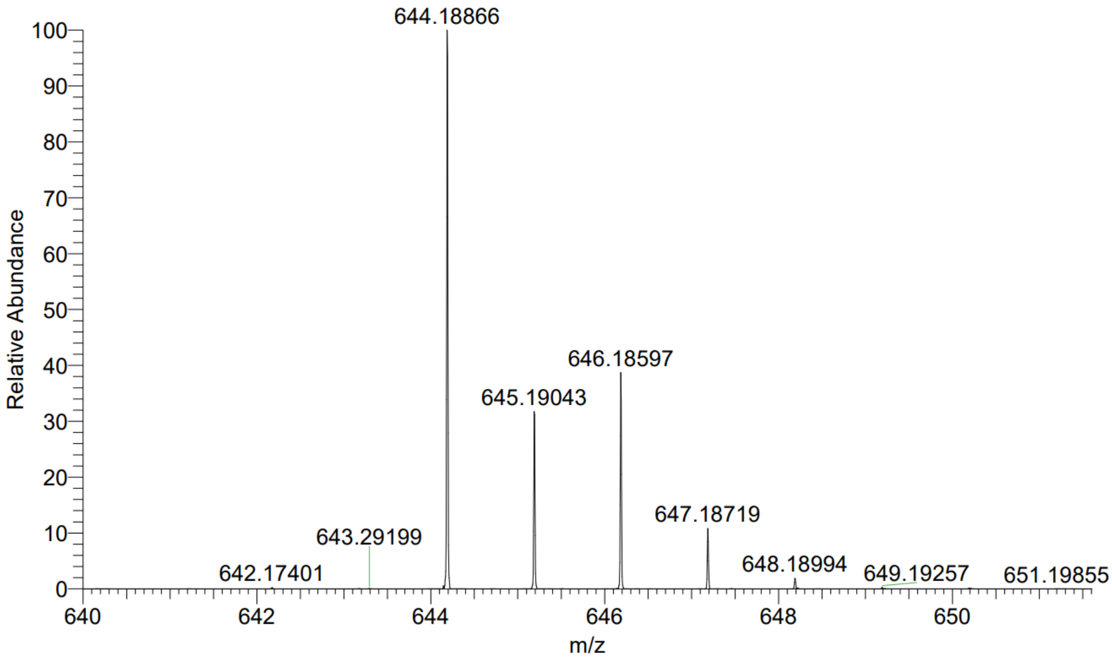

Figure S30 HRMS of the target compound BA-10

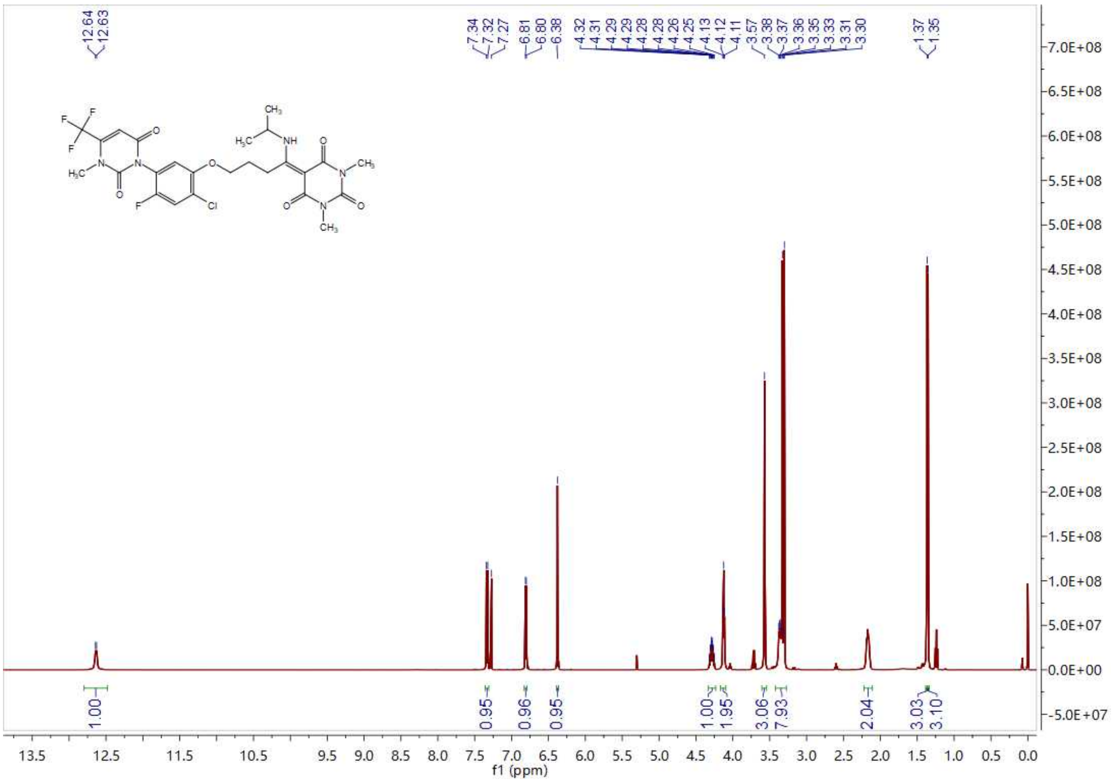

Figure S31 ^{1}HNMR of the target compound BA-11

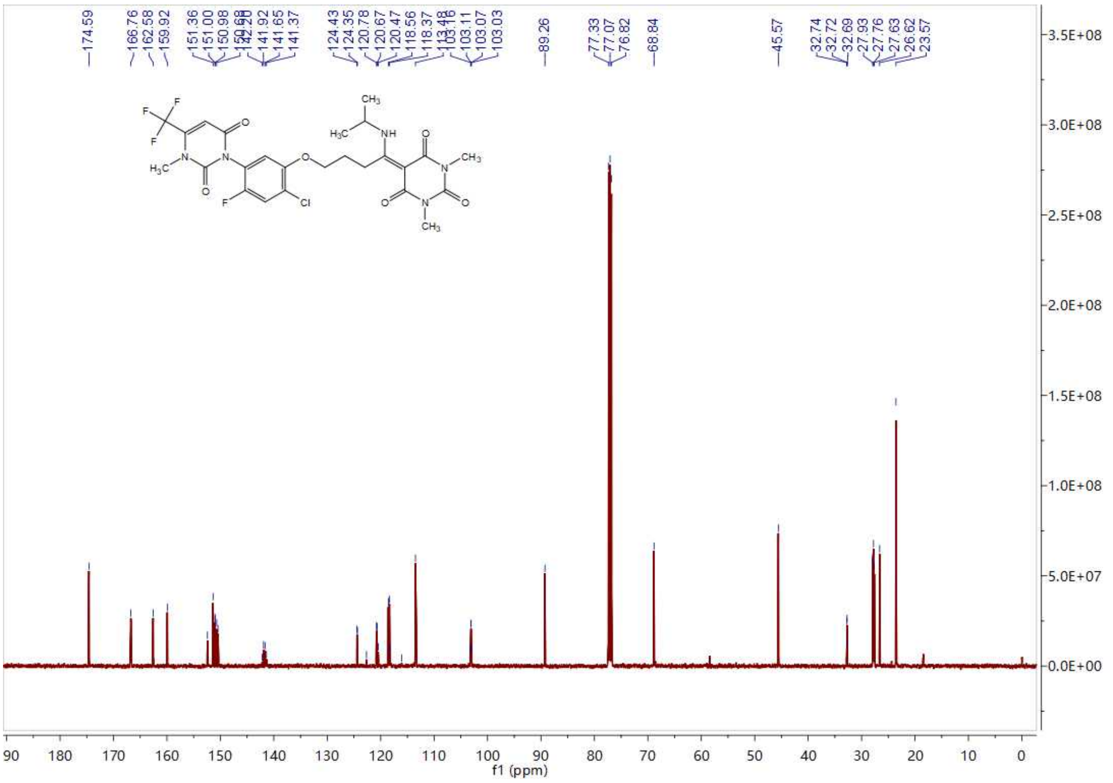

Figure S32 ^{13}CNMR of the target compound BA-11

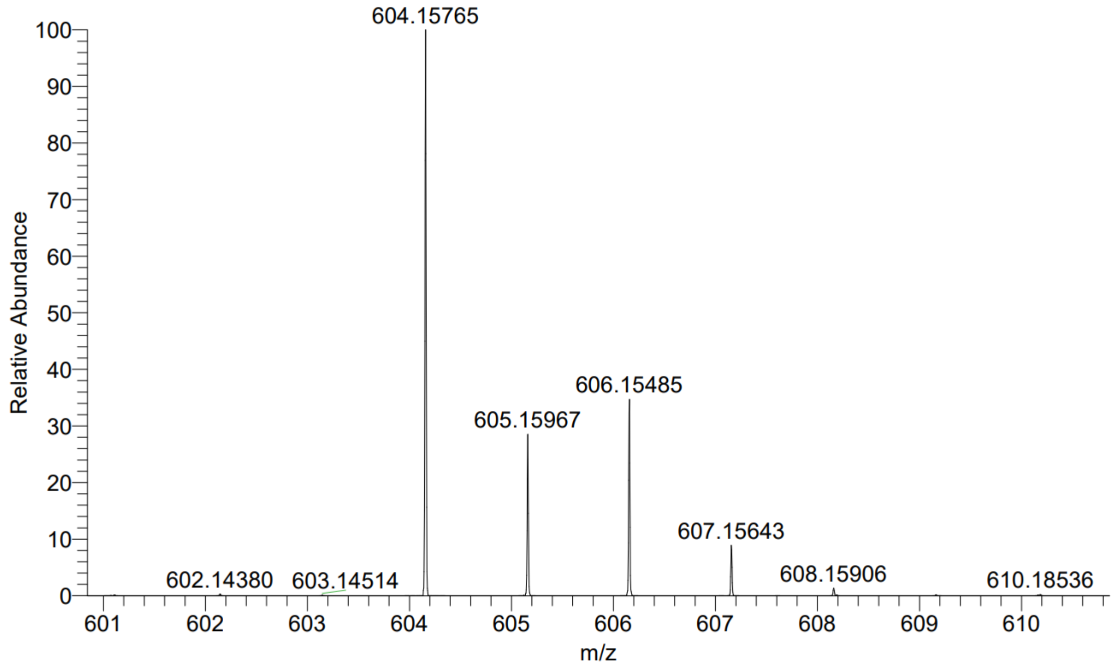

Figure S33 HRMS of the target compound BA-11

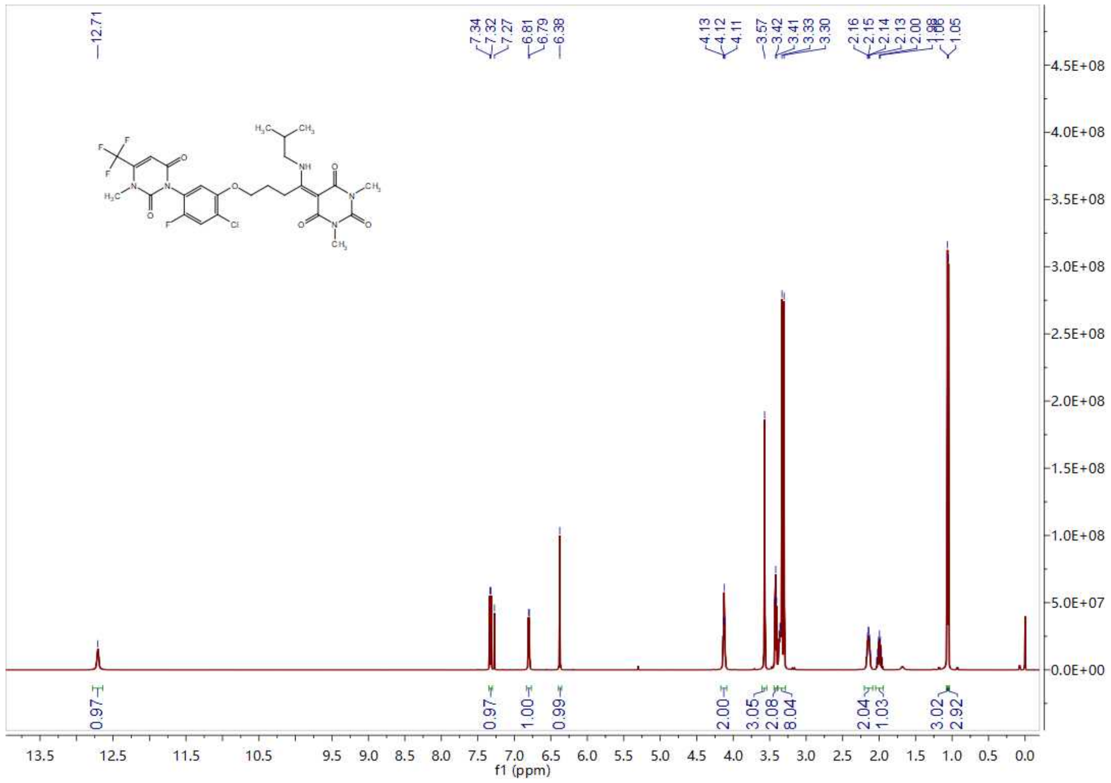

Figure S34 ^{1}HNMR of the target compound BA-12

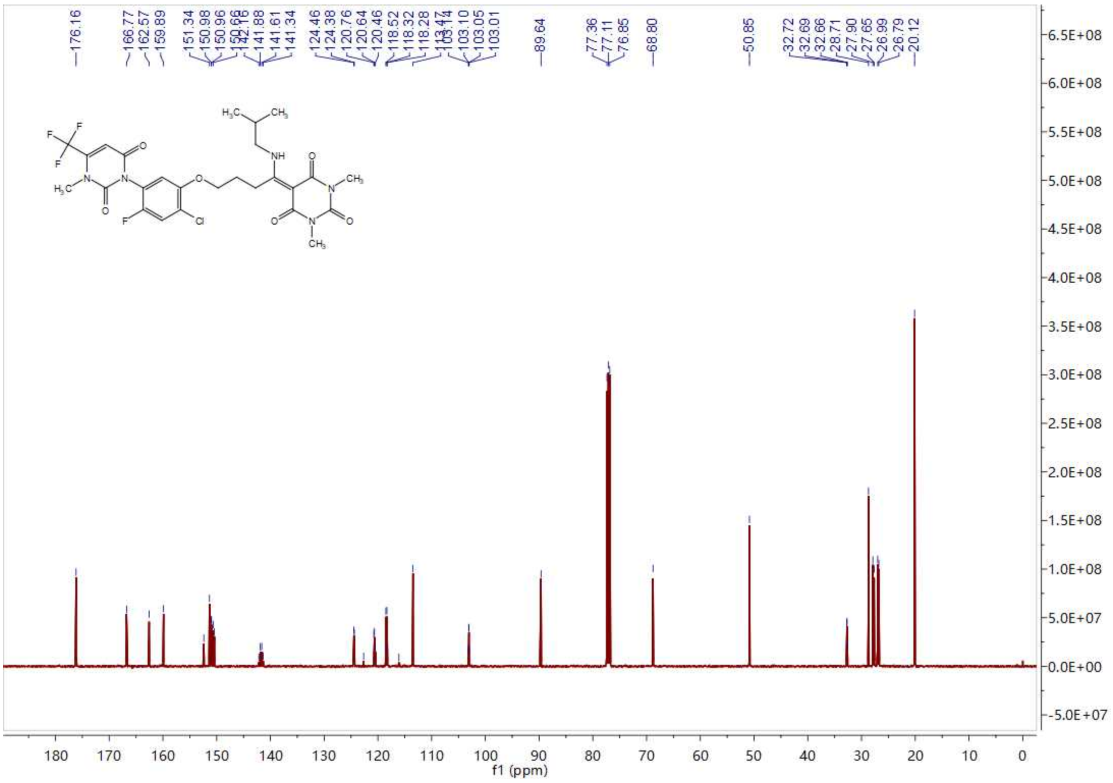

Figure S35 ^{13}CNMR of the target compound BA-12

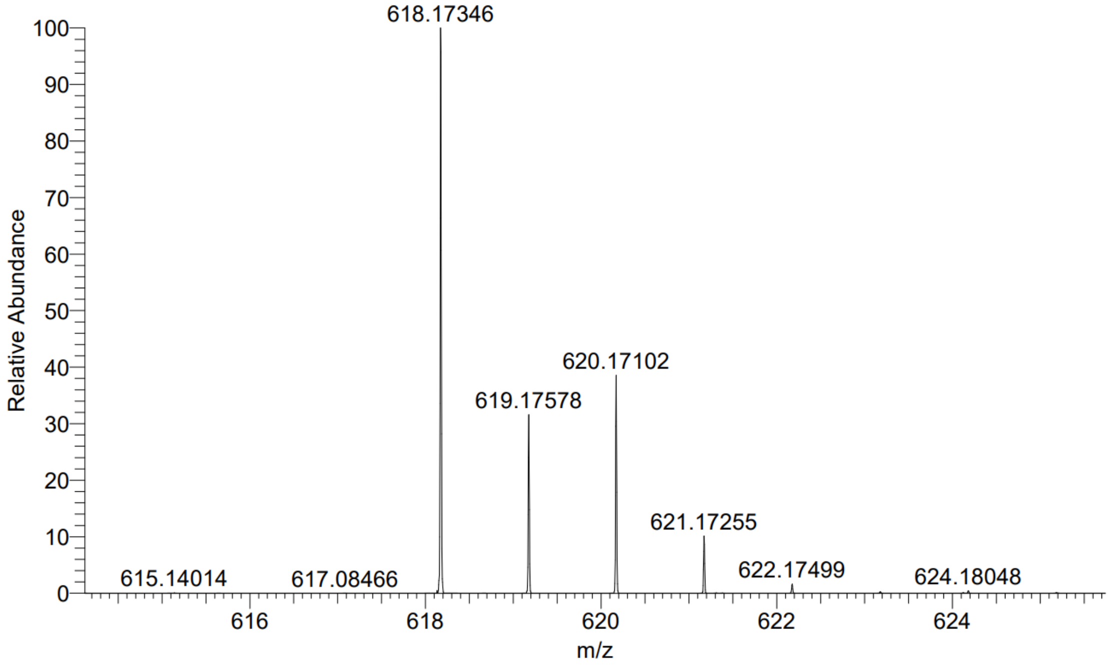

Figure S36 HRMS of the target compound BA-12

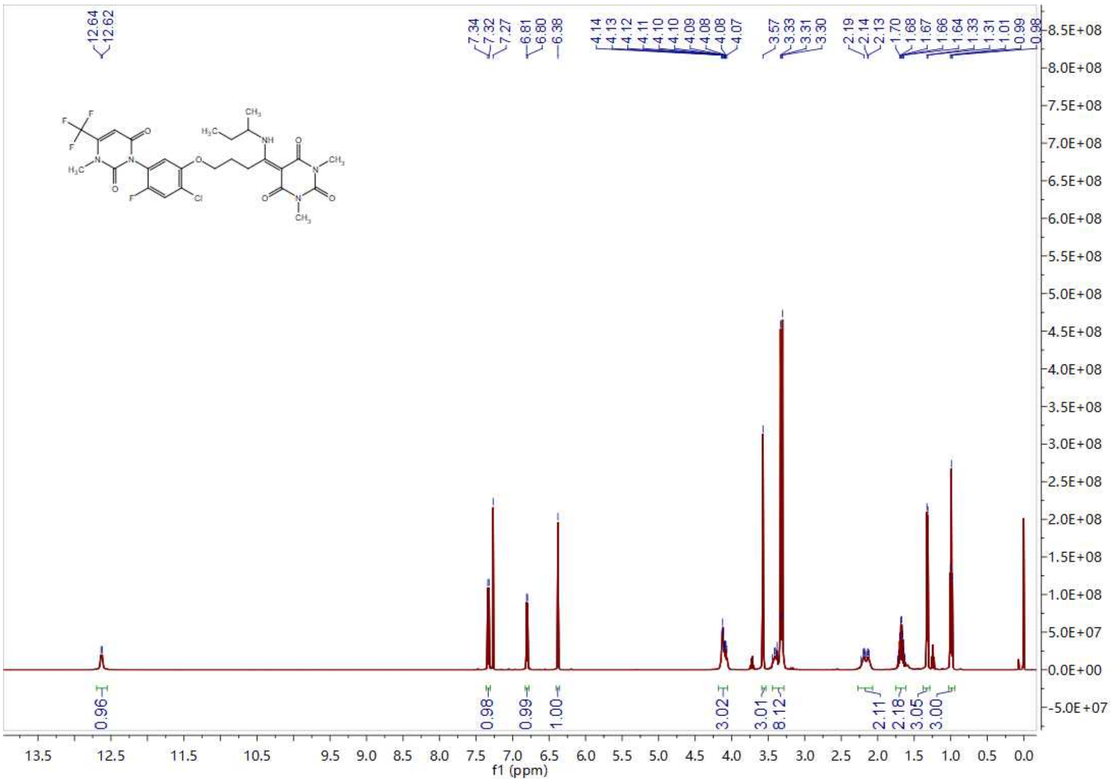

Figure S37 ¹HNMR of the target compound BA-13

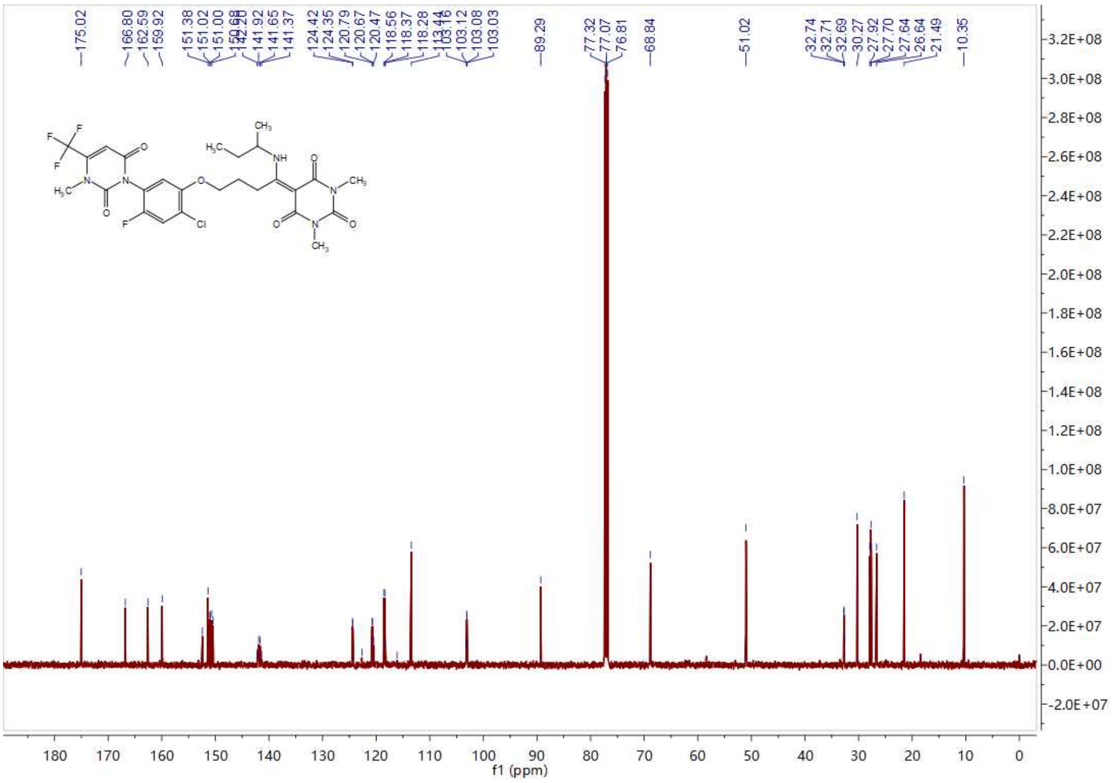

Figure S38 ¹³CNMR of the target compound BA-13

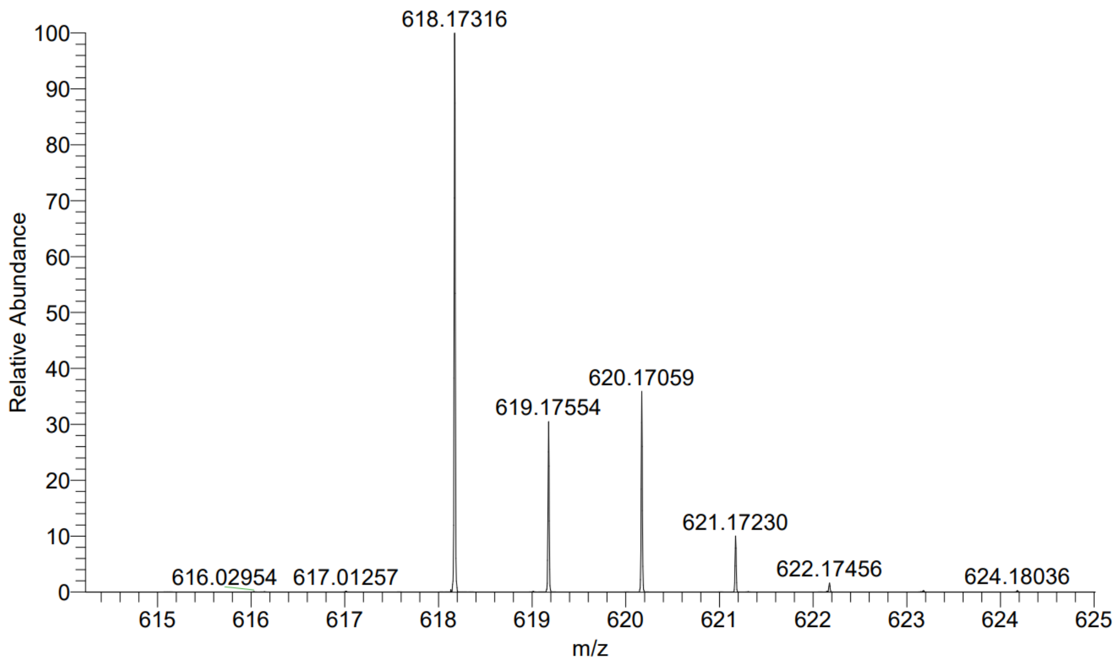

Figure S39 HRMS of the target compound BA-13

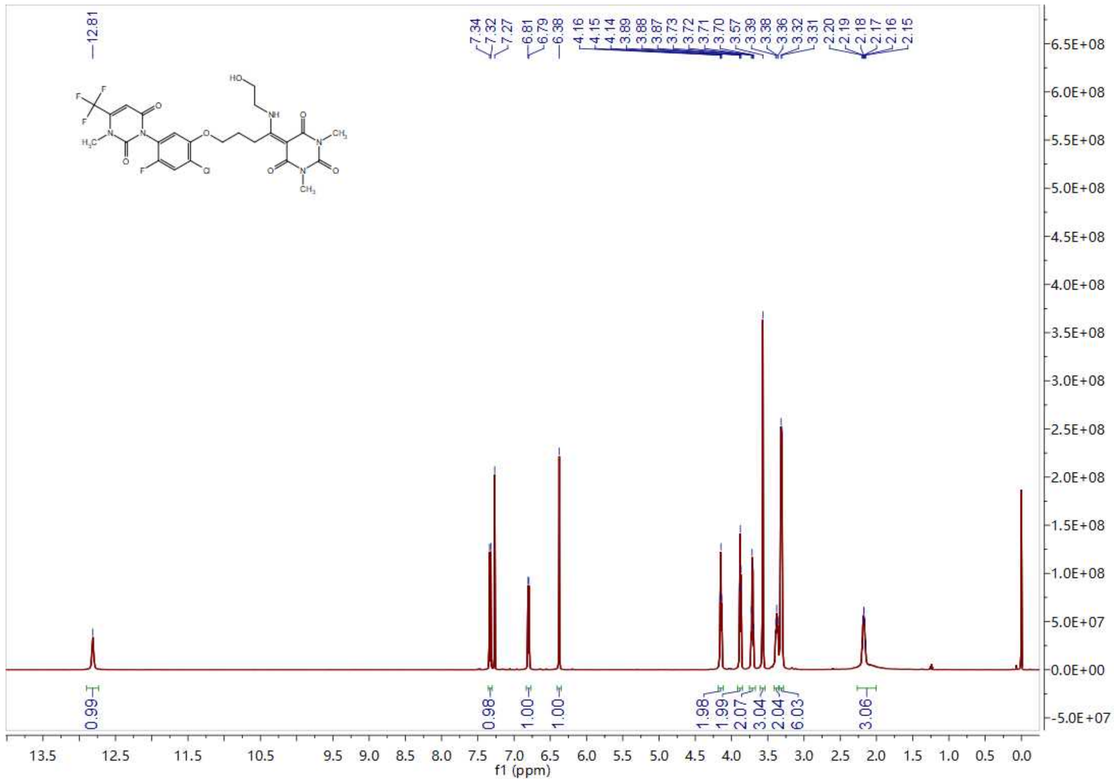

Figure S40 ^{1}HNMR of the target compound BA-14

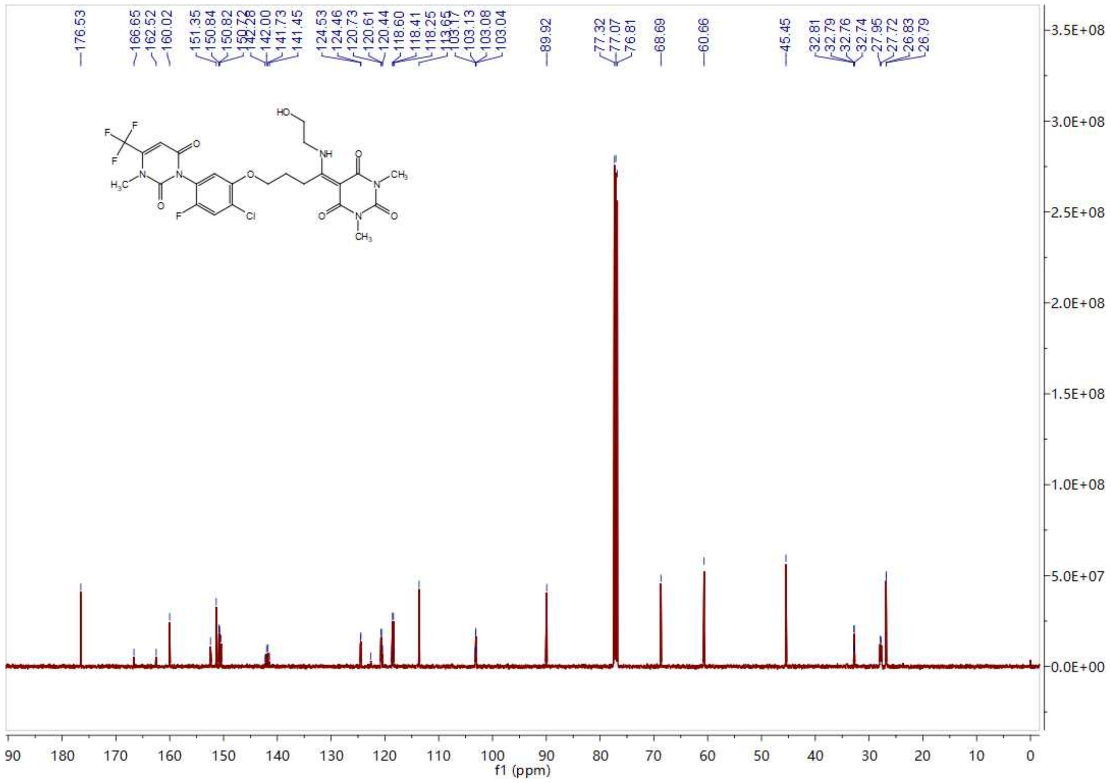

Figure S41 ^{13}CNMR of the target compound BA-14

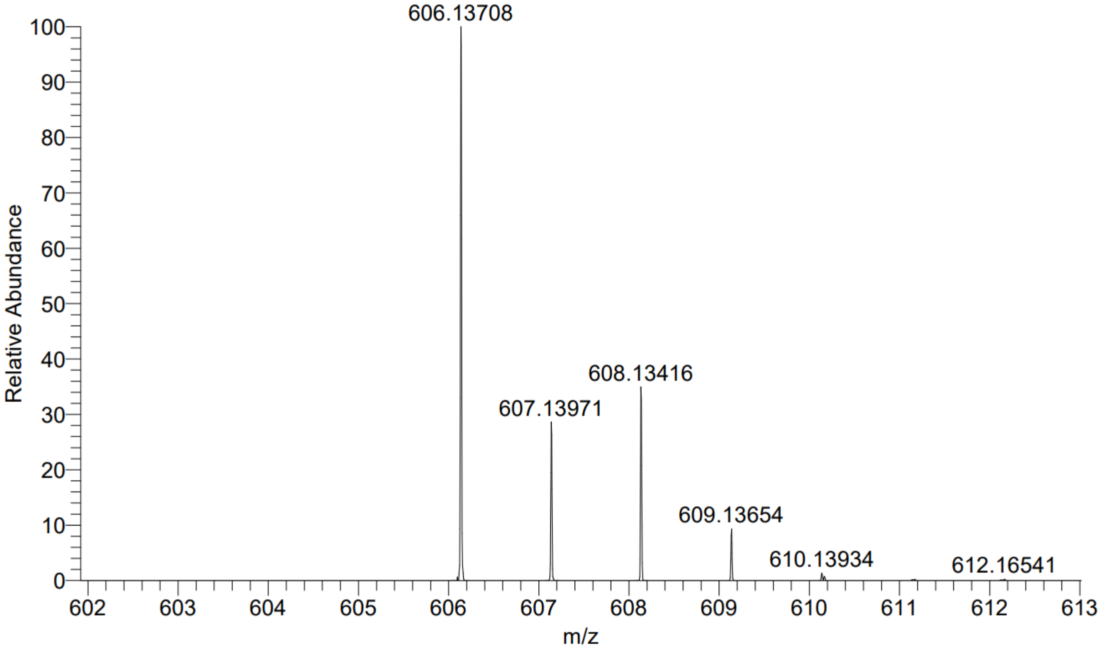

Figure S42 HRMS of the target compound BA-14

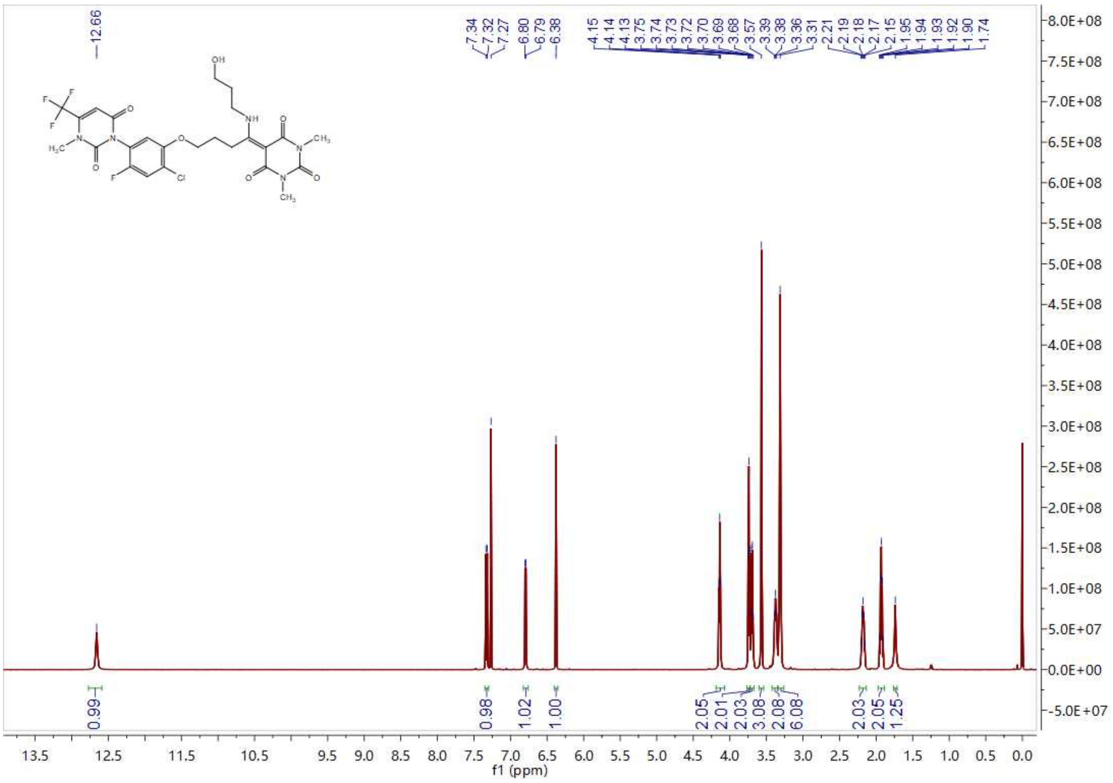

Figure S43 ^{1}HNMR of the target compound BA-15

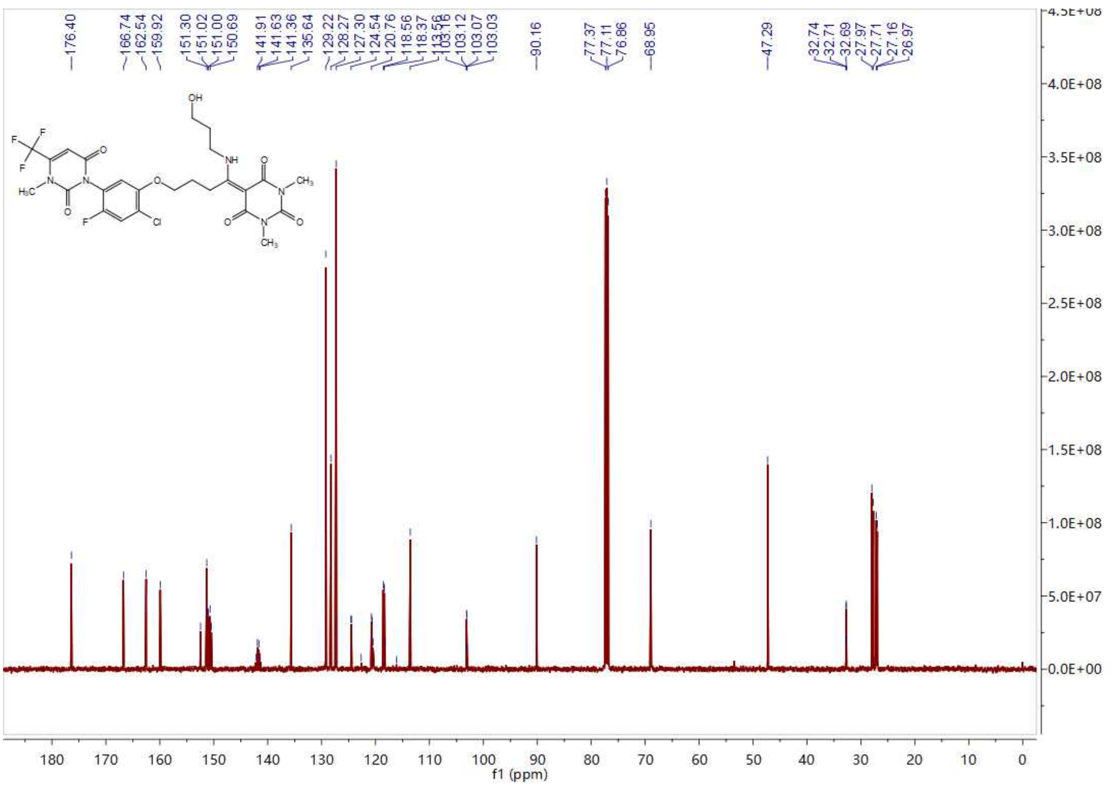

Figure S44 ^{13}CNMR of the target compound BA-15

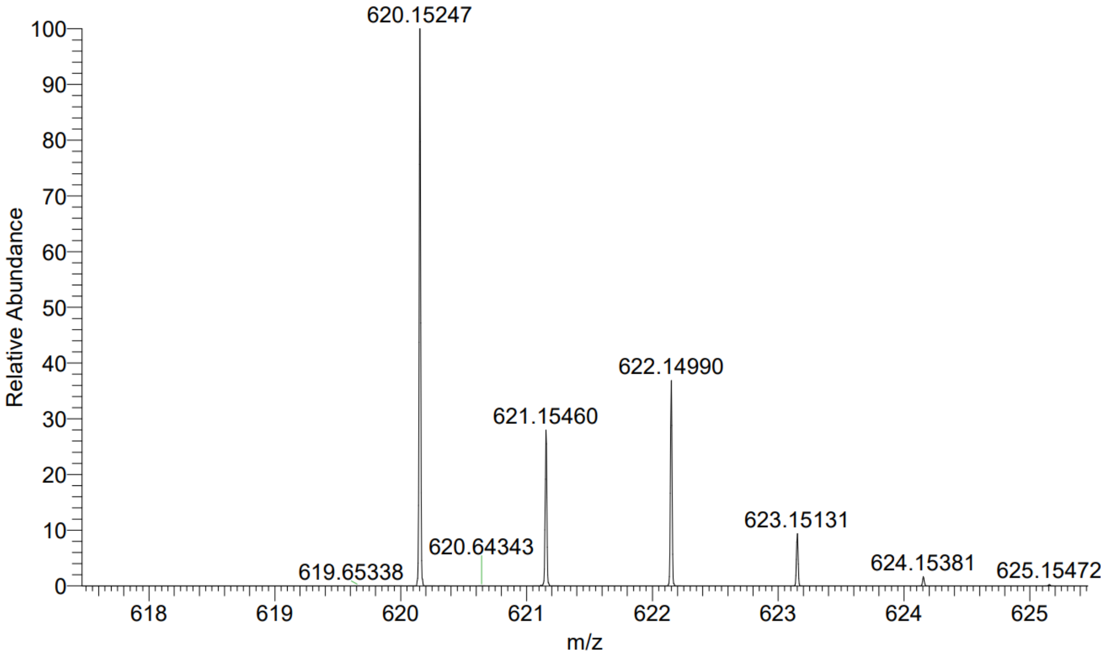

Figure S45 HRMS of the target compound BA-15

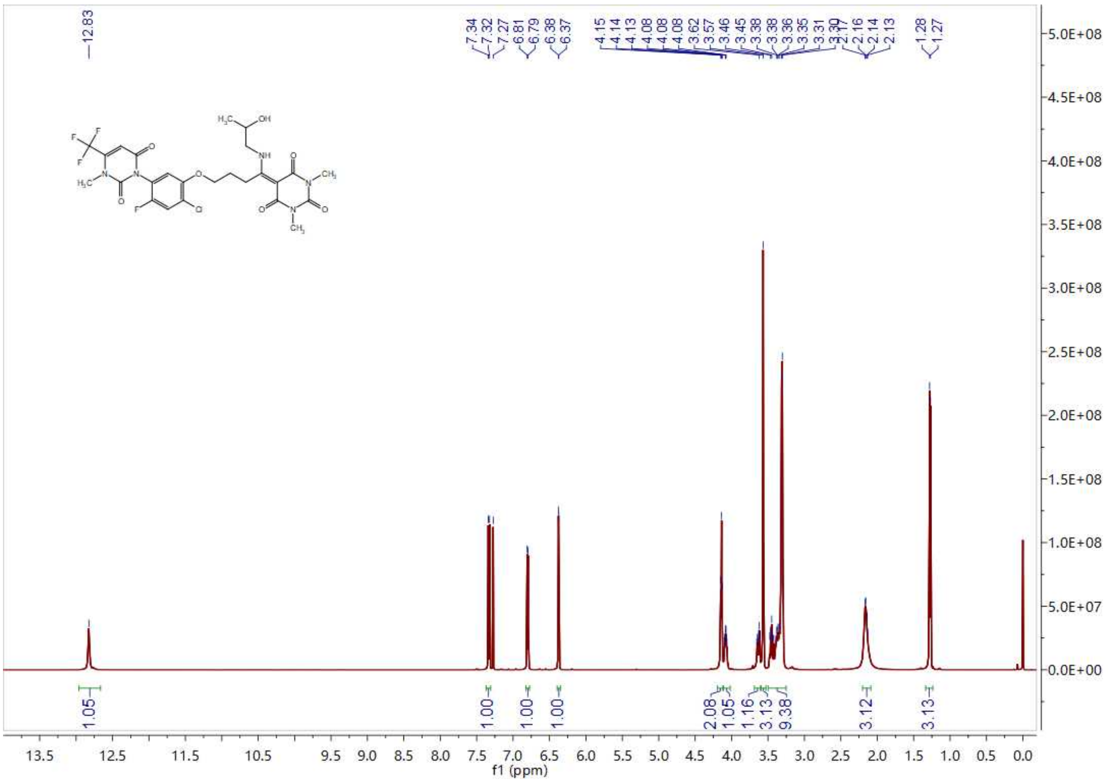

Figure S46 ^{1}HNMR of the target compound BA-16

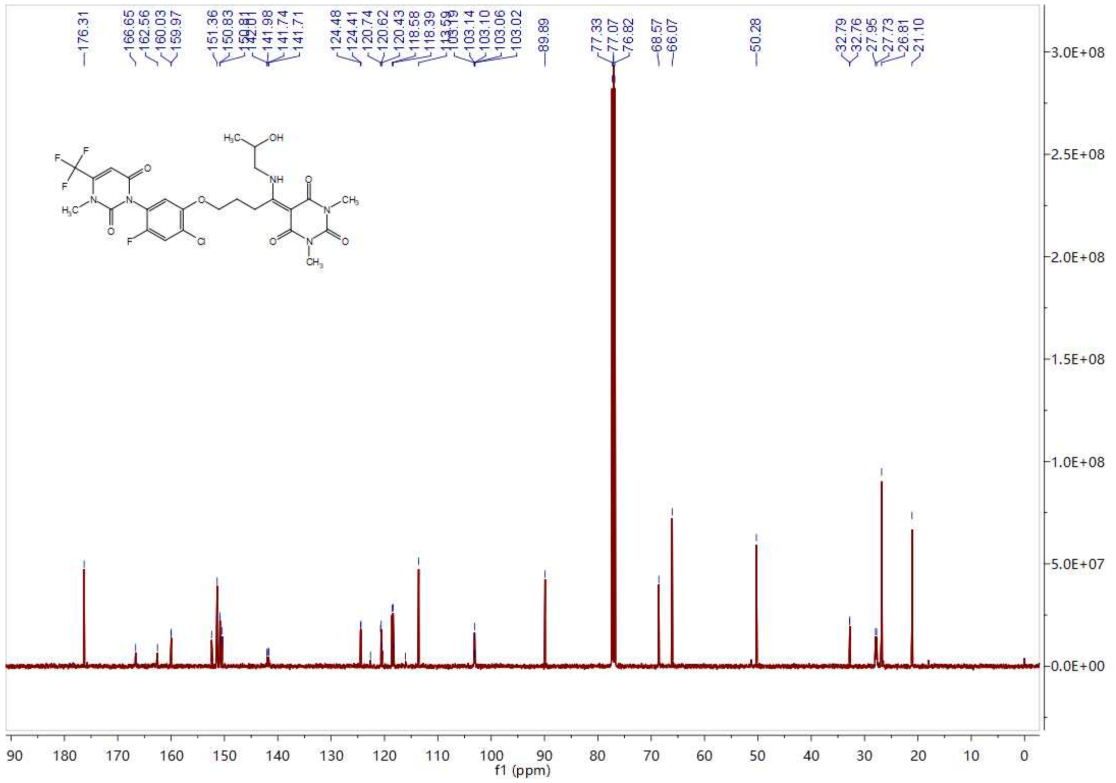

Figure S47 ^{13}CNMR of the target compound BA-16

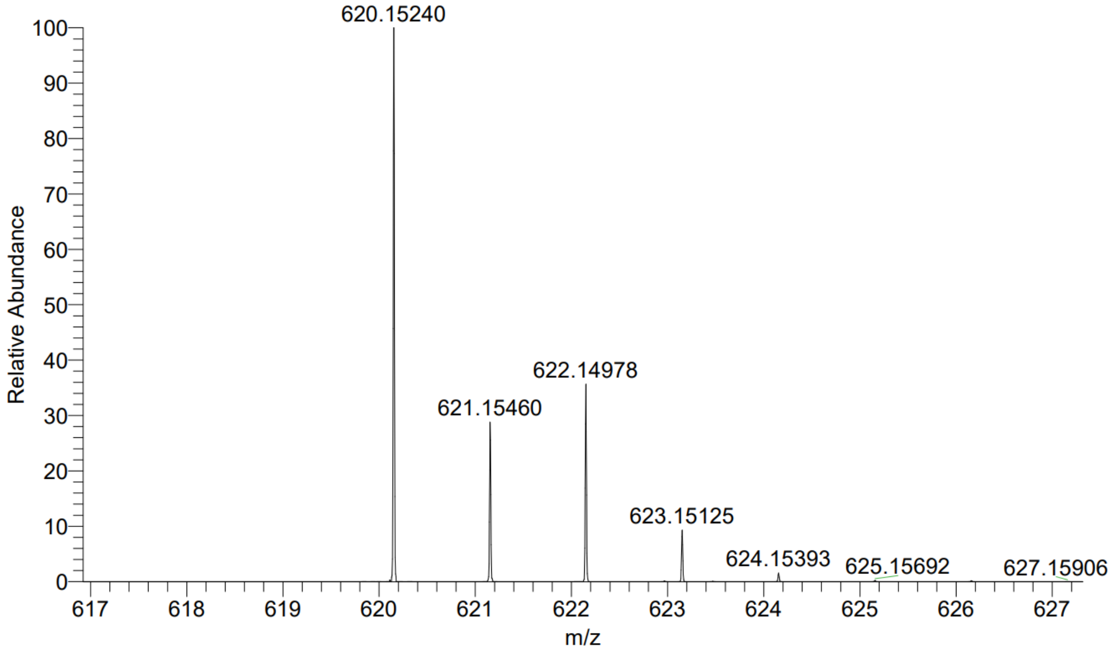

Figure S48 HRMS of the target compound BA-16

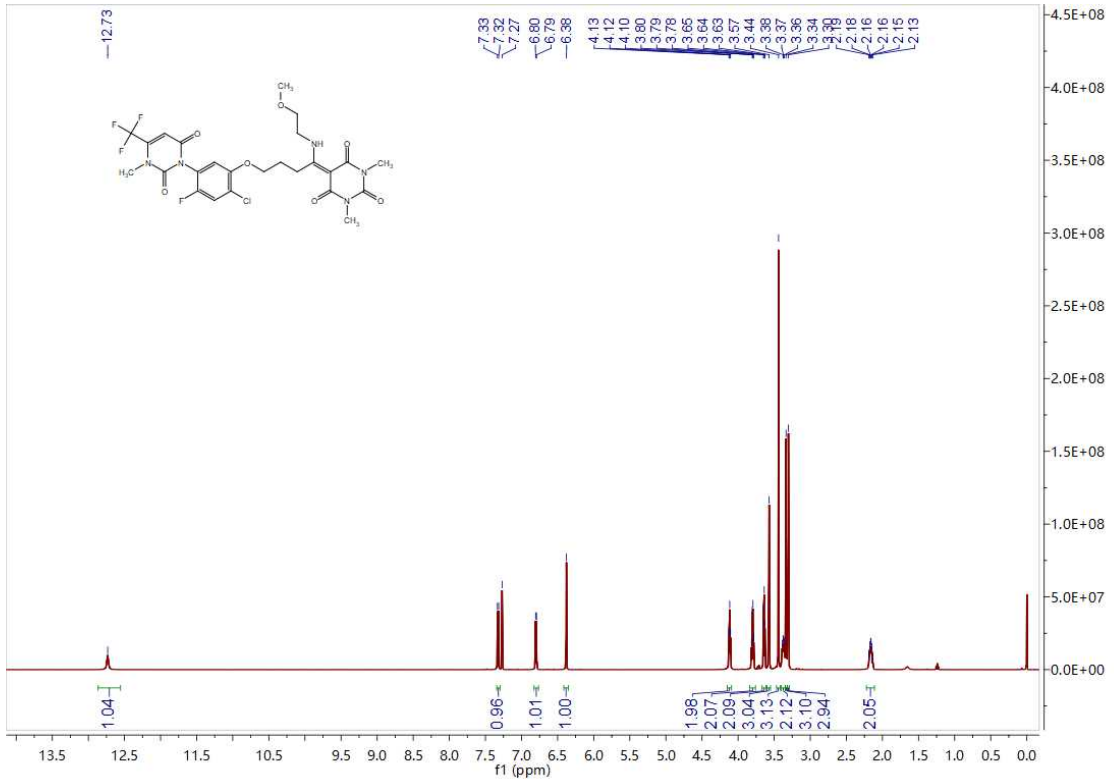

Figure S49 ^{1}HNMR of the target compound BA-17

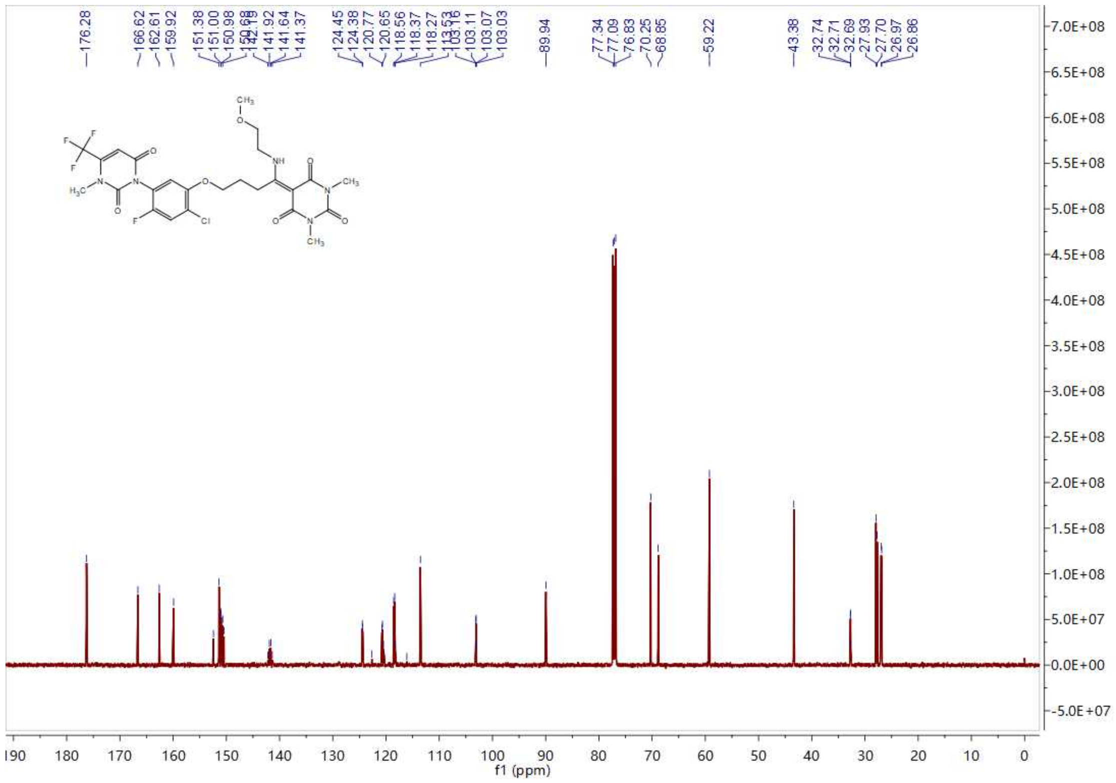

Figure S50 ^{13}CNMR of the target compound BA-17

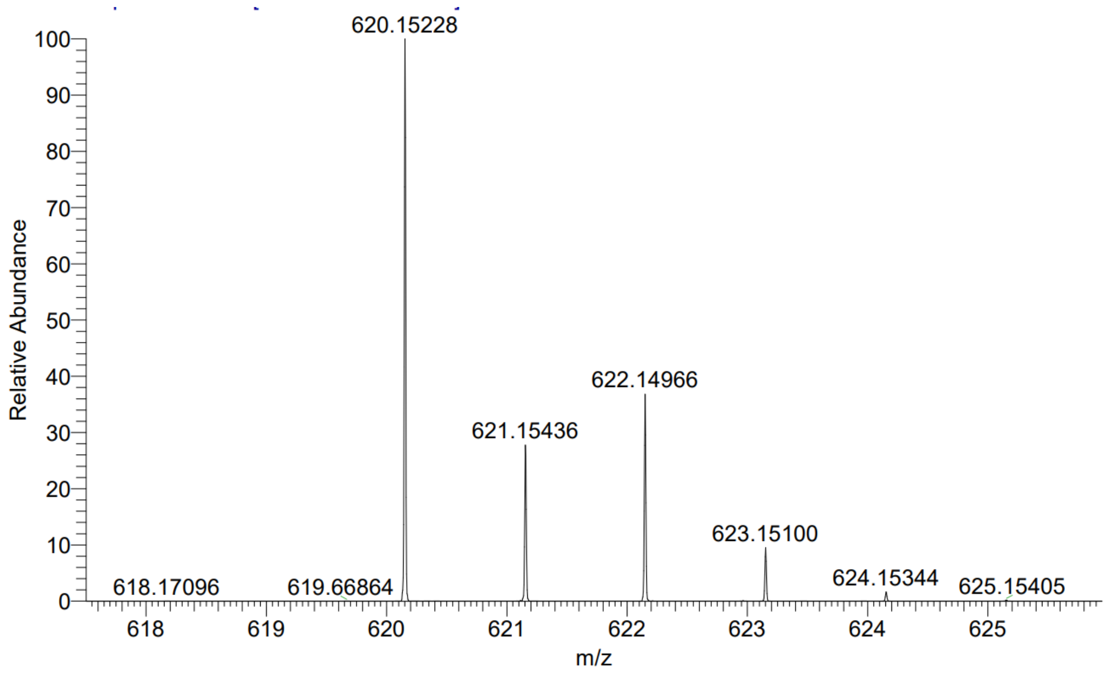

Figure S51 HRMS of the target compound BA-17

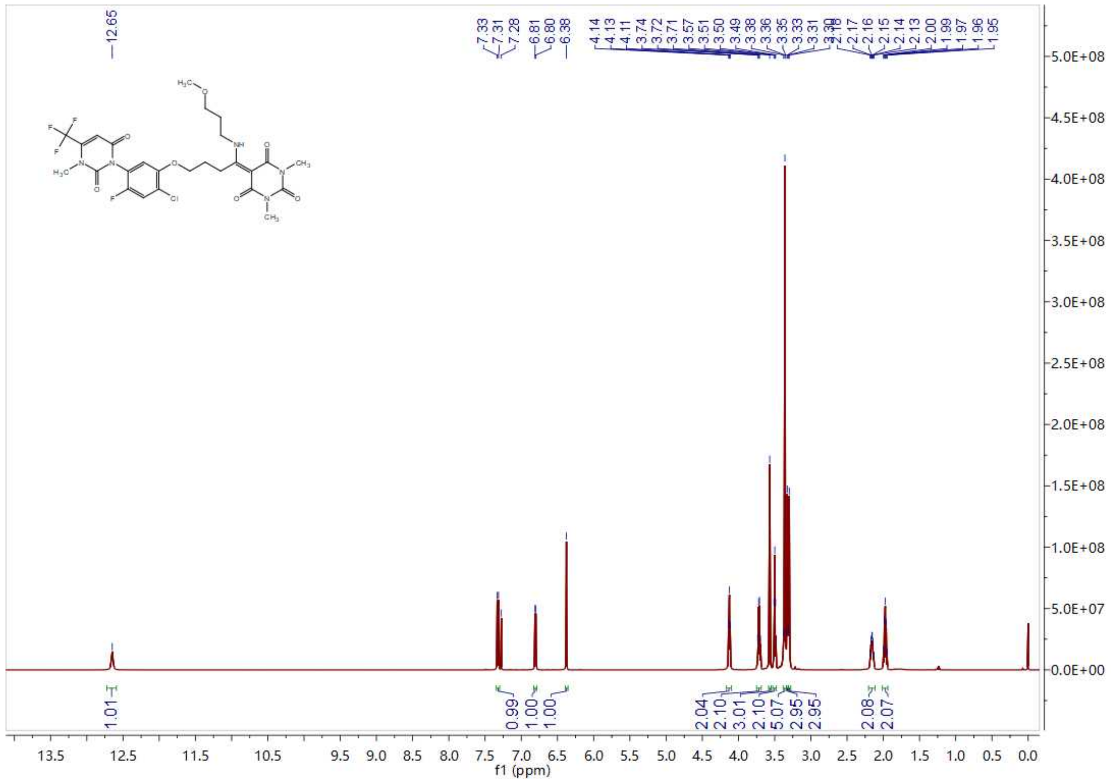

Figure S52 ^{1}HNMR of the target compound BA-18

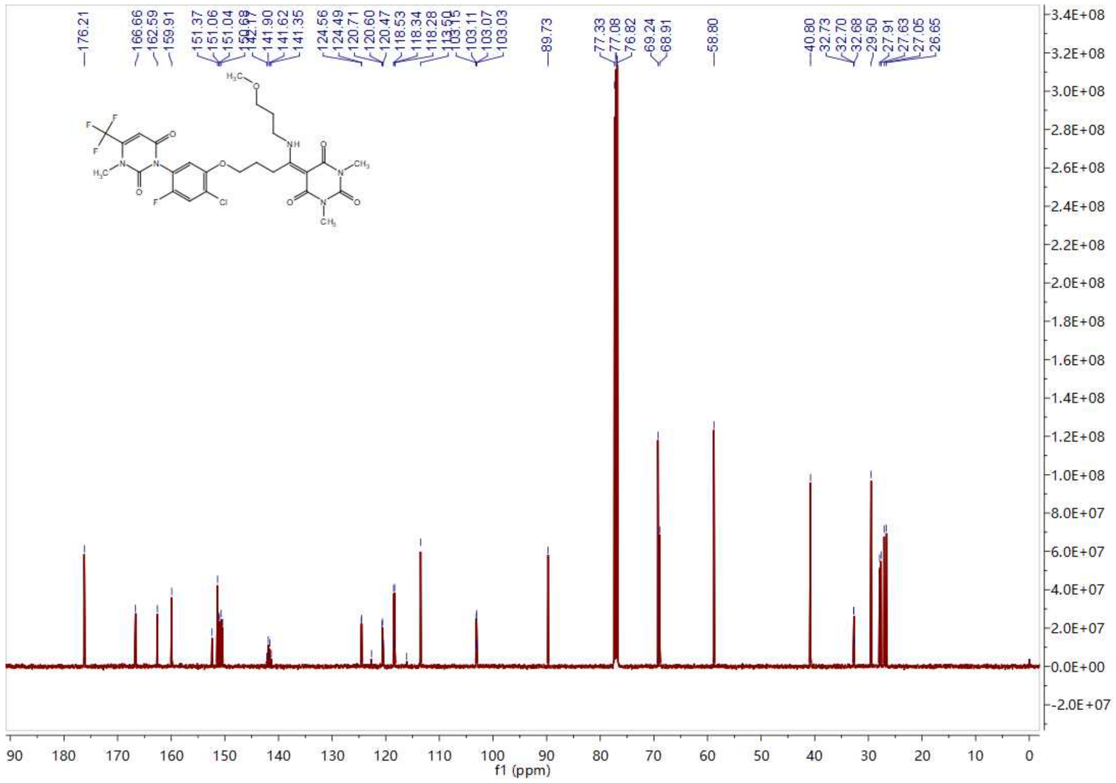

Figure S53 ^{13}CNMR of the target compound BA-18

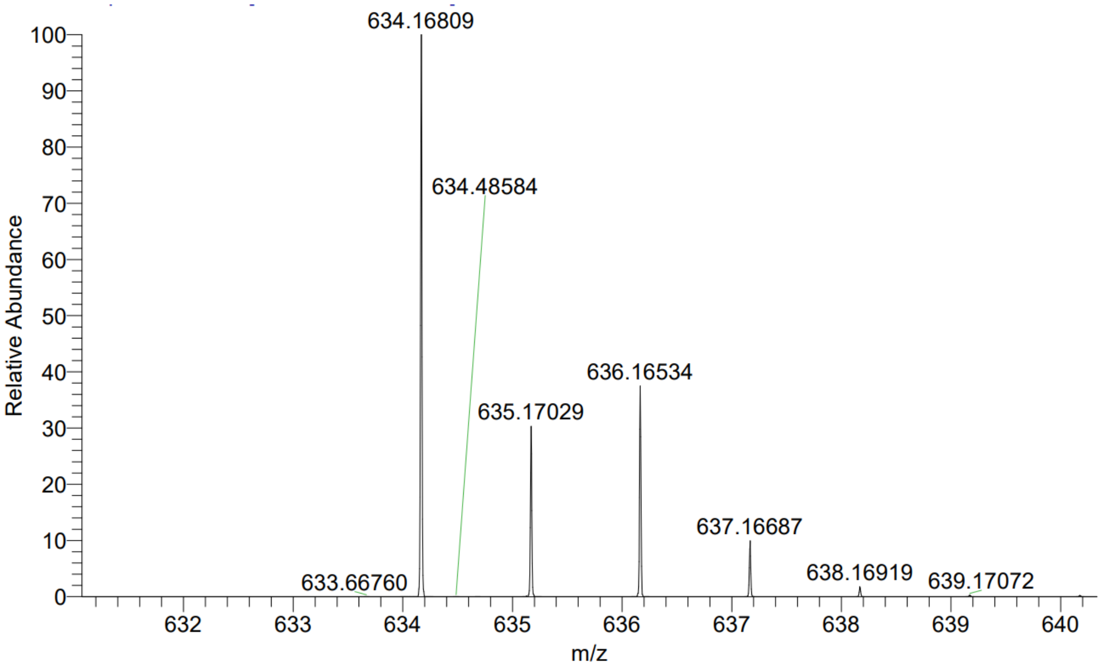

Figure S54 HRMS of the target compound BA-18

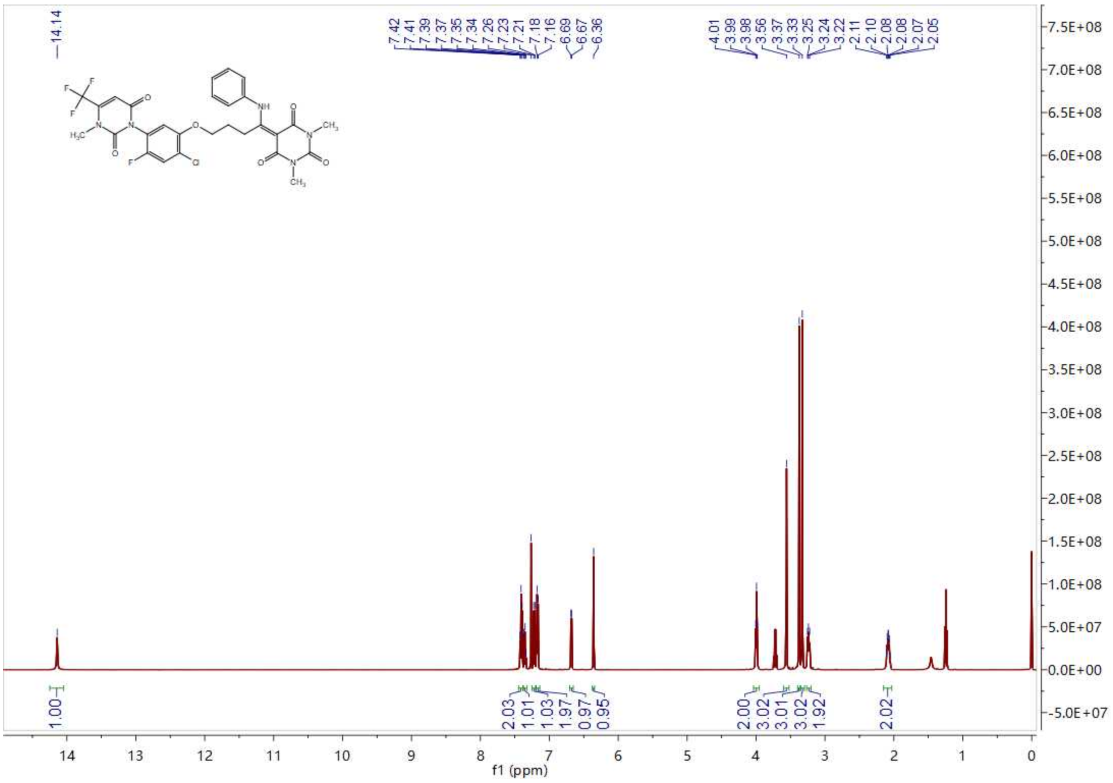

Figure S55 ^{1}HNMR of the target compound BA-19

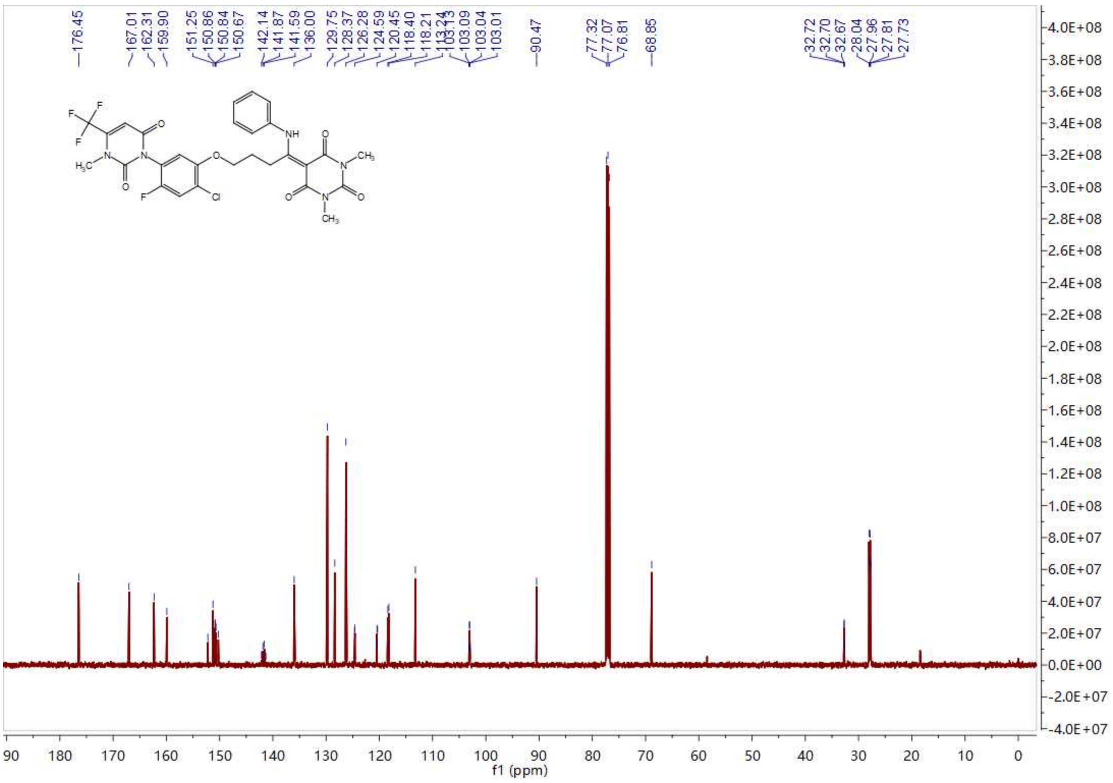

Figure S56 ^{13}CNMR of the target compound BA-19

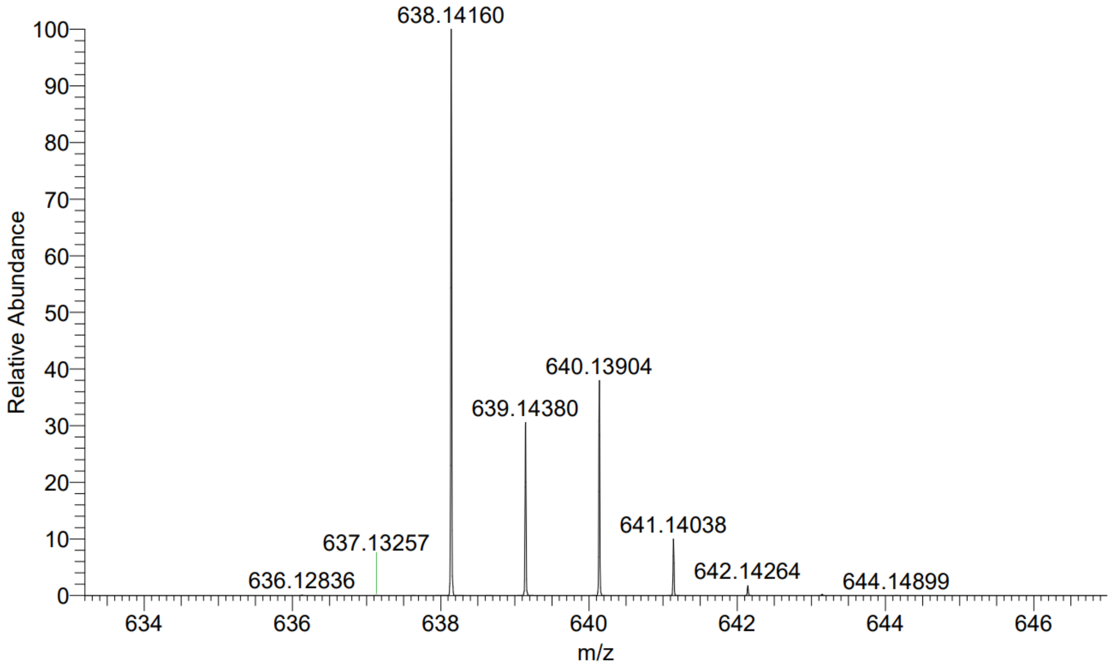

Figure S57 HRMS of the target compound BA-19

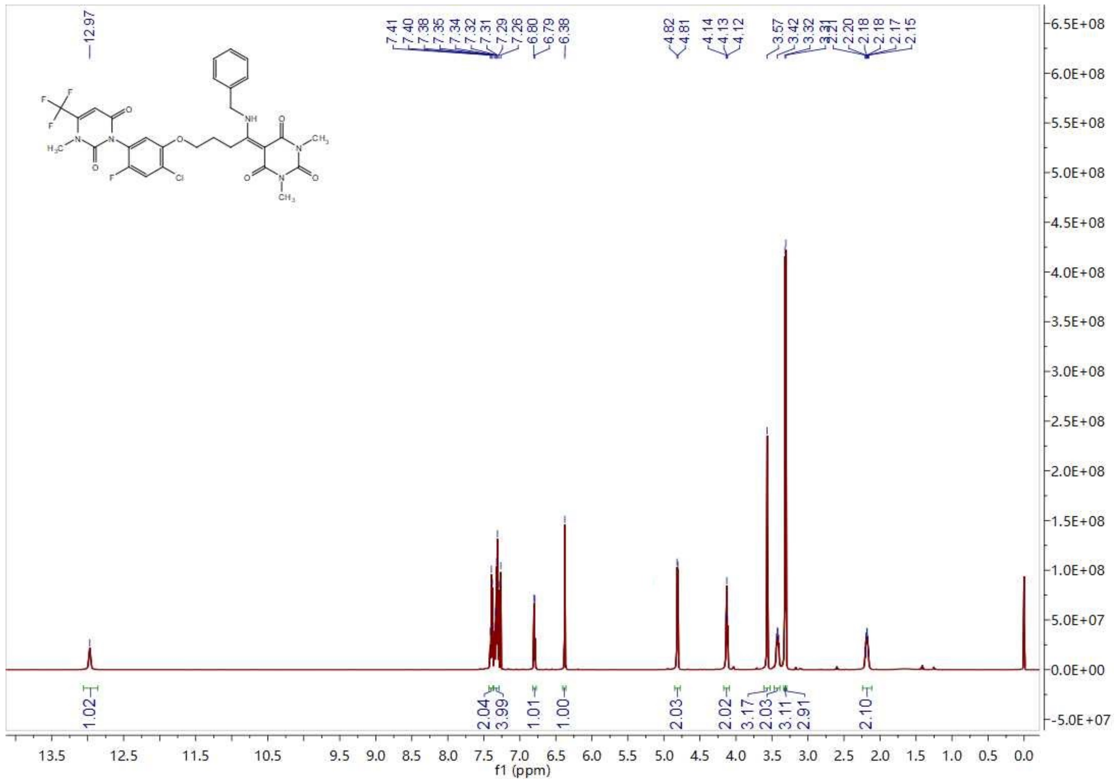

Figure S58 ^{1}HNMR of the target compound BA-20

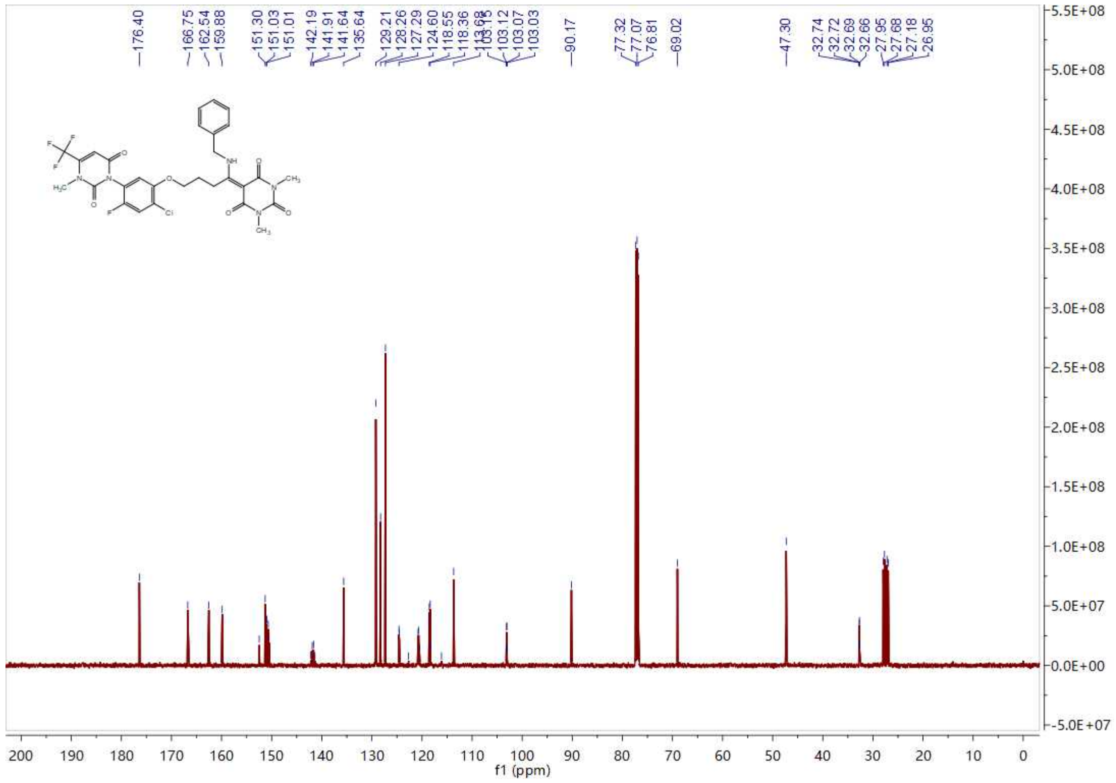

Figure S59 ^{13}CNMR of the target compound BA-20

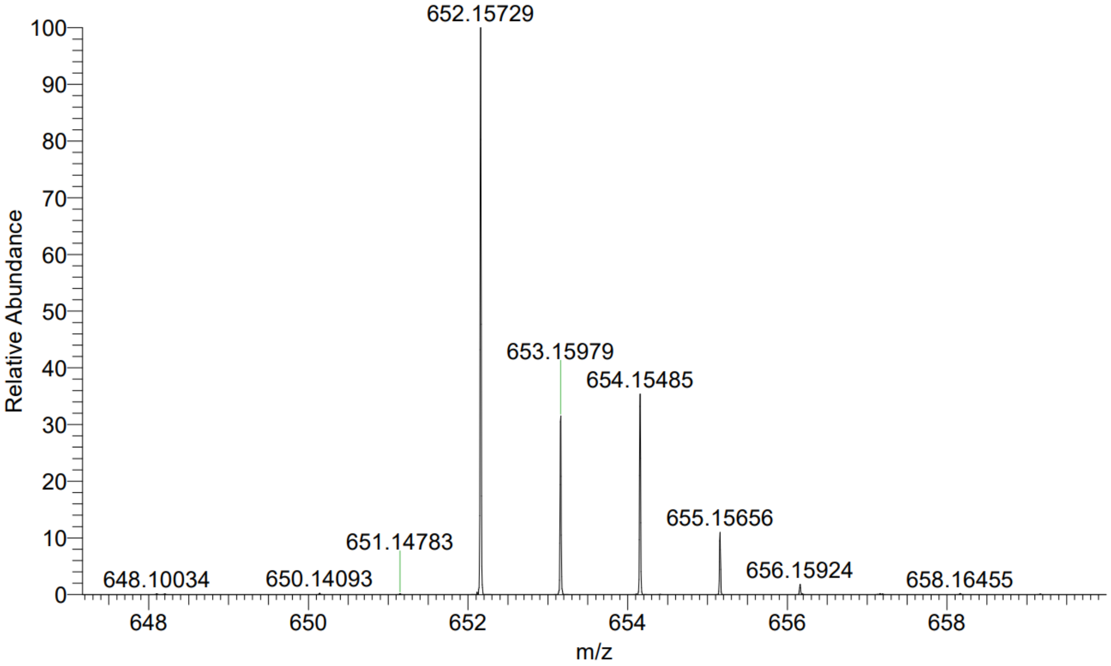

Figure S60 HRMS of the target compound BA -20
